# Supplementary figures and images for: The bacterial strains JAM1T and GP59 of the species Methylophaga nitratireducenticrescens differ in their expression profiles of denitrification genes in oxic and anoxic cultures
Source: PeerJ. 2024 Oct 28;12:e18361. doi: 10.7717/peerj.18361 (PMC11526790; doi:10.7717/peerj.18361)

GP59 «AN» | JAM1<sup>T</sup> «AN»

Cystovirus RNA -  
genome (?) -

23S rRNA -

16S rRNA -

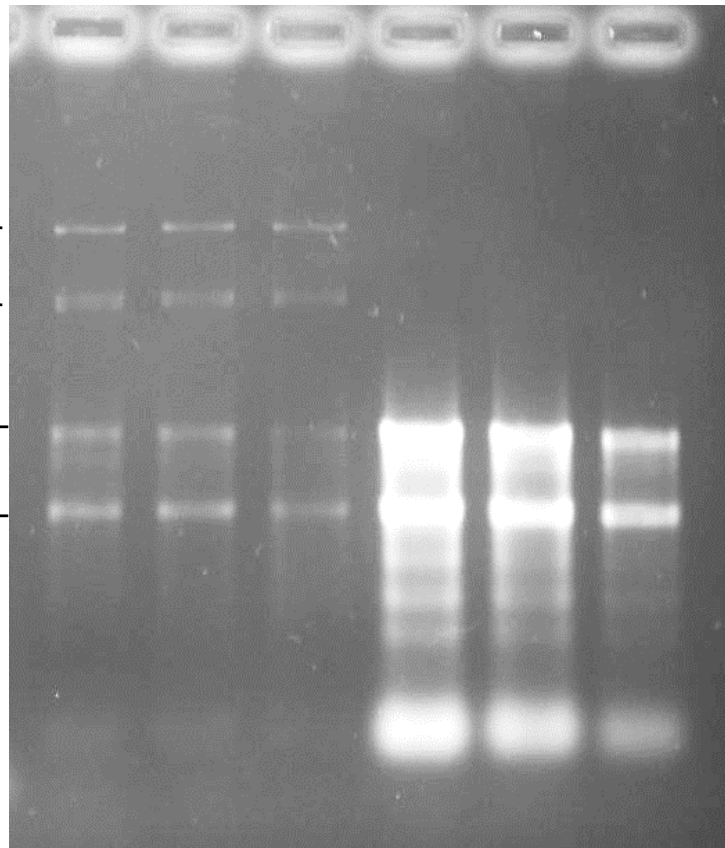

Supplement: Figure S1 — RNA extracts from replicate cultures were segregated by agarose electrophoresis and revealed by ethidium bromide and UV. DNAse treatment did not impact the high molecular weight transcripts of strain GP59 RNA, which may represent the Cystovirus genome detected in the transcriptomes of strain GP59 cultures. [file peerj-12-18361-s001.pdf]

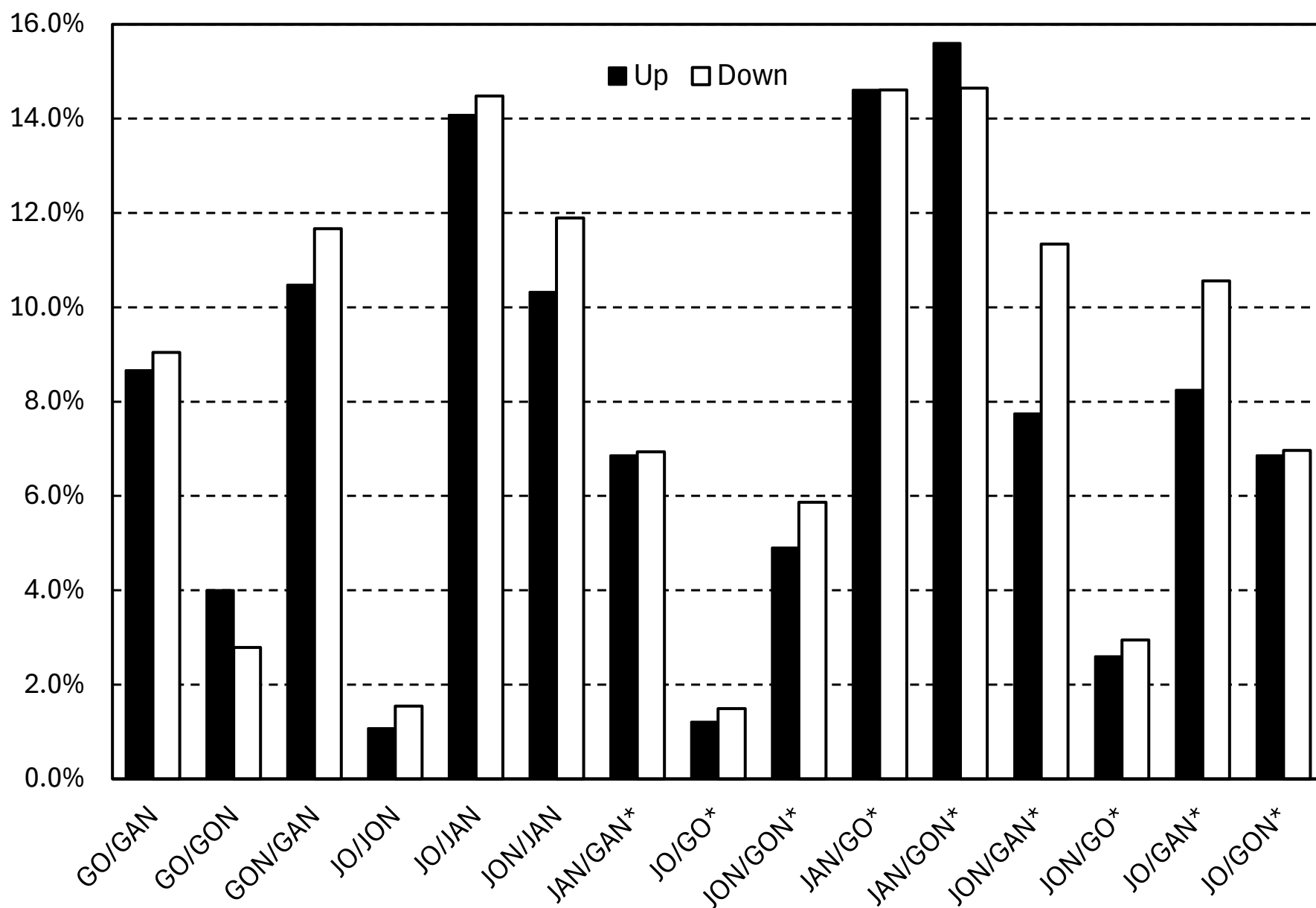

Supplement: Figure S2 — Percentages of genes and riboswitches where the fold changes were >2 with FDR < 0.05 in the relative transcript levels between culture conditions and between strains are illustrated. In GO/GAN, for example, “Up” refers to genes with relative transcript levels higher in the < > conditions than those in the < > conditions, and “Down” refers to the opposite. *: Comparisons between strain JAM1T and strain GP59 involved the 2813 common genes and riboswitches. G: strain GP59. J: JAM1T. O: < > conditions; ON: < > conditions; AN: < > conditions. [file peerj-12-18361-s006.pdf]
