# Supplementary material for: The bacterial strains JAM1T and GP59 of the species Methylophaga nitratireducenticrescens differ in their expression profiles of denitrification genes in oxic and anoxic cultures
Source: PeerJ. 2024 Oct 28;12:e18361. doi: 10.7717/peerj.18361 (PMC11526790; doi:10.7717/peerj.18361)
Supplement: Data S1 [file peerj-12-18361-s007.docx]

**Growth and NO_3_^-^ and NO_2_^-^ reduction under oxic and anoxic conditions.**

Strain JAM1^T^ and GP59 were cultured under three conditions: anoxic with 21.4 mM NO_3_^-^ («AN»), oxic with NO_3_^-^ («ON»; 21.4 mM NO_3_^-^) and oxic without NO_3_^-^ («O»). OD_600nm_, and NO_3_^-^ and NO_2_^-^ concentrations were measured at different time intervals. Each point is the average with the standard deviation of triplicate cultures.


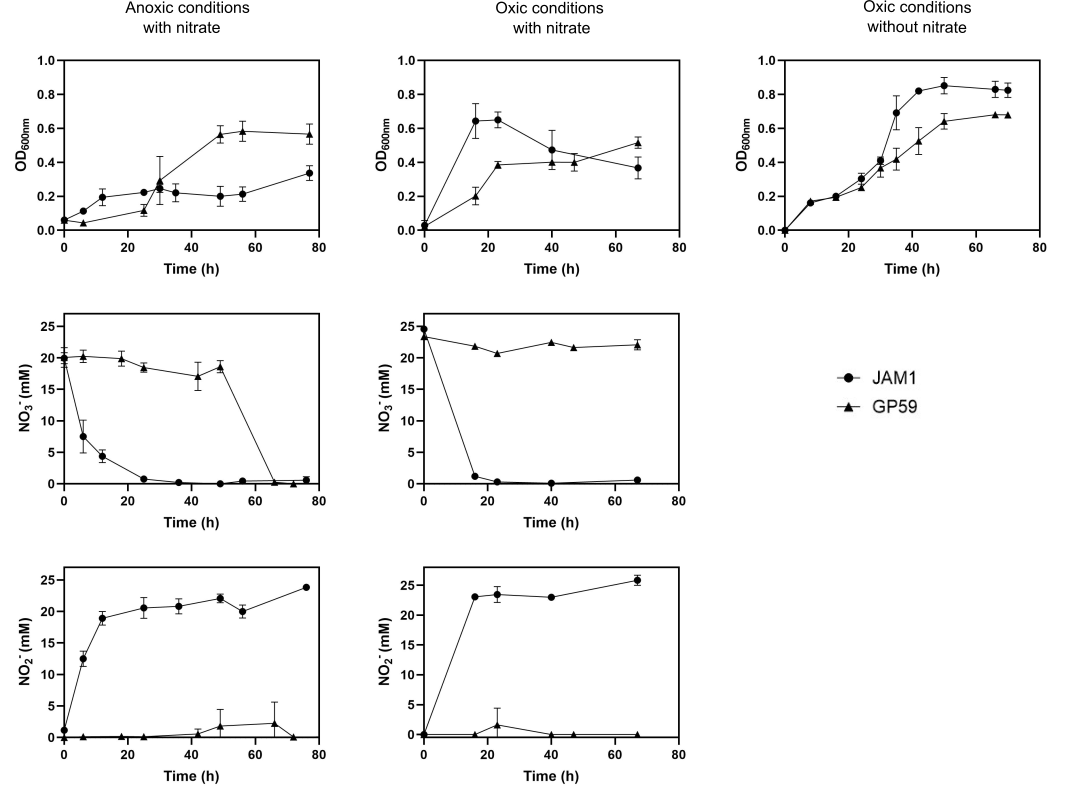


Strain JAM1^T^ grew immediately with no apparent lag phase under the «AN» conditions, and with a growth yield of around 0.2 OD_600nm_. Under the «ON» and «O» conditions, strain JAM1^T^ reached a growth yield between 0.6 and 0.8 OD_600nm_ after 24h. The complete reduction of NO_3_^-^ was observed within 20 h with strain JAM1 cultured under the «ON» or «AN» conditions, with accumulation of NO_2_^-^.

Strain GP59 showed a 24-48-h lag phase before growth occurred under the «AN» conditions. Complete reduction of NO_3_^-^ and NO_2_^-^ was achieved within 70 h. The maximum growth yield was 3 times higher (up to 0.6 OD_600nm_) than what was found with strain JAM1^T^ cultured under the same conditions. In previous work (Geoffroy *et al.*, 2018), we showed that strain GP59 reached higher level of growth (1.0 OD_600nm_) under the «AN» conditions with 42.8 mM NO_3_^-^, whereas strain JAM1 still reached the same level of growth (0.2 OD_600nm_). Finally, under the «O» and «ON» conditions, strain GP59 grew with no apparent lag phase, and reach approximately the same level of growth than strain JAM1^T^ cultured under the same conditions No reduction of NO_3_^-^ was observed with strain GP59 cultured under the «ON» conditions.
