# Supplementary material for: The bacterial strains JAM1T and GP59 of the species Methylophaga nitratireducenticrescens differ in their expression profiles of denitrification genes in oxic and anoxic cultures
Source: PeerJ. 2024 Oct 28;12:e18361. doi: 10.7717/peerj.18361 (PMC11526790; doi:10.7717/peerj.18361)
Supplement: Data S2 [file peerj-12-18361-s008.rtf]

Chromosmic region representing the Denitrification Island and sequence surrounding nirK


Sequence alignment of the denitrification island (position 763 to 67353: 66591 nt long) of strain JAM1 (GenBank accession number CP003390.3) and strain GP59 (CP021973.1). 44 substitutions were found, no gap. Open reading frame are colored. Dot represents identical nucleotide between both sequences. HP: Hypothetical protein. Fnr: putative fnr binding sites. Number locations are from strain JAM1 genome.


JAM1            TTAATAAATATTGTGGGACCAACGGTCTTACTATCATGCTGTTAAAAATAGGTGGAAAGAATGGAAACTTCCGCATATCAGAATCTGAAGCTAACTATTC 100   434083
GP59            .................................................................................................... 100   

JAM1            TTGACGTCCTGGGCCTTTCCAAAGATGCCATTCATATACATGTAGGTTTGCTGGTGTTCTTCCTGGCTGTAGTACTGTGGCGGCGCGGGCGGTTCGATGT 200   
GP59            .................................................................................................... 200   

JAM1            ATTAGCTTTGTTTCCAGTGTTCTTTGTGGCAATTACAATGGAGATTTTAGATTTACACGACGATTTGGGATCGCTTGGCTATATGCGCTGGTCGGCAAGC 300   
GP59            .................................................................................................... 300   

JAM1            CTTCATGATCTAATAAACACTTTTTTCTAGCCTACACTTGTTGTAATTTTAAGAAAGTGGTTGGGAAGTCGGGAGAACTTATAATAATGCGATACACCCA 400   
GP59            .................................................................................................... 400   

JAM1            ATACCAAACTTTCACTGTGCTGGAGTTTGTCAACTGAAGTTGCCTTTAGTTATTGTCGGTATCTAATGATGAGTCTATTTGCATTTATCGCTTATGCAAT 500 
GP59            .................................................................................................... 500   

JAM1            TGATAAATATTTATTGGTTAACTGTTTTGTTGAATTTAGCTGGTTTGTTTTGGCTTCATACAGAAAAAGGAACAAACTTCATGAATAGTGGGGTTTTTTC 600   434583
GP59            .................................................................................................... 600   

JAM1            ATTATTCAATGGCTAACAAACCTGAACTCGGGATAAGAAAATGAACTAAGTGCCTGCTTACGGATCAGATCTTTCGAGCAAGAATAATTTGATCACAAAG 700   
GP59            .................................................................................................... 700   
                                                                                763 
JAM1            GCAAGCAGGCATGGACAAGTTAGTTGAAATTTTCTGTGAAGTCGATGATTTTTGCCAGAAAT//TTATTCCTGTCTGGCAGCAGGAATTGCTGAAAAACGGA 800  <= narQ 
GP59            ..............................................................//...................................... 800  => HP/Mobile element

JAM1            GATAAACGTCGGAATAGGCCAGGTCGCTTATCAGAAAGTGAAGTCATAGTGAAGCTCTGGTTATTTAGAAATAATTATCAATTATGCGCGAAATAGTTTT 900   
GP59            .................................................................................................... 900   

JAM1            CAGTGTTTCGGCGTCATCGGATAAAGGTTCTGCCAGGCAGGAATAACAGGCCTGTTTCGGATCCACACCGGATTTAATCATAATTGCACAATAAACCAAG 1000  434983
GP59            .................................................................................................... 1000  

JAM1            AGACGGGTTGAGGGAGCTTCTTCCAGATCGTGTTCCTGTAAGCGCCGCAAATCTTGTGCGAGACTAATTAAAAGTGAAGCGGTCTGCTTTTCTATGCCCG 1100  
GP59            .................................................................................................... 1100  

JAM1            TTTCCTGACACAAGATAACGATTTCATGATCTGTTTGGGGATAATCAAAGCTTAGAGATACAAATCGTTGTCGGGTACTGGGTTTTAAGCCTTTCATGGC 1200  fnr
GP59            .................................................................................................... 1200  

JAM1            ATTCTGATAACCCGGGTTATAAGACATGACTAACATGAAGTTGGGATGTGCCTGGATTAATTCTCCTGTGCGTTCCAATGGTAGTTCGCGGCGATGATCA 1300  
GP59            .................................................................................................... 1300  

JAM1            GAGAGTGGATGCAGTACAACGGTAGTATCTTTACGTGCTTCTACTACTTCATCAAGATAACAAATAGCGCCTTCCCGTACAGCTCTGGTTAGCGGCCCAT 1400  
GP59            .................................................................................................... 1400  

JAM1            CCTGCCAATATGTGCCACTGCCATCGATCAAGTGACGTCCTACCAGGTCTGTTGCTGACAAGTCATCATGACAGGCGATGGTATATAACGGTCGTTCTAG 1500  435483
GP59            .................................................................................................... 1500  

JAM1            TTGTTCCGCCATATATTGAACAAAGCGGGTTTTGCCACAGCCAGTAGGCCCTTTTAATAAAACCGGTAAGCGGTTATTTGCTGCTGCCAGGAAAATCTCG 1600 <= norQ 
GP59            .................................................................................................... 1600  

JAM1            ACTTCATTGTTCTGTGAAATATAATTTAAATTAGCCATAGAAAGGATTCATCCATTTTAGTTTTTGGTCAGATTCAGATACAGCCGGGGCAGTAGTATGG 1700 <= norD 
GP59            .................................................................................................... 1700  

JAM1            GTAAACGTGCTGGATCGCTGATTAGACAAAAGCCCTGTTCGCCAAAAAGATAGGGCAGGTATTTATTTGCTTCGTGGTCAATAGTGATACAGAAGGGTTT 1800  435783
GP59            .................................................................................................... 1800  

JAM1            AATCCCTTGCTTTTTTGCTTCAAGAATTGCCTGGCGCGTATCTTCGATACCGTAACGACCTTCATAGTGATCGATGTCGTTTGGTTTACCATCGGACAAA 1900  
GP59            .................................................................................................... 1900  

JAM1            ATCAGCATTAACTTCTGCTGGGTTTTTTGCTCTTTCAGTATTTCTGTCGACTGGCGGATGGCGGTCCCCATACGGGTATAATATCCAGGCTTTAATGAAT 2000  
GP59            .................................................................................................... 2000  

JAM1            GAATTCGCCCATAAACACCATCTGACAAGGGTTCATTAAAGTTTTTCAGCAGGTGATAACGGATTTGTTTGTTTTTAACGGATGAAAAACCGTAAAGTGC 2100  
GP59            .................................................................................................... 2100  

JAM1            AAAATGGTCACGACTGGATGCCAATGCCGAGGAGAAAATGACTAAGCTGTCCTTGATGACATCAATAACTTTTTGTTCATCGTTAATAGCGGCATCTGTC 2200  
GP59            .................................................................................................... 2200  

JAM1            GACATGGAAATATCTGCCAGCAAGAGACAGGATAAATCACGGTGATCTTTGGAGAGCCGACGATAACAATCTGGCATATCGACGGATTGTGTCGGGTTGG 2300  
GP59            .................................................................................................... 2300  

JAM1            ACAAATGATCTATCCAGGCATTTAAATCCAGCTCTTCGCCTTGAGACTGTCGGTTTAACCAGCGTCTTTCCGGTATTAACAATTCAAATTGCCGACAAAT 2400  
GP59            .................................................................................................... 2400  

JAM1            TTTAGCGGCTAGAGCCCGCAGATGAACCGGCATTGGCGTGGGGGGGGCTTCATCGGCTAAAAAGGGTTGCAACAGACAAAAGTTGTCTCGCATTTGTTGC 2500  
GP59            .................................................................................................... 2500  

JAM1            TTTTTATAATCCCACTCAGGCAGCCGAATGCCTTCTCCTAAAGGTAATTCATCATGTATTGGAGAGGGAAGATCCATATCAAAGCGTATTTTAGCTGCTC 2600  436583
GP59            .................................................................................................... 2600  

JAM1            CGGCTCTGCGCTTTTTCGACAATGTGATGACATCCAGATCCTCCGCAGCGCTTTTGGCATCCTCATCCATATCTTCTTCTTGAGGTCGATCAACTGGAAT 2700  
GP59            .................................................................................................... 2700  

JAM1            ATGCTCTGTCCAGCTGAATAAGCTCTCAAGCCGAAAAATTAAAAGTCCGTCCGTTTCTTTACTGTCATCTTCACGATTGGCTTGTTTTCGTCCGGCATCA 2800  
GP59            .................................................................................................... 2800  

JAM1            GCTTCTTCAATAGCGTCTGGTTCGGATGAACTTCCTTCCTCAGTTTCTTCTGCCGCTATGAAGTCTGTGGGCATCCAGGGGGAAGGGTATAGCCATAAGG 2900  
GP59            .................................................................................................... 2900  

JAM1            CAACAGGTAAAGGATGAAGTTTAGATTCTATCAGCTGTTTAACAGATCCAGGCTCGCTGAGTGCCTGTTGAATTTGTATTTCTTGTTCGTTTATTGCAGA 3000  
GP59            .................................................................................................... 3000  

JAM1            TAGCTTTTTCCAATAACGCTTGCGTTCATCAGAAACCGCTTCTACTAACTCGTGATATCGGCGCTTTAGTCCCGGTCTTTTTTTGAGTAGTTGTAAGGTG 3100  
GP59            .................................................................................................... 3100  

JAM1            GCAAATTGGTTATCAGTAAACCAAGAGTTTATATTTGGCATTTGTGCTGCCATAGCCGTAAGCCAAAAATAGATATCTTCATTAAGGCTGGTATCGGTAC 3200  
GP59            .................................................................................................... 3200  

JAM1            AAATGGATACTGTTGGAGGAAGTCGCAAACAACGTTCATCTTGCCAGGCTAGGTAAAAGCGTTGCTGACTACCGGCAACCTTATCGATAAAACGGCGATG 3300  
GP59            .................................................................................................... 3300  

JAM1            GATTTTGACTTTATGGGTATTGGCTGCCTCTATAGACTTGCCTGGGTCTCCACCCATTGCGCGGTAATAAGTAGCCAATTCACTCTGAAGGTCCGAAAGC 3400  
GP59            .................................................................................................... 3400  

JAM1            GATACAGTGGCTTCTTGATGGTTGATATTTGCTTTCTGGCTGATGAGTTTATGCCAGACTGTTCCAACGTACTCTTCCATATTTGACTCTTCCCAGACGA 3500  437483
GP59            .................................................................................................... 3500  

JAM1            TAATAAACGGGGTTTTGGATGACTCCAAAACCCCTGCTTATCACAACATCATGCAGGATTGGTAACGACTTTCGCTTCGGTATCTGAAACGGTATCACCA 3600  <=norD;<= norB
GP59            .................................................................................................... 3600  

JAM1            CCGACAAAGAAGCTTGCAAAATAAGCAATCAAGCCTCCAAGGAACAACAGGCCAAAGCCGAGACGTGCCCAGTAGACAGGACTGATAGCTTCCTGTGTGG 3700  437683
GP59            .................................................................................................... 3700  

JAM1            CCATAAATGCCAGAGAATTAGCATCATCAGGAATACGTTGTAAGATTATTTGCCATACACCGGCAGCAGTTAATGCCAGCGTGATACCTATCATGGCAAT 3800  
GP59            .................................................................................................... 3800  

JAM1            ACACATCGCCCAGAAAGCAAAGCGTTCTAGTTGTTGTGCCTGAGGTGAGTTACCCTGAGGCCGTCCTCTCATAATTGGCATGGCATAAGAAATAATACAC 3900  
GP59            .................................................................................................... 3900  

JAM1            ATTACAACCATCGCATAAGCCCCAAAAAAGGCCAAGTGACCATGTGCTGCTGTAAGTTGTGACCCATGGGTATAGTAGTTGACGGGAGCTAATGTATGTA 4000  
GP59            .................................................................................................... 4000  

JAM1            AAAAGCCCCATACACCTGCACCCAGGAATGCCATAACAGCTGTGCCTTTCGCCCACAATGTTGCTACTTCGTTCTTATGGTGAATCCGACGTTGTTCAAC 4100  
GP59            .................................................................................................... 4100  

JAM1            CATTCGGAAAGCAAACAGCATCATCATGAAAAATGGAACAGGTTCGATAGCAGAGAAAATTGAACCTAACCACAACCAGTATTCGCCAGGGCCAATCCAG 4200  
GP59            .................................................................................................... 4200  

JAM1            AAATAGTGATGTCCCATACCGATTAAGCCGGAAATCAATGCCATAGCGATAATGACATATAACCATTTTTCAATAATTTCACGGTCGACGCCAGTGACTT 4300  
GP59            .................................................................................................... 4300  

JAM1            TAATCAATACAAAGGCAAGAATAGCACCCATGATCAATTCCCAGACACCTTCAACCCAGAGATGAACAACCCACCACCAGAAGAATTTATCCAGTACCAG 4400  
GP59            .................................................................................................... 4400  

JAM1            GTTTGACGGGTTATAGAATGAAAACAAAAACAAGAGAGCCAAACCGATCAGGCCGGTAATCAATACCATATTAACGACGGTTTTACGGCCCTTGAGAATG 4500  
GP59            .................................................................................................... 4500  

JAM1            GTCATTCCTATATTGAATACGAATGCCAGAATAACCACAACCAGTCCAATTTTTGTAATAGTTGGTTGTTCCAGAAACTCACGTCCCATTGTTGGAAGCA 4600  
GP59            .................................................................................................... 4600  

JAM1            GATCATTCCGGGTTAGGCGGGCAAGCTCAGAATAAGGCACCAGCAGATAACCCAGAATTGTGGCGACCCCCGCTGCTGCAAAGACCCAGAAAGTTATTTT 4700  
GP59            .................................................................................................... 4700  

JAM1            TGCCAATAACGGACTGTATAACTCCGTTTGTGACTCTTCGGGTACCAGATAATAAGATGCTCCCATAAAGCCGAATAGTAACCAGACAAGTAATAAGTTG 4800  438783
GP59            .................................................................................................... 4800  

JAM1            GTATGCACCATTCTGGCAACATTAAAAGGTATGTATAGGGATAAAAAGTCACCCACAACATACTGCAAACCCATAATCAAACCAAACAGGATCTGTCCGG 4900  <=norB
GP59            .................................................................................................... 4900  

JAM1            CGAATAATATGAGTGCGAAAATAAAATACGGTTTTGCCACCGCCTGAGATTGATAATTCATTACAAAAACTCCTTCTTTAACCTTGAATATTAGGCGGCC 5000  <= norC
GP59            .................................................................................................... 5000  

JAM1            AGTTATTATTTTTGATACGAGAAGTCCAGATAAGAAAATCTGACAACGCATCAACTTGTTCATCAGTCAGGTTGAATTGGGGCATTTGACGACGACCTGG 5100  
GP59            .................................................................................................... 5100  

JAM1            TACATTCAACGGTTGGGCTGCCATCCAGCCACGCATAAACCCTTTAAAAATTTCTTCATCACCATTACCATAGCGATCAAAAACATTCATGAGCTCTGGC 5200  
GP59            .................................................................................................... 5200  

JAM1            GCGTAATAGGCTCCTTCGCCCATAATGGAATGGCAACCCACACAATTATTGGCTTCCCATAAGTGCTTACCCTCAACGACAGACTCGGTAATTAAATGTC 5300  
GP59            .................................................................................................... 5300  

JAM1            GGTTATCCCGTTCTGGTAAAACCAGCATCGTGTCAAAAGTCAGCCCCAGTAAGAGCAGGATAAAAAAGAAGCTGCCGCCGTAATAGATATTACGGGCCAT 5400  
GP59            .................................................................................................... 5400  <= norC

JAM1            TCCTTTGTTAAAAAATTCAGTCGCCATGGTGTCTCCTCATACCGTTTATCTTGATTGATAAAAACATTTTTTGTTGTTCTCCGACTTAAAATAAGATGCT 5500  439483
GP59            .................................................................................................... 5500  

JAM1            ACCCAGAAAGACCATAGCCAGAATCATGCCAAATAGTTATGACTGTCTATTGTATAGCGAAACTGCTCGCTAAACGAAGTGTTACAAGCACTTTTGTGAT 5600  439583
GP59            .................................................................................................... 5600  

JAM1            TATTCCGGCTTTGGCTGATATGATGAAGTTGACGATTTCTAAATAAGCAGCTCAGCATGTTGTGATTTAGACATCACTGATAATGTTGTTAAAATAACAA 5700  
GP59            .................................................................................................... 5700  

JAM1            AAGTTGTGCTTATGACATCACCCGTTTTTTATAAAGCGCTTATTCGCCTGGTTTCTGATCTTTCCAGCGATCTTCCCGCTGAGGAGCGCTATAGCCGCTT 5800  => norR
GP59            .................................................................................................... 5800  

JAM1            ATTACAAGAAGTTGTAAATATTTTTCCTTGTGATGCCGCCGCCTTACTCCAATTTGAGAATGGTTATCTAAAACCGTTGGCAGTAAGAGGGTTATCAGTG 5900  
GP59            .................................................................................................... 5900  

JAM1            GATACAATGGGCAGGCGTTTCAAAGTTGATGAACACCCGCGGCTTCAAAGGCTTTTACATTCCCGTGACCCTGTCCGGTTTGCTGCAGATAGTGAGCTTC 6000  
GP59            .................................................................................................... 6000  

JAM1            CGGATCCATATGATGGTCTTGTCGAAAATGCTGGACATAAGTTACATGTTCACGATTGCATGGGGGCTTCTTTACATATAAATAACAGGCCCTGGGGAGT 6100  
GP59            .................................................................................................... 6100  

JAM1            TTTGACGCTAGATGCAATGGATCCTCGTCGTTTTGATCAAATTGAACCGACGATTCTGAAAACCTTTATTGGTCTAACGGAGGCGACAGTAAAGGCGGCA 6200  
GP59            .................................................................................................... 6200  

JAM1            GGCCGTATTGCGGCATTAGCCGCCCGCGCTGAACATGAACATCAAGTTGCTGCAACATTGCAAGAACAAAACCAACAGGTAGAAATAGTAGGAAGCAGCC 6300  
GP59            .................................................................................................... 6300  

JAM1            CTGCCATTGAAAAGTTATGCAATGAAATAGATGTGGTGGCACGGTCAGATCTGACAGTACTGGTTCTGGGGGAAACCGGTGTCGGTAAAGAATTGGTGGC 6400  440383
GP59            .................................................................................................... 6400  

JAM1            ACGGTCTGTGCATAACAAATCTCAACGTAATAAAAAATCATTGGTTTATATCAACTGTGCAGCGTTACCTGAAGGCATTGCCGAAAGTGAGTTGTTTGGT 6500  
GP59            .................................................................................................... 6500  

JAM1            CATGTAAAAGGTGCTTTTTCCGGCGCAACCAGTGATCGTGCCGGTAAGTTTGAAATTGCACATGGTGGAACCTTGTTTCTGGATGAAATTGGTGAATTAC 6600  
GP59            .................................................................................................... 6600  

JAM1            CACTGTCTCTTCAAGCGAAATTATTACGGGTTTTGCAAAGTGGTGAGATACAGCGAGTGGGCAGTGATCGACATAAAGAAGTAGATGTACGAATTGTTGC 6700  
GP59            .................................................................................................... 6700  

JAM1            TGCGACAAACCGTAATCTGCTTGAAGAAATTGAACGTAAGCAATTTCGGGCCGACTTATATCACCGCTTGTCTGTTTATCCGATTACCGTACCACCGCTA 6800  
GP59            .................................................................................................... 6800  

JAM1            CGTGATCGAGAAAACGATATATTGTTGCTGGCGGGATATTTTATGGAGGCAAATCAACGTCGGCTGGGTGTGAGGGGGCTTCGGTTATCACAGGCGGCTC 6900  
GP59            .................................................................................................... 6900  

JAM1            AGCAGGCTTTGCTCGCTTATAACTGGCCCGGAAATGTGCGAGAGCTCGAACATTTAATTAGCCGGGCTGCGTTAAAAGCAATTGCCGGTAAAAAACATCC 7000  
GP59            .................................................................................................... 7000  

JAM1            GGATCCGGTGATATTGCAGGAAAAGAATTTAGGTTTGCACCTCGTGGATTCTGATACGGATTCTGTTGAGGAAGAATTAATAACGGGGTTAACACCAGAA 7100  
GP59            .................................................................................................... 7100  

JAM1            TGGAGCCGGTTTAGTCATTTTGACAACGTCCGAGATGCACAGGATGAGTTTCTCAGATATCTGATCCAAGAAAGGTTGGACCAGTTTGATGGTAATCGAA 7200 => norR fnr
GP59            .................................................................................................... 7200  

JAM1            GTGCTGCAGCGCGTTCTTTGGGGTTAGACAGAGGAAATTTTCATCGTTTGATGAAACGATTAGAGTAATTAAAAAGTAATTATCGCTGGAGTGTTTATGT 7300  =>norE
GP59            .................................................................................................... 7300  

JAM1            ATCAGGGTCTCAGTAGCAGACAAGCAAAAGTTTTGCCGGGTAATGCTGGTGTTTGGTTATTCATTTTTGTAGAGATTACGACGTTTGCGTTATTTTTTCT 7400  441383
GP59            .................................................................................................... 7400  

JAM1            GACCTACTCCGTTTTGTATCGTCTAAATGGCAGTTCGTTTGAGCAGTATAAAGCCGTCCTACATTTAAACTTGGGGGTTATCAACACCATCATGTTATTG 7500  441483  fnr
GP59            .................................................................................................... 7500  

JAM1            ACTTCCAGTCTGTTTGTTGCCTTGGCTGTCAATAAAGCGAAGATACAACAGAATACTCGTGTTGCCGGGTATTTGTTGCTGGCGATGATCTGTGGTTTTG 7600  	
GP59            .................................................................................................... 7600  

JAM1            TATATATAGGTACAAAATTCTGGGAGTGGCAGCAGCTTTATAGTATTGGCTATACCCTGCATACCAATACCTTTTTTAGTTTTTATTTCTTTCTGACTTT 7700  
GP59            .................................................................................................... 7700  

JAM1            ATTCCATTGGTTACACGTTATTCTGGCATTGATCATTCTGAATAATTTTCGCAGCCGTTATCAAAAAGCGGATCCACCACCAAGTCTGGAAGGGCTTCAG 7800  => norE
GP59            .................................................................................................... 7800  

JAM1            TCTGCGGGCAGCTACTGGCACATGGTCGACATGTTGTGGATCATTCTGTTTTTACTCATTTATATAGTCTGAATTAACAAAGAGTAGTTGATTATGAAAT 7900  => HP
GP59            .................................................................................................... 7900  

JAM1            TATGGTTTTATTGGTTATTTCTGATAACTGCAACGGGCATGATGGCCGCTTTTAATATTAAAAGTTTTCAATTGCTGATCTTGCTGTTAACAATTGTAAA 8000  
GP59            .................................................................................................... 8000  

JAM1            ACTGCTAACTATCTGCGAAGTCTTCATGGAAATGAATCACGCCCCTACGCTTTGGCGAAGTGCCATGCAACTGTATTCAGTGATGTTACCGGTAACGTGT 8100  
GP59            .................................................................................................... 8100  => HP

JAM1            TTTTTCATCATTAATTTTTGATATGGATTGTCCAAGATCAGGGTGCCTTACAATGTAATTTGCTGATCTGGGTGGTCATTATTAAACAAGTATAAAAATG 8200 <= nnrS
GP59            .................................................................................................... 8200  

JAM1            ACAAAATCGGATATTTTTGTTTCATGTGTTTAACGGAAACTAGCCTGCTCGTCCATCAGCTCGAGGGTTCAAGTAGATGGGGGCATATTTGTAGCTCCAT 8300  
GP59            .................................................................................................... 8300  

JAM1            ACGCCAAAACACAGTAACCAGAGCGATCCGGCAGCAAGATACAGCCAACCGGTGAAAAGAGACGATATACCGGGGATATCCGCTGCTATCCTTAAGATGG 8400  
GP59            .................................................................................................... 8400  

JAM1            TGACATACTGAAAAGACAAAAAAAGTGCCCAGCTGGCCATGTCAAGTATTAGCGGCCGGCCGGAATGTCCCAGTGTGACTCTGGTTATCATGGCTAACAG 8500  442483
GP59            .................................................................................................... 8500  

JAM1            CATTGAGCTGAAATAACCAATCACGAGTGCGTGCAGTGGCGCTCTGAGTAAAATGTATTCTCCAGTCAAAAGCAGATAAAAGCTCTGAAGAGTATAGAGC 8600  
GP59            .................................................................................................... 8600  

JAM1            GTCATGGCGATACCAAACCAGACAAAGCTGATGTGCAACATGGCTAAAAGCGGGTGGGCAAGGCTCTTAAAACTTTGCCATTTGACTGTCAGGTAAAAGC 8700  
GP59            .................................................................................................... 8700  

JAM1            CAATAATGGCCATCGGTAAGTCAACCAGCCAAAGCCAGGCATAAAGATTACCCATTTCCAAGATCGCATGGATAATGGCAAACAACAGAACGAGTGATAA 8800  
GP59            .................................................................................................... 8800  

JAM1            AGCCCGGTATGGTCGGATAATGTCATAATTTGGCAGCACATTGGCGGAAAAAAAAGGAATCATCCGATGACTGACAGCAAAAAATAAAGGAAGCAGAAAT 8900  
GP59            .................................................................................................... 8900  

JAM1            AACCAGACACCACCAATTTTGGCCAATCGCACCATCTCAAAATGTCCTGAGATGAAACCGGCAATCATCACCCAGGCAATAACCAGCATTACGCTCGTAA 9000  
GP59            .................................................................................................... 9000  

JAM1            TGGTTGCGTGGCGTTTGTCGGGATGTTTAGCTCGCAGATAAACCTGCAACAAAGCATATAGTCCGAAAGACCATCCTATAAGAAAGATCACCAAAGCAAT 9100  
GP59            .................................................................................................... 9100  

JAM1            TGTTAGTATAAACGTGTTGCTGATGAGCCCGATATAAAACAATAAGATACCTGCATTCAGCAAAGAGAATGCTGGGATATACAGTTTTCTATCAACTTCT 9200  
GP59            .................................................................................................... 9200  

JAM1            TCGCCCTGCATCCATCGAGGGTAAGTAGTCATTAGAAAGCCAAAAATGAAAAAAGCAAAAAAACCATAAATCATCAAGAAAGCGTGCGCATCATTAGCCG 9300  
GP59            .................................................................................................... 9300  

JAM1            GAATTACCCAGTTAACCGGCATGTCAAAGTCGGTAAAGCGACCGCTTAAATCACTAAACCACCACAGCATGGCTAATAGTGCTTGCATGGTACCTGTAAA 9400  <= nnrS
GP59            .................................................................................................... 9400  

JAM1            AAAGAACAGACGATGTGGTGCGGATGAAAATATCTGCCAACGTTTATTCAGCTTTTGCATAATTAAATTCCGGTTATCTGTCGGCCCTGGAAAGCCGTAA 9500  443483
GP59            .................................................................................................... 9500  

JAM1            ACAGTATGGTTATCACAAAAAAGACTATTGCAAGAACATTCAAAAATCTACCCATACGCCTTAATGAGAAAACGACAGCCTAGTCGTCATATTTACGTAT 9600  
GP59            .................................................................................................... 9600  

JAM1            CAATAATGACAAATCTGAATGCGTAACTTTGGATTGTTTCAGTAATACCTCTACACACCATTTATCTGTCGAGACGTGATGGTAAACCTTTTTAGTAGCG 9700  
GP59            .................................................................................................... 9700  

JAM1            AATGATGCGATGAGCATTTATTTTCTGAAGCGTTTGTCGTTCAATGAAGAACTTTACAGTGATCAGGTTAATCGAAAATCCGTAGTTCTTCTACTTTATC 9800  
GP59            .................................................................................................... 9800  

JAM1            CTTAAAAAAACAGGACAACTTTGATAATGGCTAGCGGTTTATTAAGGCCGATAGAGAGCCGATATAGACTAAGATAAACAAGATAAAACGGTAATCGAAA 9900  
GP59            .................................................................................................... 9900  

JAM1            GAATAACCGATGCTAAGAATCCCACCGTTCCAGTAAGCGGAAGCGAAGGATAGTTAACCTCCCGTCATATTCATAAAACGAATTACTTTTGCAGGGCCTT 10000 <= moaA
GP59            .................................................................................................... 10000 

JAM1            CGATAGAAAAATTATGTTGTGCCGGCTTGACTTGCAAGGCATTAAGCAGGGCTTTCTGTATCGGTGCGTCTGTTGTTGGATAACGCCGGATCAGTGAACG 10100 
GP59            .................................................................................................... 10100 

JAM1            CATATCCAGAGAGTTTTCCTCTCCCAGACATAACAATAACCGACCTTCAGCCGTCAGGCGAAGGCGGTTGCAGGTGGCACAGAAACTGTTTGAATGAGGA 10200 
GP59            .................................................................................................... 10200 

JAM1            GAAATAAAACCAATGCGACTTTGTGAGTAACCTTTAACCCGAACATAGCGTGCCGGGCCACCGCTGTTTTCAGTGCTGTCGATCAGAAAGTGCTTTTGAC 10300 
GP59            .................................................................................................... 10300 

JAM1            TGATAATCGAGCGGACTTCTTCACTGGAGCAAAATGTCTCACCTCGTGAATGGCCGACATTTCCCAAGGGTATTTCCTCAATAAAGCTGATATCCAGTCC 10400 
GP59            .................................................................................................... 10400 

JAM1            CCGATCCAGAATGAAGTCAATCAGATCCGGTACTTCATCGGCATTGCGTCCTTTCATCACCACCACATTCAGTTTGATATGCTCAAACCCAGCCTGACTT 10500 444483
GP59            .................................................................................................... 10500 

JAM1            GCAGCATCGATACCCGCCAGAACCTGAGACAAGCTACCGGTTCGGGTAATAGCGTTGAATTTTTCCGCATTCAGGCTATCCAGACTGATGTTCAAACGTT 10600 
GP59            .................................................................................................... 10600 

JAM1            TTACACCAGCCTCAGCCAACGGTTTGGCCAGATTGGTTAGCTGCGAACCATTAGTGGTCATTACCAGTTCTCGAAGGCCAGGTAATTCACTTATTTTGGC 10700 
GP59            ...............................C.................................................................... 10700 

JAM1            ACAGAGTTTAACAATGCCTTTTCGTACCAGTGGTTCGCCACCCGTCAAGCGAATTTTCTTCACTCCCTGACTAACAAAAAATGCGGCTAAACGCTCAACT 10800 
GP59            .................................................................................................... 10800 

JAM1            TCTTCAAGACTGAGTATTTGTTGCCGAGGAAGAAAAGTCATCTTCTCTGACATGCAGTAAACACAGCGGAAATCACATCGATCGGTTACTGATATCCGCA 10900 <= moaA
GP59            .................................................................................................... 10900 

JAM1            AATAATCAATCTGTCGTCCCAGCCCATCTACCAGTCCTGCATGCTTCTGAATGTCAGCCATGGTTAGTTTCTCGTTGTTATCTTGGTGAGAGGGGAGCTG 11000 
GP59            .................................................................................................... 11000 

JAM1            TTATTGTCTGCAGTAATCCCCCCATCGCTGCAGCGTCCAGCAGCCATATGTCAGAGATAGTCCTTATCTTTTTTTCAAACGCTTTCAGTAACAGTTGTAT 11100 <= HP
GP59            .................................................................................................... 11100 

JAM1            TTTGCAGGAGTTCCAGCTTTTGTTTTATCTCTGCTTCGAGGCTATCCGCTTGCTCTAGATTACAGTCTTCACCGTAACGCTTACGTAAACCGAAATCGCT 11200 445183
GP59            .................................................................................................... 11200 

JAM1            TAGCGTCAAATGTAAATCAGGTTCTACATCAATATTATCAAGACAGGCCAGCGTGCAATGCATTTTGCAACCATCGATGGCCACAATCCGTCGTCCGGAA 11300 
GP59            .................................................................................................... 11300 <= HP

JAM1            CGAGCAGTACGAACCATTTTTGGCACTTTACCGCCCACACCGGCAATACAGGACATTTCTGCAGCACCAGAACGATCCAGTCGTAGCGCTAGAGTATTCG 11400 445383
GP59            .................................................................................................... 11400 

JAM1            CCAACTGAGCTACATTTGAGCAGCCGGAACACGAGTACACAATAGGCAATTGGTTGGAAGACATGCAACCCTCCTGTTTACATTAAAAAATAAATACTTT 11500 
GP59            .................................................................................................... 11500 

JAM1            GGTCTGTTCGGCAAGTCAGCAAAATGACAGCTGTTTTTTTGTAACCAGCTTCGGGCTCAATCAATTATCCTAAGATAATTACACTTGTATCAGATCAAAA 11600 
GP59            .................................................................................................... 11600 

JAM1            GTAGTCTACTTATCTGTTTTTCAGTAAGATTTAACGGAGCAAAAAAGCTCTCACTTCAAAACCCAGTTTAAACAGCTTCCTTGCATTAATATCGTGTTGC 11700 
GP59            .................................................................................................... 11700 

JAM1            TTCTCGCGCTTGATTATCTGTTCGCACCTCCACCGTTGAAAAATTAAACTGGTCTGATAAGGCGAAAAAGGTGCGGTGTTGATATTCTAACGTAAACCTT 11800 
GP59            .................................................................................................... 11800 

JAM1            ATTAAGGCTGAGGCCCTGGAGAGAGGTTGAAAAGTCACCTCCGGGCGTGAGCAGAAGCTTTGCTCGTCATCTGCAAAGAACAATTACAAAACGACCGCGC 11900 
GP59            .................................................................................................... 11900 

JAM1            AAACAATCCATCCATGGTAATAAACGCTGTTGAGTGTAAATCCTAAAAAAGGAGAGATACGTTGATATATCTCACAATCGGTATTTCTCAATCACTGCTT 12000 fnr
GP59            .................................................................................................... 12000 

JAM1            CAGCTCTTTCCAGTCATAAGCGGCAGTGATGTATGTTCAACCTTTCCAAAGGCCAGAAAGCTTAGCGGGATACGGTTCTTTAGAGCCTTTCTTTTCGAAA 12100 
GP59            .................................................................................................... 12100 

JAM1            AACAATAACGATGACAAACAGTAATCCTACTATAGACGTTTCTTTCCATCATTGATCAGGATCAATAACCGGCCTGGTTCCCTACACTGTTGGCTGCTAA 12200 fnr
GP59            .................................................................................................... 12200 

JAM1            TTTTATGCGAAGAGGTGTAAGCTAAACCGGTTATAGTATTTTCCATGAGGAACCTCAGTGTTGAAACTGAAAATCCGCGAAAAAATCACTGGTCTGTTAG 12300 => narX
GP59            .................................................................................................... 12300 

JAM1            TGTTTTATTTTTTATGTGCCCTGATTGCCATAAGTTCTACCCTTTACGTTTCATGGAAACTTGAAGGTGGTGCTGCTGCGATCAATGACGCAGGCAGAGA 12400 446383
GP59            .................................................................................................... 12400 

JAM1            ACGGATGCGCTCATACCGTATCGCCTATCTATTGGGGCAACAGGTACATTATCCGTCTCCTGATCTGGAGCATGCTCTCACTCAAGAAATGGTCTTTTTT 12500 
GP59            .................................................................................................... 12500 

JAM1            GAAAACACATTGGTCGAATTGCAAAACGGAAACCCACAAAGACCACTATTTGTTCCAGAAGATGCCATTATAAAGGAGCAGATGAACCAGTTGCGTAGCA 12600 
GP59            .................................................................................................... 12600 

JAM1            GTTGGTACAATACGATGAAACCGGGCATCCAAAAGATTCTTGATACCCCGGCAACACTGCAGAAAGACACGCTTCTGACGAATTATCGCCCCATGATGGA 12700 
GP59            .................................................................................................... 12700 

JAM1            AAGTTTTGTCAAAGAGCTTGATGAACTGGTCTTTTTAATGGAAAAAAATTCTGCGCACTATACAACCATGCTACGTTACATACAGATTGCATTAATGGTT 12800 
GP59            .................................................................................................... 12800 

JAM1            GTCGCCTTACTGGGAACTATTTTTTTAGATTACATCTTTTCATTGCTACTGGTACGGCCGGTGCAAAGAATTAACCAGGGATTACAGAGCATGGGAAAGG 12900 
GP59            .................................................................................................... 12900 

JAM1            CCGACTTCGGTGTGCGTCTGCCAGCATCTACTAATGATGAATTAGGAGAAGTGGCTCAGGGCTTTAATCAGATGGCAGAAAAGCTTCAGAATCTTTATAC 13000 
GP59            .................................................................................................... 13000 

JAM1            CACTCTGGAGCAGCGGGTAGCTCAGAAAACAGACAGTATTAAAGTGAAGAATCGTGAACTTGGCGCACTGTATAAAGTGGCTGCTTTTCTAAGCTCAAGC 13100 447083
GP59            .................................................................................................... 13100 

JAM1            ACCTCTGCAGAGCCTCTGTGTGAAAGTGTATTAAAGCAGATGATGGATCTGACCGGAGCTCGTGGTGGTATCGTACGGCTAACGGATCCCAAGGGTGAAC 13200 447183
GP59            .................................................................................................... 13200 

JAM1            AGTTACACGTTGTAGCCGCCGAGGGAGTCAGTAAAGCATTTGTGGAAAATGAAAACTATCTATCGGTGGGCTCCTGTATTTGCGGCGAAGTTGCCCAAAA 13300 
GP59            .................................................................................................... 13300 

JAM1            TGGTATTGCCGTTAGTTCAGATTTGAAGACTTCCAGACAGCAATCTTGTAACAAGGAAAAATTCCGCGCCGTTTCGGCGATTCCAATTCGCTCGAATCAG 13400 
GP59            .................................................................................................... 13400 

JAM1            CGCCTTATTGGTTCCCTCAATTTATTATTCAATGTGGAACGGATTCTGCCGCCTGCAGAGATAAAGCTGCTTGAATCAGTAGGCTTACACCTTGGCGTTG 13500 
GP59            .................................................................................................... 13500 

JAM1            CTATTGAAAATCAGCGCTTGGTTGCTCGTGAACGAGAAATGGCCGTGTCAGAAGAACGTAATTTACTTGCACAGGAACTGCATGACAGCATTGCCCAGTC 13600 
GP59            .................................................................................................... 13600 

JAM1            GCTTGCTTTTTTGAACATACAAGTGCAGTTGTTACAAAATGATCTGAATAATGAAAATATTGCTGAAGCCCTACAAGTGGTTGACCAAATTCGAGAAGGT 13700 
GP59            .................................................................................................... 13700 

JAM1            GTGCAAGAAAGTTACGATGATGTACGGGAATTACTGGTACATTTCCGTACCCGGTTAAAACATGCTGATCTGGAAGGTGCTATCGCTAACGCATTGAATA 13800 
GP59            .................................................................................................... 13800 

JAM1            AATTCGAAGGACAAGTGGGAATCAGTACGTCTTTTGAATATTCTGGTCCCACCATGGACTTGCCTCCAGAACATGCGTTGCAAATTTTACATATCGTACA 13900 
GP59            .................................................................................................... 13900 

JAM1            TGAATCGCTTTCTAACATCCGTAAACATTCCACTGCCACACAGGTAAGTGTAGAACTTCGCACGGAAGGTGAAAGCCGTCTTACCATTAAAGACAATGGT 14000 
GP59            .................................................................................................... 14000 

JAM1            AAGGGTTTTAATATCGATGAAAAAAATGATGATACCCATGTTGGTATCTATATTATGCGAGAACGTGCCCATCGTTTCGGTGGTGAACTCTCCATTTCTT 14100 => narX
GP59            .................................................................................................... 14100 

JAM1            CTGCAACCAATCAAGGAACCGTCGTTAGCCTCTCCTGGCAGCCTGTTTCGATAATCACGGAGCAACGTATTACAGCATGAAACCGATACGCATCCTTATT 14200 => narL
GP59            .................................................................................................... 14200 

JAM1            ATTGATGACCACTCCCTGTTTCGTAGTGGTATCAAGTTACTGTTACAACGTCAAAAGGGTTTTGAAGTTGTCGGGGAAGCAGGCAATGGTCTGGAAGGGG 14300 448283
GP59            .................................................................................................... 14300 

JAM1            TTAAATTGGCGAAGCGTCTGTCTCCGGATGTAGTTTTGCTTGACCTGCATATGCCGGGAATAGGAGGAGTGGAAACTATTCCTCTGATAAAAGAGGAGGC 14400 
GP59            .................................................................................................... 14400 

JAM1            CCCTGAAGCCCAGGTAGTCATGCTAACGGTTTCTGAAGATGCAGAAGACTTGTTGGATGCCCTGCATAATGGTGCCCGCGGTTATTTACTGAAGAATATC 14500 
GP59            .................................................................................................... 14500 

JAM1            GACACCGATTACCTGCTGGACTCAATCCGTCGCGCTAATAACGGTGATTCGGTAATGTCTGTTGAAATGGCCAATAGAATGGCAGATGCCATGCGTGCTT 14600 
GP59            .................................................................................................... 14600 

JAM1            TATCCGATGGAAGCAATAAAAAAATAATCATGGATACCAATAAGCTCTCACCCAGAGAGCGGGATGTGATTGTCATGTTGGCACAAGGTGCCAGCAATAA 14700 
GP59            .................................................................................................... 14700 

JAM1            AGAAATTGCTCGCAACCTGAGCTTAGCTGAGTCAACCGTTAAAATTCATGTACAAGGAATATTACGTAAGCTCAACCTAACCAGCAGGGTGCAAGCTGCC 14800 
GP59            .................................................................................................... 14800 => narL

JAM1            GTATATGCGGTAGAAAACGGTTTGGTGGATAAATCATAATCTGTTTTTCAATCCGATATATTAGTTCTATATGGGAACGAGAGGGCATTTTTATAAATCC 14900 
GP59            .................................................................................................... 14900 

JAM1            GCAGATTACGCATATGTGCACGAATTAAGAATAAGTCTTTTAGTTACACTCTCCCGACACTAAAGCTGATGGACCGGGCTTTTGCAACCTAATGTTCGAG 15000
GP59            .................................................................................................... 15000 

JAM1            ATAGTTGAGAACTAAGCCGCTGTTGAACCAGACGATAGATGATTGGGAATGAATCGTTATTGCCGTGCCGATAATGGCATAGGTTTGTTCATCTCTTGGA 15100 449083
GP59            .................................................................................................... 15100 

JAM1            ATAATCTGCATCCAGCTGCTTGATCTGTGGATGTATGGATTCCTGCGGCCTCCCCTCCGCCTTCGCAATGAAGTCCTTTTGTACGTTATGAATGTTTGCG 15200 
GP59            .................................................................................................... 15200 

JAM1            GCGCCCCGCAGGGCGGTGATAACCCGTCGGAAAGTCTTTGGAGTCCGAGGGGCTCCATTATGTTCAAAGCGCTGTCTCACCAAGTGAAATAGCCGCTTGC 15300 
GP59            .................................................................................................... 15300 

JAM1            CTGTGGATGAATATTTTAGGACCTATCCGGCATATATTGTTTTTGTCTGCGTTACAGCCATGTTTTATTTTACCGATTTTCAGGCAGTCCTAGTTCAAAA 15400 
GP59            .................................................................................................... 15400 

JAM1            GAACTAGTTCTTTTAATTGCTAAACCTATGCTTCTGAGGAATATGCTCGATGCATGCATAAGCCTAAGATTGCACCATGTTCTAATCGTGGTGGTCTGTG 15500 
GP59            .................................................................................................... 15500 

JAM1            ATGATGAATCAACAATACAAAGCATGGTCAGTTGTTATTGCAAGCACGCTTTCTTTTACAGTGTGCTTCATGATCTGGATGATGTTTGCTGTTATAGGGA 15600 => narK1
GP59            .................................................................................................... 15600 

JAM1            TCCCTATTAAAGAAACCCTCGGACTTAATGAAACCGAGTTTGGCATATTGATTGCCACGCCGGTATTAACCGGGTCATTAATGCGGCTACCACTTGGCAT 15700 
GP59            .................................................................................................... 15700 

JAM1            GCTCACGGACAAGTTTGGTGGACGCATTGTTTTTTTCCTATTGATGCTCTCCACGGTCATACCCATCTGGTTGATCTCCTACTGCGCACAGTTTTGGCAA 15800 
GP59            .................................................................................................... 15800 

JAM1            TATTTGATTGTCGGTCTCTTTGTTGGCATGGCTGGTGGATCATTTACGGTAGGGATTGCTTACTGTGCTCATTGGTTTCCAAAGAATAAACAAGGATTGG 15900 
GP59            .................................................................................................... 15900 

JAM1            CAATGGGGATTTTTGGCGCTGGTAATACGGGCGCTGCAGTCACCAAACTGGTTGCACCACTTATTGTCGTGGCATACGGCTGGATGATGGTGCCAAAGGT 16000 
GP59            .................................................................................................... 16000 

JAM1            TTACGCAATTCTCATGTTGGTAACGGCCATCCTATTCTGGATTTTTACCTTTTCTGACCCTGAACATAAGGTCGCCAAAGCAATCACTATTCGAGAACAG 16100 450083
GP59            .................................................................................................... 16100 

JAM1            TTAGCGGTACTCAAAGACCCTAGAGTCTGGAGATACAGTCAGTATTACTCAATCGTTTTTGGTGGCTATGTCGCCTTGGCATTGTGGATGACTAAATATT 16200 
GP59            .................................................................................................... 16200 

JAM1            ACATCACGGAATATGGTTTTGATTTAAAAACGGCTGCGTTGATGGCCGCCGCGTTCAGTATCCCGGGGGGGATTCTTAGAGCCTTGGGAGGGTATTTTTC 16300 
GP59            .................................................................................................... 16300 

JAM1            AGATAAATTTGGTGCGCATACTGTGACCTGGTGGGTATTGTGGGTTTCGCTGGTATGCCTGTTCTTTCTGTCCTATCCACAAACTGAAATCAGCATTCTT 16400 
GP59            .................................................................................................... 16400 

JAM1            ACGGTAGATGGTTCAAAAAGTTTCCATCTCGGACTGAACGTACTCTTCTTCACCATCATCATGTTCACTTTGGGCATTGCTTTCGCCATAGGCAAGGCAT 16500 
GP59            .................................................................................................... 16500 

JAM1            CGGTATTCAAGTACATCTCTGACGATTATCCAGGCAATATCGGTGTGGTATCAGGTGTTGTTGGTCTTATGGGCGGCATGGGAGGCTTCCTACTTCCCGT 16600 
GP59            .................................................................................................... 16600 

JAM1            CATGTTTGGCGCATTAGTCGACCTGACTGGTATCCGTTCATCCGTTTTTATGTTGATGTTCGGCATCATCTGGGTGTCATTGCTGTGGATGTACTGGACC 16700 
GP59            .................................................................................................... 16700 

JAM1            GAGGTTCGCCCCACCAAACTTGGCAGCAATCAGCTGCCAAGCCAACCATTAAAAAGTCAATATTAAGGAGCCCATAAAGTGACTATCAATATTAAAAAGT 16800 =>narK2
GP59            .................................................................................................... 16800 

JAM1            GGGACGTTGAGGACCCGGAATTCTGGGAATCAACGGGTAAACGCGTCGCCTACCGTAACTTGTGGATTTCAATACCCTGTCTGTTATGTGGTTTTGCGGT 16900 450883
GP59            .................................................................................................... 16900 

JAM1            ATGGCTGATGTGGGGCATGATCAGTGTTCAGATGTTGAACCTTGGTTTCCCGTTCAGCAAAGAAGAATTATTCTCGTTGACGGCCATTTCCGGCCTGGCG 17000 450983
GP59            .................................................................................................... 17000 

JAM1            GGGGCGACAATGCGCATTCCGTCTTCCTTTTTCATCCGTTTGGCCGGTGGACGGAATACGATTTTCCTGACAACAGCAATGCTGATTACCCCAGCCGTTG 17100 
GP59            .................................................................................................... 17100 

JAM1            GTACAGGGATTGCCCTGCAGCATCCAGAATGGCCGCTGTGGGTGTTTCAGTTGCTGGCATTATGGTCAGGTGTCGGCGGTGGCAACTTTGCCAGTTCAAT 17200 
GP59            .................................................A.........C.G.....T........T..C.................... 17200 

JAM1            GAGTAACATCAGTACCTTCTTTCCTAAGCGTTTACAGGGAACTGCCCTGGGATTAAATGCTGGGCTCGGTAACTTTGGTGTAACAACCATGCAAATCCTG 17300 
GP59            .............................................T..............C.................C..................... 17300 

JAM1            ATCCCCCTGGTGATGACTGCCGGCGTTTTTGGGGCTATGGGTGGTGATCCGGTGGCGCTGGTCAAAGACAGTGGCTGGATTCTGGGCAAGATTGAAGCGG 17400 
GP59            .........................................................T................T......................... 17400 

JAM1            GCACCCCAACGTTTATTCAAAATGCTGGTTATGTGTGGCTTTTATTGCTGATCCCACTGGCAATTGCTAGCTGGTTTGGAATGAATAATCTGCTTTCAAT 17500 
GP59            ...................................................................C................................ 17500 

JAM1            TTCCTCTGATATTGGTGGTACGCTAGTTGCTTTTATCAAAATCACCTGGCTTTATACTCTGACCTTTATACCGACTATTGTCGGTCTGTACTTCTATCTG 17600 
GP59            ........................G................................G.....................A.................... 17600 

JAM1            CCTGCACCGACGGGACTGGGTATTTTGAATATGTGGGTTGCTTTGCCATTGATCATGGTGAGTACGCTGCTGATGATGAAGCTGGCTGCCTTTGGCACCA 17700 
GP59            ...........A........................................................................................ 17700 

JAM1            TGAAGGAAAACATCCAAAAACAGTTTGCGATCTTCAGTAATAAACATACATGGTCGATGACGGTTCTTTATGTACTGACTTTTGGTTCTTTCATCGGCTT 17800 
GP59            .................................................................................................... 17800 

JAM1            TTCGATGGCATTGCCATTGTCGATCACCATCATCTTTGGTGAAAAGCATATCTATGATGTTGCCACCCAAACGTGGCTTCACGTCAGGAATCCGAATGCA 17900 
GP59            .................................................................................................... 17900 

JAM1            CCCTCTGCACTTACTTATGCCTGGATCGGGCCTTTTGTCGGAGCGTTGATTCGCCCGGTTGGTGGCTGGATCTCCGATAAGGTAGGCGGTTCCATTGTTA 18000 451983
GP59            .................................................................................................... 18000 

JAM1            CTCAGATGATCTCTGCAATCATGGTGCTGGCCTCAGCCTACGCAGGATTTGTAATGATGCAGGCGTATCAGTCGGCCACCCCTGAAATTTACTTTACTCA 18100 
GP59            .................................................................................................... 18100 

JAM1            ATTCCTGGTGGTGTTTGTCATCTTGTTCGCTGCAAGTGGCATAGGTAACGGTTCAACTTTCCGTAGTGTAGGGATGGTTTTTGATCGGGTACAAGCAGGA 18200 
GP59            .................................................................................................... 18200 

JAM1            CCAGTACTGGGTTGGACATCAGCAGTTGCTGCTTATGGTGCCTTTATCGCCCCGGCAATCATTAATCAACAGATCCAGGCCGGCACGCCACAAATTGCCA 18300 
GP59            .................................................................................................... 18300 

JAM1            TGTATGGTTTCGCGGGATTCTATGCATTGTGTTTGGTATTGAACTGGTGGTTCTATCTGCGCCCCGGCGCTGAAATAAAGAACCCTTAAAAGCCACCCTG 18400 
GP59            .................................................................................................... 18400 

JAM1            AAATAACGATTTTAATTGGAGCAAACAATATGAGTCATTTTCTAGACCGCTTGAATTTCTTCGACAAGGTCAAAGAGACCTTCTCCAACGGCCATGGCAT 18500 => narG1
GP59            .................................................................................................... 18500 

JAM1            CGTCACTAACGAAGATCGCCAATGGGAAAGCGCTTATCGCCATCGTTGGCAACATGACAAGATCGTGCGTTCTACGCACGGTGTTAACTGTACAGGAGGC 18600 
GP59            .................................................................................................... 18600 

JAM1            TGTAGCTGGAAGATTTTCGTTAAAAACGGCTTGGTGTCATTCGAAATGCAGCAGACCGATTATCCACGGACCCGAGAAGATCTACCTGATCATGAACCAC 18700 
GP59            .................................................................................................... 18700 

JAM1            GTGGTTGCCAACGTGGCGCTTCCTTCTCGTGGTATCTCTATAGCCCACATCGTATCAAGCATCCGATGATTCGTGGCCGTTTACTTGATTTGTATCGGGC 18800 452783
GP59            .................................................................................................... 18800 

JAM1            TGAACGTAAAACAGGTAAAGATCCGGTAGAAGCCTGGGCTGCGATTCAAGCTGATGAACAGAAACGCAAGCAATATACTGCGGTGCGTGGGTTAGGCGGT 18900  => narG1
GP59            .................................................................................................... 18900 

JAM1            TTTGTCCGTACTGATTGGGATGAAATCACAGAAATCGTCGCGGCTGCTAACGTCTACACCATCAAGAAATGGGGACCAGACCGTATTTACGGTTTTTCTC 19000 452983
GP59            .................................................................................................... 19000 

JAM1            CTATCCCTGCCATGTCGATGATTTCTTATGCTGCCGGCGCACGTTACCTGTCAATGATTGGTGCCGCATGTGGCTCATTTTATGACTGGTATTGCGATTT 19100 
GP59            .................................................................................................... 19100 

JAM1            GCCTGCTGCTTCTCCACAAACGTGGGGTGAACAAACTGACGTACCGGAATCGGCTGATTGGTATAACTCAACTTATATCATCATCACCGGTGCCAACTTA 19200 
GP59            .................................................................................................... 19200 

JAM1            CCAATGACCCGTACACCGGATGCGCATTTTGCTTCTGAAGTTCGTTATAAAGGGGCCAAAATTGTAGCCATGGCACCTGACTATGCCGAATTCTGTAAGT 19300 
GP59            .................................................................................................... 19300 

JAM1            TCTCAGACTTGTGGATGCCGATCCGTCAGGGTACTGATTCCGCGGCATTTCTGGCAATGGGCCATGTGGCGTTAAAAGAGTACTACATCGATAAGCAGGA 19400 
GP59            .................................................................................................... 19400 

JAM1            TCCTTATTTCCAAGAATACGCTCAGAAATATACCGATCTGCCAATGCAAGTCATGCTTCGCAAGCACGGCGATGCGTATGTTTCAGATCGCTTCCTGCGT 19500 
GP59            .................................................................................................... 19500 

JAM1            GCCTCCGATTTCGATAATGCTTTAGGAGAGACAAATAATCCGGATTGGAAAACCATCGTTTATGATGAAGTCAAAAAAGCGTTTGTTGCTCCTAACGGTT 19600 
GP59            .................................................................................................... 19600 

JAM1            CTATCGGTTTCCGCTGGGGTGAGGACGGTAAATGGAACCTGTTAGCGAAGAATGCCGCAACTCAGGAAGACATTGTTGCCGAACTGAGCTGCATCGATAA 19700 
GP59            .................................................................................................... 19700 

JAM1            CTTGGATAGTGTGGTTTCGGTTGGCTTTCCGCATTTCAACCTGGGAGAAGATGAACTGCTGTTTCGCAATGTGCCAGTACGCAAGGTTAAGCTTGCCAAC 19800 
GP59            .................................................................................................... 19800 

JAM1            GGCGAGGAAGCATTAGTTACTTCTGTTTTCGATATGCAGGTGGCGCAGTACGGTATTGACCGGAAACTGGGTGGCGGTAATGTTGCTCAATCTTATTCTG 19900 
GP59            .................................................................................................... 19900  => narG1

JAM1            ACGCTAATGTAGCCTATACCCCGGCATGGGCTGAAAAGGTAACTGGTGTCAAAGCGGCTGATATCGAGCGTACAGGGCGTGAATTTGCCTATAACGCTTC 20000 453983
GP59            .................................................................................................... 20000 

JAM1            CAAAACCAAAGGTAAATCCATGGTTATCATGGGAGCTGCCATTAATCACTGGTACCACAATGACCTGGCTTATCGTTCGATCATGAATCTGCTGCACATG 20100 
GP59            .................................................................................................... 20100 

JAM1            TGTGGTTGTGTCGGTCAGTCAGGTGGTGGCTGGGCTCACTACGTAGGTCAGGAAAAACTGCGTCCGCAAGCTGGTTGGGCACCGATCGCCTTCGGAACGG 20200 
GP59            .................................................................................................... 20200 

JAM1            ATTGGAGCCGTCCTCCACGTCATATGAATTCTACCTCGTACTGGTATTTCCATACTGATCAATGGCGTTATGAAACGGTAAAAGCTGATGACTTGCTGTC 20300 
GP59            .................................................................................................... 20300 

JAM1            TCCTGCTGGTAAAGGTAAGTACAAAGGTTATTCATTGGCCGACTATAACGTTGCCGCCTCACGTATGGGCTGGTTGCCAACTGCACCACACTGGAACGCG 20400 
GP59            .................................................................................................... 20400 

JAM1            AATCCTCTTAAGTTAGTTGCAGATGCTGAAAAAGCCGGTGCAAAAGATGAAGCTGGAATCACTCAGCATATCGTTGACAGGCTTAAAACAGGTGAGCTGG 20500 
GP59            .................................................................................................... 20500 

JAM1            ATGTTTCGTTTGCCGATGTGGACAATCCGGTCAACTGGCCTCGTAACTTGATTGTCTGGCGTGGCAACCTTATCGGATCTTCCGCCAAAGGGCATGAGTA 20600 
GP59            .................................................................................................... 20600 

JAM1            TTTTCTTAAACATTTGCTTGGCGCACAGAATGGTGTGTTGCAGGAATCCGGTGCCGGTCGTGATAACAAAGAAGTGAAATGGCATGAAGAGGCTCCTACC 20700 454683
GP59            .................................................................................................... 20700 

JAM1            GGTAAGCTGGATTTGATGGTGGACATAAACTTCCGCCTGAACTCTACCGGTGCGCATTCCGATATTATTCTGCCGACAGCGACCTGGTATGAAAAGAATG 20800  => narG1
GP59            .................................................................................................... 20800 

JAM1            ACCTCAACACGACGGACATGCATCCGTTTATTCACCCACTGTCAGAAGCGGTAAGTCCGGGCTGGCAATCAAAATCTGACTGGCAGATTTTTAAATCTAT 20900 454883
GP59            .................................................................................................... 20900 

JAM1            CGCCAAAACGTTCTCAGCACTGGCAGAAAAGCATTTGGGAACGGTCAAGGATATTGTCGCTCTGCCAATGCAACATGACTCCGCTGCTGAAATGGCTCAA 21000 
GP59            .................................................................................................... 21000 

JAM1            CCATTCGGTTATGTATCTGACTGGAAGAAAGAGGGACTGGAGCCTATCCCAGGTAAAACCATGCCAATTCTTAAAGTGGTAGAGCGTGACTATGCCAATA 21100 
GP59            .................................................................................................... 21100 

JAM1            CCTACCGGAAGTATATTGCTCTTGGTCCGTTAATGACCAAACTTGGAAACAATGTTAAAGGGATTGACTGGAACACTGATCAGGAATACGAAGAGTTAAA 21200 
GP59            .................................................................................................... 21200 

JAM1            ACAATACAACAAAACGGTTAAAGAACCGGGTATTTCCTTTGGGATGCCATCACTGGAAGAGGATATCTATGTCTGCGATGCGGTAATGCGCATGGCACCA 21300 
GP59            .................................................................................................... 21300 

JAM1            GAAACAAACGGTGAAGTGGCACATAAATCCTGGTCTGCCTTGTCTGTTAAAACCGGTATTGACCATCACCATCTGTATGTTGGTCGTCATGAAGACAAGA 21400 
GP59            .................................................................................................... 21400 

JAM1            TTACCTTCAAAGATATTCAGTCTCAGCCGCGTAAGATTATTACGGCACCAACCTGGTCCGGCATTGAGTCAGAAACGGTATCTTACACCGCTGGTTACAC 21500 
GP59            .................................................................................................... 21500 

JAM1            CAATATTCATGAGCATATTCCGTTCCGGACCTTAACTGGCCGTGCTCATTTTTATCAGGATCATGAGTGGATGCTTGATTTCGGTGAAGGCTTCTGCGCT 21600 
GP59            .................................................................................................... 21600 

JAM1            TATCGTGGACCGTTAGATATGAAGGCTCAGGAGGTTCTCCCAGATGCGGTCAGAGCCAAGCCTCACCTGGTGCTCTCATGGATCACGCCACATTCCAAAT 21700 
GP59            .................................................................................................... 21700 

JAM1            GGGGGATTCACTCCACCTATCAGGATAATCTGCGCATGCTGACGTTATTCAGAGGCGGACCATACGTTTGGGTATCAGAGACTGATGCACAGTCAATCGG 21800 
GP59            .................................................................................................... 21800 

JAM1            GCTCGAAGACAATGACTGGGTTGAAGCGGTTAACGGCAACGGCGCCACGATGGCTCGTGTCGTAGTATCGCAGCGTGTCCCCCGTGGTATGGCGATGATG 21900 455883
GP59            .................................................................................................... 21900 

JAM1            TATCACGCTCAGGAAAAGAACGTCAACGTACCGGGATCATCTTCCACTGGTAAACGCGGCGGTATCCTGAACTCCGTCACCCGCGTCATTATTAAACCAA 22000 
GP59            .................................................................................................... 22000 

JAM1            CCAATATGATCGGTGGTTATGCACAGCTCTCGTACAGCTTTAATTATTACGGGACAGTGGGTTGCCAGCGTGATGAACAGGTGGTTCTGCATAAGATTGC 22100  => narG1
GP59            .................................................................................................... 22100 

JAM1            GGATAAAGATATTGACTGGTTAGAACGTCCGCTGACAGCGGAGCGGGAAGAACAACGTAACCCAATAGGGGTCGGTAAACGCTAAGCGTTCCGGTCAGAA 22200 
GP59            .................................................................................................... 22200 

JAM1            TTATTAGGAGAATCACATGAAAGTACGCGCACAATTCGCTTTTGTTTTTAACTTGGACAAATGCATTGGTTGCCATACCTGCTCAGTAACCTGCAAGAAT 22300 => narH
GP59            .................................................................................................... 22300 

JAM1            GTCTGGACCAATCGTAAAGGTGTTGAATATGCCTGGTTTAATAACGTTGAATCCAAACCAGGTATTGGTTATCCCAAGCAATGGGAAGATCAAGGAAAAT 22400 
GP59            .................................................................................................... 22400 

JAM1            GGAAAGGTGGCTGGGAGCTTAAAAAAGGCAAGCTGGAGCTTAAATCTGGTACCCGCGCATCCAAGCTTAAGAATATTTTTGCTAACCCTGATTTGCCCGA 22500 
GP59            .................................................................................................... 22500 

JAM1            AATTGATGACTACTATGAGCCATTCAGTTTTGACTATGCACATCTGAAAAATGCTCCCCTGTCTGAGGCTGCGCCGACCGCACGACCTATCTCACAGATA 22600 456583
GP59            .................................................................................................... 22600 

JAM1            ACGGGCAAGTCGATGGAAAAAATCACTTGGGGACCAAACTGGGAAGACGATCTGGCCGGCGAGTATGCCAAGCGCAGCGAAGATCAGAACATGAATAACA 22700 456683
GP59            .................................................................................................... 22700 

JAM1            TCCAAAAAGAGATGTATGGACAGTTTGAAAATACGTTCCACATGTATCTGCCACGTATTTGTAATCACTGTCTTAATCCGGCCTGCGTTGCCTCATGCCC 22800 
GP59            .................................................................................................... 22800 

JAM1            TTCCGGTTCGATGTACAAACGGGAAGAGGATGGCATCGTTCTGGTAGATCAGGACAAATGTCGCGGATGGCGGATGTGTGTCAGTTCGTGTCCGTATAAA 22900 
GP59            .................................................................................................... 22900 

JAM1            AAAGTGTTTTACAACTGGGAATCAGGAAAAGCGGAAAAATGTGTCGGTTGTTACCCACGCGTAGAATCTGGAATGCCGACAATTTGTTCCGAATCCTGTG 23000 
GP59            .................................................................................................... 23000 

JAM1            TCGGTCGTATCCGTTACAACGGCATCATGCTCTACGATGCTGACCGCATTGAAGAGTTGGCAAGCGTAACGGATGAAGAAGATTTGTATGAAGCACAATG 23100 
GP59            .................................................................................................... 23100 

JAM1            TCGCATCTTCCTCGATCCGAACGACCCAGAAGTCATTGCAGCAGCACGTGCAGAAGGCATTAACGAGGATTGGATAGAAGCTGCACGTAAATCACCAATC 23200 
GP59            .................................................................................................... 23200 

JAM1            TGGAAAATGGCCATGGATTGGAAGATCGCTTTCCCTATCCATCCGGAATTCCGTACGCTGCCGATGGTTTGGTATGTACCACCATTGTCACCAGTACAGT 23300 
GP59            .................................................................................................... 23300 

JAM1            CACAGCTTGATCAGGGAAATCTGCCTGTCGGGCCTGACGGTGCCATTCCACTAGCAGGTTCTATGCGCTTGCCAGTTCAATATCTGGCTAATCTGCTCAC 23400 
GP59            .................................................................................................... 23400 

JAM1            CGCAGGTAAAACAGAGCCAGTCGAAAGTTCGCTCAATCGTCTTATTGCGATGCGTCGTTATCATCGTTCAGTTCATGTGGAAGGTGAAGCAGATACCCGG 23500 
GP59            .................................................................................................... 23500 

JAM1            GCGTTGGAAGCGGCTGGTATTACCGAAGAAACGGCGAAAGAAATGTACCGTTATCTGGCGATTGCCAATTACGAAGACCGTTTCGTTATTCCTACTGGCC 23600 
GP59            .................................................................................................... 23600 

JAM1            ATACAGAAGAAACGCTGGATGATGCTTTTGGCTTCCAGGGACAGAATGGCTTTACTTTCGGCAATGATACTTCCGCCGGTGTCAGCAAGATCACGTTGTT 23700 => narH
GP59            .................................................................................................... 23700 

JAM1            TCCACGTCGTCGTAAAGAAACAGTGGAATCCAAAGAGCTGGCACCTCCAGCGGATGAGTAACAGGAGAATATGATGCAAATATATAAATTACTGTCGGTA 23800 => narJ
GP59            .................................................................................................... 23800 

JAM1            CTGCTGGAATATCCAAGCCAGGAATTGATCGATAACTTACCGGAAATAAAACAACGACTGGAGCAGAGTGACGATCTCGAAGATGATGAACTTGAGAAAC 23900 457883  fnr
GP59            .................................................................................................... 23900 

JAM1            TGCACGGGTTTAATGACTATCTTGCCAGTAAGTCATTGACTGAACTGCAAGAAGATTACGTGCAAACATTTGATATGACGGCAGAACATAGTCTTCATCT 24000 
GP59            .................................................................................................... 24000 

JAM1            ATCACATCATCTATTTGGCGATGACAAAAACCGTGGGCCGGCACTGATCGACTTGGGTGAGTTGTACAAAGATTATGGCGTGGAAGTTGTGACCAATGAA 24100 
GP59            .................................................................................................... 24100 

JAM1            TTGCCTGATTATCTGCCACTCACTCTGGAGTTTGCTGCCCAACTGGAAGATAACGAAGCGATGGTCTTCCTGTCGGATGCTAAAAAAATACTGACGATCT 24200 
GP59            .................................................................................................... 24200 

JAM1            TGACCGAAAATTTGGAAAAAGCCAGTAGCCCATACGCGGCACTACTATCCATAGTGGGAAGTCGCGCCACTTTAACGCGCTTAACTGCCCAAGGAGTATC 24300 
GP59            .................................................................................................... 24300 

JAM1            ATAATGAGTCTGCACAATTTTCTCTATGGAGTTTATCCGTATATTGCTTTGAGTTTGTTTTTGTTCGGTAGTCTTATGCGGTTTGACCGTGAGCAATACA 24400 => narI
GP59            .................................................................................................... 24400 

JAM1            CATGGAAAAGTGATTCCAGCCAATTGTTATCAAAGAAAAATCTGCGACTAGGCAGTAATTTATTCCATATCGGCATTATTGCCATTTTCTTTGGACATGC 24500 458483
GP59            .................................................................................................... 24500 

JAM1            GGTCGGGTTGTTAACACCACATAGTGTTTTCACGGCAATCGGAGTCTCCGATATGGCCCATCAGATGATTGCAATCTGGGCCGGATCAATTTTTGGGACT 24600 458583
GP59            .................................................................................................... 24600 

JAM1            TTATGTTTGATTGGTGGTGCAATGCTGTGGATCCGCCGTATGTTTAATCCACGTGTCTCAGCTGCAAGCCGTGGCTCAGATAAGTTTGTGCTGAGCTGGA 24700 
GP59            .................................................................................................... 24700 

JAM1            TCATGGTTACCCTGATCATTGGAATGTCAACTATTCCAGTATCGATTGGTCATGCTAATGCTGGCGATCCGGGCGTGATGATTGCTCTGGCTGAATGGGT 24800 
GP59            .................................................................................................... 24800 

JAM1            GCAAAGTATCGTTTATTTGCGACCTGAACCAGCTTTACTGATGGGTGTGGACACCATTTTCAAAGTACACCTGTTTTTCGGTATGACAGTATTTCTGGTG 24900 
GP59            .................................................................................................... 24900 

JAM1            TTCCCGTTTACCCGTTTGGTACATATCTGGAGTGCGCCAATTGGTTACTTAGCAAGACCGTATCAAATTGTTCGCAGTAAACGTAAGTTTAAGTCTTGAT 25000 => narI
GP59            .................................................................................................... 25000 

JAM1            ATAAAAGGCAGTCATATGCGAGATGTTGGAAAGTTTCTTCAGGGATTTAGACGGTTCCAGCAACATTATCTGGGAGAGCAGCACGCACTGTTTGATCAAC 25100 => Carbonic anhydrase
GP59            .................................................................................................... 25100 

JAM1            TGCTTACAGAAGGTCAACGTCCGCGAGCTCTGATGATTGCTTGTTGTGATTCTCGCTGTGATCCGGCATTGCTTACGGATTGTGAGCCTGGTGATATGTT 25200 
GP59            .................................................................................................... 25200 

JAM1            TGTGGTACGGAATGTCGCCAATCTGGTGCCTCCCTATGATCAGGCCAGATTGTTTGCCGCTACAAGTTCTGCCATTGCTTTTGCCGTTAGCAGTCTTGAA 25300 fnr
GP59            .................................................................................................... 25300 

JAM1            GTAGAGCATGTCATCATTATGGGACATGCGCATTGTGGTGGTATTCAGGCATTAATGACACACAAAACGCCTGAGAATGATGAATCGAAATTGATTTCAC 25400 
GP59            .................................................................................................... 25400 

JAM1            AGTGGATTGGAATTGCCGAATCAGCTCGTGAGCAGGTGTTGCAACAATTGCCCAATAAAACGCCTGAGGTTCAGGCGCATGCCTGCGAGCAGGCCTCAAT 25500 
GP59            .................................................................................................... 25500 

JAM1            ACTGATCTCGCTGGAAAATCTCCGGTCGTACCCATGGATTAGCAAACGTGTAGAGCTGGGCAAACTGGCTTTACACGGTTGGTATTTCGATATGGATAAA 25600 459583
GP59            .................................................................................................... 25600 

JAM1            GGTGAATTACTTCAGTATCAGCAAGAGAGTGGCAAGTTTGAAGTGCTTGTGGCAAATGGCGACAAAAGACCGATAAGCCCTTTTTCTAGCTCCAAAAGAT 25700 
GP59            .................................................................................................... 25700 

JAM1            AGCCCTTTATAGCAGCAAATACATGGGGGCAGGCCTTAACGTTGAAAATTCTTAAAGTAAGGATATCATCATGAACATACACTCCCGCCCATCGGCAATA 25800 
GP59            .................................................................................................... 25800 

JAM1            ACAATGCTGGCAAGTCTAATCAGTGTTTTATTTGTACCGCAGGCACAGGCAGAACAAGACAATCTTCTTGAAGCGATAACGGAAGGTAACGTCAGCTTTT 25900 => HP
GP59            .................................................................................................... 25900 

JAM1            CAGCACGTATGCGTTATGAGTCAGTAGAGCAGGACAATGCTTTGAAAGATGCCGATGCGCTGACGTTACGCACTACCTTGGGTTATAAAACAGGGGCTTT 26000 
GP59            .................................................................................................... 26000 

JAM1            CCACGGTTTCAGTGGCTTTATTGAGTTTGAGGACGTGTCTGAAATTGGTTCAGACAATTTTAACAGTACAACCAATGACGAAACAGAGTATTCGGTTGTG 26100 
GP59            .................................................................................................... 26100 

JAM1            GCCGATCCGGATAGTACCGAAGTTAATCAGGCTTATCTGTCATATAACGGTTTTGATACGGAAGTAAAAATTGGTCGCCAGGAAATCACCTATCGTGATG 26200 
GP59            .................................................................................................... 26200 

JAM1            CACCTTTTCATCGTTTTATTGGTAACGTGCTATGGCGTCAAAATCATCAATCATTTGATGCTTTGAGCTTCAGTAATACCTCGCTGCCTGATACCAAAAT 26300 
GP59            .................................................................................................... 26300 

JAM1            CAGCTACGCTTACCTCAATAAAGTCCATACCATCTTTGGTCATGACCGTGATGCTGCCGCAATCTTTGTTAAAGATGGCGATGTCGATATGAACAGTCAT 26400 460383
GP59            .................................................................................................... 26400 

JAM1            TTGTTTAATATTCAATACAGTGGTCTGCCGATCGGCAGCCTGGAAGGTTATAGCTATCTACTGGATTATTCTGATGCACCAGATGACAATAAGTTTTCAA 26500 460483
GP59            .................................................................................................... 26500 

JAM1            CCGAAACCTACGGGTTACGGTTAAGTGGTGTGCAGGCTGTTAATGAAACAACAAAACTGATTTATAGCGCTGAATACGCAACCCAAGACGATTTTGCTGA 26600 
GP59            .................................................................................................... 26600 

JAM1            TGGTGAAATGGATCGGCAAGATTACTATCTGTTTGAACTGGGCGGTAAATATAAGGGCTGGCTGGCCAAAGTGTCTTATGAAATGCTGGAAGGTGATGGT 26700 
GP59            .................................................................................................... 26700 

JAM1            ACAGACAGCTTTCAAACACGTCTGGGCACCAACCACGCTTTCCAGGGCTGGGCTGATCAATTTTTGGCTACGCCCGATGTAGGCCTGGAAGATCTATTCT 26800 
GP59            .................................................................................................... 26800 

JAM1            TTACGGTTACCGGCAGTGTTTTTGGTGCCAAATTGGTTGTGGTTTATCATGATTTTGAAACCGATAAAGGCAGTTTGGATGCCGGTAATGAACTCGACAT 26900 
GP59            .................................................................................................... 26900 

JAM1            TCTGTTAGAAAAAACCTTTAAAGAGCATTACACTTTAGGTGTGAAATATGCCGACTACAATGCCGATAAAGAATATGCATCATTGGTGGACACCGAAAAG 27000 => HP
GP59            .................................................................................................... 27000 

JAM1            TTCTGGGTTTATGGACAAGTTAAATTCTAATTTCTGTGCGGCGTAATTATAAAGTTGTAATGCATCTTGATTATAAAACGAGGGATTGTGGATCTTTTTA 27100 
GP59            .................................................................................................... 27100 

JAM1            GATCCGCCTTTGTTATGGATAAAAAGGCACCACAGAGGCAGGAGGGGGCCATTTGCGAGTTACCTCCGTGTATTTCGCATCATCGGGATTTATGATCAAA 27200 
GP59            .................................................................................................... 27200 

JAM1            ACGTTTATTGCCATGGCTGGGTGGTCGCTCCTGTGTGGGCTTGAGCCCACAGCCCAGCCTTACACATCGTCAGAAAAGTTAAACACTGGAACAGTGTATG 27300 
GP59            .................................................................................................... 27300 

JAM1            ACCTGACGCTCCTGTAGTTACTCAACGAAATCACCGAACAAGGAGCTTACACATCCAGTTCACATCTGGATCTAGAGTAACAACTTCCACTCACAGTACA 27400 
GP59            .................................................................................................... 27400 

JAM1            ACGCCTAAACCGGCTCTGGGTAGACGCAACACTCTTCAGTGACATTGTTTTAAAAAATGGGTGGATAGGTCAGATTTGCCGATCCTTGTATTAGGGTACG 27500 461483
GP59            .................................................................................................... 27500 

JAM1            TCTGCCCCAGATATTTTCAAGGTATCCGTCTTTTTGATGGCAAAAAAAGTCAACACTTTCTGTCGCTTTAAAGTCCTCCTATTTCAGTACATCAACCCCT 27600 461583
GP59            .................................................................................................... 27600 

JAM1            GAAAAATGAAACTATCAATAGCACACCACAAACAATCTTGATCCAGATCAATTTTTATTTGGCTTAAGGAAAATCCTGAGGAAGAGCCTTATGACAACAG 27700 fnr (coli)/anr 
GP59            .................................................................................................... 27700 

JAM1            AGTATGGTTAACTCCACTGGCTGAATCGTCAGGCAGTAATTACTTAAATGAGGAGATTTACTATGCAATGGAAAGATCCAGCATATTGTGATTTACGCTT 27800 => pqqA
GP59            .................................................................................................... 27800 

JAM1            AGGCTTTGAAGTTACAGCGTATGTTTTTGTTCGTTAATTAAACTTATAAACAGTGAGAGAGTGGAAACAATCACTCTCTCTCTGTTTATAACAAATCATA 27900 461883
GP59            .................................................................................................... 27900 

JAM1            TATTGAAATATATAAATTGGATTTGTTACATGGTTAAAAATATAACGCCGGAAAATATTGTTGAATTAGCACCACTCTACCTGTTTCGATGGGAAGAACC 28000 => pqqD
GP59            .................................................................................................... 28000 

JAM1            ACAGCAGGCCCATATCCTGCTCTATCCTGAGGGTGTGGTAAAGCTCAATGAAACGGGGGCGGCAATAATAAAGCATTGTGATGGCACCCATACGGTTAAA 28100 462083
GP59            .................................................................................................... 28100 

JAM1            GAAATCAGCACACTGTTAAGTGATCTCTATACCACTGATGTCAGTGAAAGTGTTCAAAAATTTTTGGAGGTGTCCTATGCCAAAGGATGGATCAGAATCA 28200 => pqqD/E
GP59            .................................................................................................... 28200 

JAM1            AGTCTTGATCATTCAGAATTAACGCTGGATGGTTTAGGGATACCGATGTGGCTCACGCTAGAGCTGACCTATCGCTGTCCTTTACATTGCCCTTGGTGCA 28300 => pqqE
GP59            .................................................................................................... 28300 

JAM1            GCAACCCTTTGAATTTCGATAAATTCAAAAATGAGCTTTCAACAGAAGAGTGGAAAAAAGTTCTACGTGACGGCCGAAAAATGGGGGCATTACAGCTGGG 28400 462383
GP59            .................................................................................................... 28400 

JAM1            ATTTACCGGTGGTGAGCCTATGCTGCGAGATGATATTGAAGAACTCGTTAGAGAAGCAGATGGGCTCGGGTATTACACTAACTTGATTACCTCAGGCGTT 28500 462483
GP59            .................................................................................................... 28500 

JAM1            GGTTTGACGCCCGAACGTTTGAAGGCATTGAAAGCCGGCGGACTCAAGCAGATACAGCTTAGCGTGCAATCCAGTGATCGCGAGTTATCACACCAATTAG 28600 
GP59            .................................................................................................... 28600 

JAM1            TTGGTGTTGATGTTTTCGATCAAAAAATGGCGGTAGCAGAAAATATTAAAGCCGAAGGGTTCCCTATGGTTTTAAATATCCCTATCTGTAAACAAAACAT 28700 
GP59            .................................................................................................... 28700 

JAM1            TGATCAAACACGCGCTATGCTGGAAATGGCCGAAAGGCTTGAGGTTGAATATATCGAATTTGCCAATATTCAATATTACAACTGGGCATTATTGAATCGC 28800 
GP59            .................................................................................................... 28800 

JAM1            GATGAGTTTCTGCCTTCTCTCGACCAGTTAAAAGCTGCCGAAGCTGTTGTTGCAGAATATCGCGAACGGCTTGGCAACAAAATGCAAATTTATTTTGTTA 28900 
GP59            .................................................................................................... 28900 

JAM1            TTCCCGATTACTTTGATGATCGTCCAAAAGCCTGTATGAATGGTTGGGGAAATATTCATTTGACCATCGGACCGGATGGTGCTGCTCTTCCCTGTCAGGA 29000 
GP59            .................................................................................................... 29000 

JAM1            AGCGCGCGTAATCAAAGGACTTGAATTCCCCAATGTCCGGGATGAGTCTTTGGATTGGATCTGGAATAACTCTCCAGTATTTAATGCTTATCGTGGCGAT 29100 
GP59            .................................................................................................... 29100 

JAM1            GACTGGATGAAAGATCCGTGTAAAAGCTGTGATGAAAAAGAACAGGATCATGGTGGTTGCCGATGCCAGGCTTTTCTGCTGACAGGTGATGCTGCTAACA 29200 
GP59            .................................................................................................... 29200 

JAM1            CAGATCCTGTGTGTTCTAAATCACCGATGCACGATGTTGTTGGATCTTGTGTAGCCAACTCCACCAATCCCCATAGAGTTGAAAAACCACTGGTTTTCCG 29300 
GP59            .................................................................................................... 29300 => pqqE

JAM1            TAAAAAGGGTGCAATCTCAACCGATTTTCTTCGATAAAAGCTTATGCCGGGCTCATAACTGATTGATGGTCGCATCATGTTGTTATGGGCCTATCACGAT 29400 
GP59            .................................................................................................... 29400 

JAM1            ACTTTTTTGTTTACCGAAACGATTACGGTAATAATTTTTGACGCATGGAGTTCTGATGACACTTACTGCATCTATAGATCTTTTCGCCAGATGGCTACAT 29500 => HP 
GP59            .................................................................................................... 29500 

JAM1            CTGATGGGCATACTTGTATGGATGGGCCATAATTATGCCAATGTGATCCAAAATCCTCGGTTTAAACCGGCACAACCCAGCGACCCTGGATCAATGCTGG 29600
GP59            .................................................................................................... 29600 

JAM1            CCGCAATGCAACGCGAACACGGCACTTTCCGTTATGCCTCACTGGTAGTCTTGGCTACAGGCGTTTATATGCTGTGGTATAGAGGCTTATTAATGGATGC 29700 463683
GP59            .................................................................................................... 29700 

JAM1            TTTAACGCTATCAGATTCAGCTTTATACATTGGCATAGGGGTCTGGTTGGGAATTATCATGGTTCTCAATTTATGGTTTGTTCTTTGGCCACATCAGAAA 29800 
GP59            .................................................................................................... 29800 

JAM1            AAAGTTCTTGGTTTTGTTCCTGCTGAGGATAATGAGCGTATTCGTTGTTCTCGCATCACTTTTCTATCCTCTAGAACAAACTCTGTCCTTTCAGTGTTCA 29900 
GP59            .................................................................................................... 29900 

JAM1            CCTTGTTTTTCATGATTGCCGGTGCTCACGGATTAATTTTGTTTTCCTAAGCAGAGCTATCTCACTATGTATAATTTTTCTGCTGGCCCGGCTCAGCTTC 30000 => Ph-Ser Trfase
GP59            .................................................................................................... 30000 

JAM1            CCAAAGAAATATTAAATAAGGCTAAAGAAGATCTGCTGGATTGGCATGGCTCTGGTATGTCAGTAATGGAGATGCCCTTTACCTCGGATGAATATAAAGC 30100 
GP59            .................................................................................................... 30100 

JAM1            TATCAGTGAGAGAGCCCACAACGATCTGCGTAAATTGATAAACCTGCCGGATGATTATCATATTTTGTTTCTACAAGGTGGGGCTTATGCCCATTTTGCC 30200 464183
GP59            .................................................................................................... 30200 

JAM1            CTTGTCGCTATGAATTTATTGGGTCAGAAACAAACCGCTGATTACATACAGACGGGTCATTGGTCGACACGAGCGTTGAATGAAGCCAGACGGTATGGCG 30300 464283
GP59            .................................................................................................... 30300 

JAM1            ATATCAATATAGCCGCCAGTTCTGTGTCTTCAGGTTTTAACCGCATTCCTCCCTATGAGCAGTGGCAACTGAACCCGGAGGCGGCATATTGTCATATCAC 30400 
GP59            .................................................................................................... 30400 

JAM1            CACCAATGAGACCGCTAACGGGGTTCAGTTTCATGATATTCCCAAGCTACCCATCCCTCTGGTGGCAGATGTCACTTCTGATTTTTTGACCCGCCAATTG 30500 
GP59            .................................................................................................... 30500 

JAM1            GACGTTTCACAATTTGGTCTTATCTATGCCAGTGCTCAGAAAAATATTGGACCAACGGGATTAACGATCGTCATTATCCACAACACGCTATTGAATCAAG 30600 
GP59            .................................................................................................... 30600 

JAM1            CAATGGAAATGGTGCCGACAGTCTTTAACTATGGCCGTCAGGCAAGCAACAACGGGCGAGTTAATACTCCACCGACTTACAGCGTCTATATCGCCGGATT 30700 
GP59            .................................................................................................... 30700 

JAM1            GATGTTCTCATGGCTGCTAGAGCAAGGTGGAATAAAAGCAATAGAACAAGCCAATCAGCATAAAGCTCAGCGACTTTACGAATTCATCGACAATAACAGT 30800 
GP59            .................................................................................................... 30800 

JAM1            TTTTATCAATGCTCTGTTGATCCATCTGCACGCTCTATCGTCAATGTTTGTTTTGAATTAACCGATATGAGCCGGACAGAAGACTTTCTCAGTGAGGCGA 30900 
GP59            .................................................................................................... 30900 

JAM1            GTAAAAAAGGCTTTGTTAACCTAAAAGGGCATGGCATTCGAGGCGGAGTTCGGGTCAGCCTTTATAATGCAATGCCCATTGAGGGTGTTGATGCTTTGGT 31000 
GP59            .................................................................................................... 31000 => Ph-Ser Trfase

JAM1            TGCTTTTATGAAAACTTATTCGGCCAGCCAATCCCAAACACGGTTGAAAAAATCTTTCCATGGATAACAGATCATAGATCAGATGGCGGCGAACTATACA 31100
GP59            .................................................................................................... 31100 

JAM1            AGTTATCAATACAAACGAGCCTATTTAATCGCACATATACTTAGCCGGATAAACAGGTTTTTTCCACATTCACCGCTATCGTCCGCAACTGTAGAAATAA 31200 => DiOHorottate deh
GP59            .................................................................................................... 31200    

JAM1            TGGGGATACGTTTTCCTAATTCGGTGGGACTTGCCGCTGGCTTTGACCGGTATGCCAGATTTTTGCCTTATACTCAACGGGTAGGCTTTGGCTTTATTGA 31300 465283
GP59            .................................................................................................... 31300 

JAM1            AATTGGCACTATTAATGTAAATGCGTCAAAAACGCCTGACAGTGCCGTCCTGGCAACATTAGATAACCTGAAAAAAGCTACGAAGTACTCCAAAAACAGT 31400 
GP59            .................................................................................................... 31400 

JAM1            GTGCAACGCCGGGGAATCAGTCTGGGCAGTCTTCACGATGGATTAACGAAACAAACGGAAGCCGACTTTGCAAACGGCATGATGTTATTTTGGCCGTACG 31500 
GP59            .................................................................................................... 31500 

JAM1            CGGATTACCTTGTCATTAATTTGAGCCGACCGAATAGCCCCACACGAAATCCTTCTCTTGAACCCAAAGCGTTAACGTCATTTTTAACTAATATCAAGCG 31600 
GP59            .................................................................................................... 31600 

JAM1            CGATCACAGCCAATTTGCTTCGGAATTTCGCCGTTACGTGCCGATCGTGGCAAAAGTTGCTATCGACCACGAGTGTAATGAGCATATTCCTGAAATCCTG 31700 
GP59            .................................................................................................... 31700 

JAM1            TTATCGTTAAAAGCGCAGCAGTTTGATGGTGCAGTAATTGCTTTTGAGAATTGGCCAGATATCAGTCGGGTAGCAGACTATGTGAGTCAATTAAAATCGA 31800 
GP59            .................................................................................................... 31800 

JAM1            CGAATGAAGATTTTCCATTAATTGTCGTTGGAGGCATACGATCTTGCGATGATATTGAGCAATTGATGGCAGCTGGCGCATCCCTGGTCCAGGTTTTTAG 31900  => DiOHorottate deh fnr
GP59            .................................................................................................... 31900 

JAM1            TAGCTTGATAACGCAAGGTCCATTACGAATGCAAAAAATGATTTCAAATTATTGAACAAAACCATGAATAACCTTAGGGCTTGTTGATCTTTGATGGTTA 32000 fnr
GP59            .................................................................................................... 32000 

JAM1            TTTTTTGAGCAGCATGGTGACTCGGTATAATGCGTTTCACAACAAACAAAAATTACACCAAATCACCATGCCAAGACTTATGTTAACCGACCAACGGTGG 32100 => Trpase 
GP59            .................................................................................................... 32100 

JAM1            CAAACGCTATCCAGACTGATGTATCAAAGTGGCCGTATCTACAACAAACCCGAGCACAGAATGACGCTCGAAGGGATCTTGTACCGAATGCGAACCGGCT 32200 
GP59            .................................................................................................... 32200 

JAM1            GCCCTTGGCGGGATTTACCCCGAGAGTTTGGGCAATGGAGTACCGTTTTCCGACGATTTAACTTATGGTCGAAGAAAGGTCTATTGGAAAGGCTATTTAA 32300 466283
GP59            .................................................................................................... 32300 

JAM1            AGCATTATCGCGCAGCACGGACATGGAATGGTTGTTCATTGATGGCAGTATCGTCAAAGCCCACCAACATAGCTCTGGCGCAGCCTCTCCCAAAGGTGAA 32400 
GP59            .................................................................................................... 32400 

JAM1            GCGATAGGTCAAAGCCGGGGCGACCATTCCACCAAAATCCATTTGGCGGTGGGGAGTTATGGTCTGCCAGTCCATGTCGAGTTATCAGGCGGTCAGGTCA 32500 
GP59            .................................................................................................... 32500 

JAM1            ATGATATCGTCCATGCTCAAAGCCTGATGGATGCCGTTTGCGCCGCTGACAAGGTGATTGCTGACAAAGGCTATGATAGTGAAGTCTTCAGGAATGAAAT 32600 
GP59            .................................................................................................... 32600 

JAM1            CGAAAGGCAAGGCGTAAAACCAGTCATTCCTCGACGTAAACATAGCCGTGTTGGCAATGAAAACATGGACTGGAGTTTGTATCGGTATCGACATCTGGTT 32700 
GP59            .................................................................................................... 32700 

JAM1            GAGAATGCCTTTGCCAGAATCAAGCATTTCCGGGCGATTGCAACGCGTTACGAGAAGCTCGAACGGAATTATTACAGCATGCTCATGCTGGCGTTTACTA 32800 
GP59            .................................................................................................... 32800 => Trpase

JAM1            TGATGTGGCTGCCAATGTGGGTTGATTAATTTTTGTACAGCAAAGATCAATAGCGGCTAGTAAAATAATCCTCCATGGATTCCTGACTTTTAACGCTGTG 32900 
GP59            .................................................................................................... 32900 

JAM1            TTCACAAAATGTTCAGGTATCAAAATTTATTCAACCAATCGCTTTACTAACCGTATGTTTATTGCAGCTGGATTTAGCGAATTGATATAAGGAATACACT 33000 
GP59            .................................................................................................... 33000 

JAM1            TTACTTTGATTTTTATCTTCATCAAACAATCAGGAGGGTAAAAGTGTTTTTTTGTGTAATTTTGTCGAAGAAAACGAGTAGAATAGCGCTATGCTGAAAA 33100 => nosR fnr
GP59            .................................................................................................... 33100 

JAM1            AGATTTTTTTATGGTCGAATCGTAACTTTTTGCAACGGGTTCTGTTGTTATTACTGATCTTCCCCAGTTTTGCTTATTCGATGGTCCCGGTCAATTTTGA 33200 
GP59            .................................................................................................... 33200 

JAM1            TGAGCAGACAGCAACCCTCAAAGAAGCTTTTCCATCAGCAACAAAAATCAGCGATAAAATCCCTTTCGCACATAAACAGCCCGAAATAAGAACCATTTAT 33300 467283
GP59            .................................................................................................... 33300 

JAM1            CAAGGTGAAGAAATTATAGGTTATGCCTTTGAAACTGTTGATGTGGTGGATGTGGCTGCTTACTCCGGTAAGCCAGTTAATCTACTGGTGGCCATAGATA 33400 
GP59            .................................................................................................... 33400 

JAM1            CCAAAGGCGAAATCAAAGTTGCTCGGGTGCTTGAGCATCATGAGCCAATTCTGCTGATAGGTATACCCGAGCAGCAGCTTTTTGACTTTGCCGATCAACT 33500 
GP59            .................................................................................................... 33500 

JAM1            AGTTGGTATGGATGTCAAGGACCATGTCATTGTTGGTCGTTCCGGTATAGAGAATGCTCACAGCATAGATTCCATCTCTGGAGCAACGGTCACGGTGATG 33600 
GP59            .................................................................................................... 33600 

JAM1            GTGGTCAATGAAGTTGTCATGCGTTCAGCCCGAAAAGTTGCCAGTCTGCTCAATATCGGTGGCATAACTGATAAAGTAACAAAGCAAGCGGCCTCTATTA 33700 
GP59            .................................................................................................... 33700 

JAM1            ACGAGGATGTTTTTAATGAAGCCGACTGGCCCCAATTAACCGGTGATGGTTCTATACGGCGTCTTCAGCTGAGTTATGGTGATATCGATAAGGCCTTTGA 33800 
GP59            .................................................................................................... 33800 

JAM1            AGGCACCAGAGCGGCTTTCAAAGGTAAAGCAAATGCAGCTGAACGGGAGCAAAAAAACTTTATAGACCTGTATTACACCCTTTTGGACATTCCGACCGTA 33900 
GP59            .................................................................................................... 33900 

JAM1            GGCAGAAACGTGCTGGGTGAGGATCAATATAATTGGCTAGTCAGTGAACTTAACCCCGGAGACCATGCCATTGCTGTTTTTGGCAAAGGGGATTATTCAT 34000 467983
GP59            .................................................................................................... 34000 

JAM1            TCAAGGGAACCGGTTACGTTAGAGGCGGTATTTTTGACCGGATTCAACTCCAGCAGGATGATAAATCCATTATGTTTCGCGATAGCGATTTTTACCGGCT 34100 468083
GP59            .................................................................................................... 34100 

JAM1            CAGTGATGTTTATTTGCAAGGGATGCCGAGTTTTTCCGAAATGGCTATTTTTATTATCCGTGATTCTTATGAGTTTGAACTCAGCCAAAACTGGCAACTC 34200 
GP59            .................................................................................................... 34200 

JAM1            GAATTACTGGTTCGCCGTCAGGTAGGTGCGCTGGACGGCGTTTTTACTCGTTTTTCCGGTGATTATAGCGTCCCTGAAAAATATCTTGAAAAACCACCAA 34300 
GP59            .................................................................................................... 34300 

JAM1            TGGAAATAACTGAAGCCCAGGATGAAGCTTTATGGGTGTCTGTCTGGCGTAATAAGGTTTTTCAGATAACCGTGCTTGGTATCAGCTTATTCATGTTGTT 34400 
GP59            .................................................................................................... 34400 

JAM1            CAGTGTGATCGTGATGCAGGATGTTTTGGTCAAGTATCCTCGATTTTTCCGCTGGTTCCGTCTGAGTTTTCTGGTGTATACCCTGTTTTTTATCGGTTGG 34500 
GP59            .................................................................................................... 34500 

JAM1            TACACACTGGGGCAGTTGTCGATTGTCAATGTGTTCACTTTCGTTCATTCCCTGGTGAATGGTTTCAAATGGGATACTTTTCTGCTTGATCCGATCATGT 34600 
GP59            .................................................................................................... 34600 

JAM1            TTATTTTATGGAGCTTTGTCGCCGTCACATTGTTGTTATGGGGGCGTGGAATTTTTTGTGGCTGGCTCTGTCCGTTTGGTGCATTACAGGAACTCATAAG 34700 
GP59            .................................................................................................... 34700 

JAM1            TGAAATAGCACGTAAATTGCGGGTGCGTCAGTTTGAATTACCTTTTGCCGTCCACGAGCGGTTATGGGCACTGAAATATCTGATCTTGTTGCTTTTGTTT 34800 
GP59            .................................................................................................... 34800 

JAM1            GCCATTTCGCTGGAATCGATGAGCGATGCCGAGCGCTATGCTGAAGTTGAGCCCTTTAAAACCGTAATTATGCTCAAATTTCAACGTGAATGGGCTTACG 34900 
GP59            .................................................................................................... 34900 

JAM1            TTTTCTATGCGGCAGTTTTACTAATCATCTCAGCGTTCACTCATAAAGCCTACTGTCGTTATATTTGTCCTTTGGGGGCGGCATTAGCTATTCCTTCACG 35000 
GP59            .................................................................................................... 35000 

JAM1            GTTTCGTCTGTTTGATTGGCTTAAACGTCGCAAGGAGTGTGGTACTCAGTGTCAAATCTGTGCAAACGAGTGTGAAATTCAAGCGATCCATCCCAGCGGT 35100 469083
GP59            .................................................................................................... 35100 

JAM1            AAAATTAATGCCAATGAATGTCATTGGTGTCTGGATTGTCAGGTCACTTATCACAACGAAACAAAATGCCCTCCTTTAATTAAGCGATATAACAAACGCC 35200 
GP59            .................................................................................................... 35200 => nosR

JAM1            ACAAAATGGAAAAAGGGTTTATCCCTATTACTATTGCAGCAGAAAAGAAATAAACTGAAGTCAATAAACTTGTATACCTGATCAGGAGTAGAAAGATGAT 35300 => nosZ
GP59            .................................................................................................... 35300 

JAM1            AGACAAAAAAGATAACAATCTTCCGAAGATTGATACGGAAGATAGTCATGCCAGTGAGCATGTCAAAATGAGTCGAAGGTTATTTCTTGGATCCTCTACT 35400 
GP59            .................................................................................................... 35400 

JAM1            GCAGTAGCAGGTGCTGTAGGTGCATTGGGAGCTGGCATCGGTTCAGTGTTCTCAGGTTCTACAATGGCTGATACACAAAATAATGGTGCTGGACATCATA 35500 
GP59            .................................................................................................... 35500 

JAM1            CCATTGGCCCTGGTGAGCTGGATGAATACTACGGTTTCTGGAGTGGTGGTCATCAGGGCGAGGTTCGGGTGCTTGGGGTTCCCTCAATGCGTGAACTGAT 35600 
GP59            .................................................................................................... 35600 

JAM1            GCGGATACCAGTGTTCAACATAGATTCAGCGACTGGTTGGGGGTTAACTAATGAGAGTAAAGAGATACTGGGTGATCAACTGTATCAAAATGGCGATTGT 35700 
GP59            .................................................................................................... 35700 

JAM1            CATCATCCACATATTTCCCTAACGGACGGTCGTTATGACGGTAAGTATCTTTTCATTAATGATAAGGCAAATACCCGGGTAGCCAGAATCCGTCTGGATA 35800 
GP59            .................................................................................................... 35800 

JAM1            TTATGAAAACGGACCGTATTGTTCACGTTCCAAATGTCCAGGCTATTCATGGCTTGAGACTGCAGAAAGTACCCCATACAAAATATGTATTTTGTAATGC 35900 469883
GP59            .................................................................................................... 35900 

JAM1            TGAGTTTCTAATTCCACATCCTAACGATGGTAGCGAATTTACACTGGAAAATAGCTATACGATGTTCAACGCCATTGACGCTGAAACCATGGAGGTAGCA 36000 => nosZ
GP59            .................................................................................................... 36000 

JAM1            TGGCAAGTTATAGTTGATGGTAATCTGGATAATATGGATGCAGACTATACTGGAAAATATGCCGCATCGACCTGTTATAACTCCGAAAAAGCCCTGGATT 36100 470083
GP59            .................................................................................................... 36100 

JAM1            TAGCTGGCACAATGCGTAATGAGCGTGACTGGGTAGTCGTCTTTAATATCGAACGGATCGAAGCTGCTGTCAACGCAGGTAATTTCAAAACTATCGGAGA 36200 
GP59            .................................................................................................... 36200 

JAM1            TTCAAAAGTGCCAGTTGTGGATGGTCGCAAAGGGAGCGAGTTGACACGCTATATTCCGGTGCCAAAAAATCCGCATGGTCTCAACACTTCGCCTGATGGT 36300 
GP59            .................................................................................................... 36300 

JAM1            AAATATTTTATTGCAAATGGTAAATTGTCTCCGACCTGTTCAATTATTGCGACTGACAGATTGGACGATTTGTTCGATGATAAAATTTCTGAGCGTGATC 36400 
GP59            .................................................................................................... 36400 

JAM1            CGATTATTGGTGAACCAGAGCTTGGACTTGGCCCGCTACATACTACGTTTGATGGACGGGGTAATGCTTACACCACGCTGTTTATTGATAGCCAAGTTGT 36500 
GP59            .................................................................................................... 36500 

JAM1            GAAATGGAACATCGAAGACGCAATCAAACATTATCAAGGCGAAGATGTTAGCTATATCCGTCAGAAGTTGGATGTACACTATCAGCCCGGACATAACCAT 36600 
GP59            .................................................................................................... 36600 

JAM1            GCTTCATTGACCGAATCACGTGATGCAGACGGAAAATGGATGGTGGTTTTATCCAAATTCTCCAAAGACCGTTTTCTGCCAGTCGGGCCGTTACATCCAG 36700 
GP59            .................................................................................................... 36700 

JAM1            AAAATGATCAGTTGATTGATATTTCTGGCGATGAAATGAAGCTTGTACATGATGGTCCTACATACGCTGAACCACATGACTGCATTCTGGTACGCCGGGA 36800 
GP59            .................................................................................................... 36800 

JAM1            TCAAATCAAGACAAAAAAAATCTGGGATCCACAAGATCCATTTTTTGCCAGCGCACGCAAACAAGCTGAGAAAGATGGCGTCGAACTTGAATTTGATAAT 36900 
GP59            .................................................................................................... 36900 

JAM1            ACGGTTATCCGTGATGGAAACAAGGTACGTGTTTATATGACATCTGTTGCTCCAGTGTTTGGGCTGAATGAATTCAAGGTTAAGCAGGGAGATGAAGTAA 37000 
GP59            .................................................................................................... 37000 

JAM1            CGGTTTATGTCAGCAATCGTGAGATGGTTGAAGATGTTACGCATGGTTTTTGTATGGTGAACCATGGCGTAAATATGGAGATCAGTCCGCAACAGACCTC 37100 471083
GP59            .................................................................................................... 37100 

JAM1            TTCAATAACCTTTATTGCGTCAAAACCAGGGGTTCATTGGTACTATTGTTCATGGTTCTGCCATGCCATGCATATGGAAATGACTGGACGCATGATCGTC 37200 => nosZ
GP59            .................................................................................................... 37200 

JAM1            GAAAAGGTTTAAGAAAACCGTGGACGGAAAGTTATTTCCGTCCACTCTTTGCTCAATTAGCTACTGGTATACGAATATAACAAGAGGATCTTTGTGGTTA 37300 => nosD
GP59            .................................................................................................... 37300 

JAM1            ATTTAAAGCGGCGACATTTCAAGGGATGCCAAAGCTTTCATTATTTATTAGCGGCACTTTGTTTTTTTGTTTCCTTCGACTCAAATGCTGAGTTGCAGAC 37400 
GP59            .................................................................................................... 37400 

JAM1            AAACTTGGATCAGATCGAACCGGGAGGAACCATTACCTTGCCTGCGGGAGAAATATCCTCTATGGTTCTGCGCGTTCCGAATGTGACGGTATCATGTGAT 37500 
GP59            .................................................................................................... 37500 

JAM1            AAAAATACGGTTATTGATGCAGAAGGTCAGGGCCATGCTGTTGAAATTCTCGCTGCTGGAATTTCGTTCAGTGATTGTCAAATTCGCAATTGGGGGCAGG 37600 
GP59            .................................................................................................... 37600 

JAM1            ATCTCACCAAGTTAGACGCGGGAATTTTTGTATCCCGTCAAGCGACAGGATCAGTTATTCATAATAATCGTCTGACAGGGCCAGCTTTTGGAATCTGGGT 37700 
GP59            .................................................................................................... 37700 

JAM1            GGATGCAACGGCTGACGTGGTTGTGCGAAACAATTATATTCAGGGAGATGCCGGGATACGCTCTCAAGATCGGGGAAATGGTATTCATCTTTTTAATACT 37800 471783
GP59            .................................................................................................... 37800 

JAM1            TCCGGCGCTCTCATTGAAGAAAACAAAATCATCCAGACCCGGGATGGCATTTATATTGAAGCTGCAAACAACAACACTATACGCAACAACCTGATGACAT 37900 471883
GP59            .................................................................................................... 37900 

JAM1            CATTACGCTATGGTATTCATTACATGTATTCCATGGATAATTTATTGGAAAATAATATCACTCGAAATACTCGTACGGGCTATGCCTTGATGCAAAGTAA 38000 
GP59            .................................................................................................... 38000 

JAM1            ACGGCTGAGGGTTATCGGGAACCGCTCTGAGAATGATGAGAATTACGGTATTTTGCTGAATTTTATCACCGACTCAATTTTTCTTAACAACGTGGTTGAA 38100 
GP59            .................................................................................................... 38100 

JAM1            GGTGTTTCTCAAGGGCAGACCGCTGGATTGGATATTTCCGGTGCAGAAGGTAAGGGACTATTTGTTTATAACTCTTTGTATAACCGCTTCGAAAGCAATT 38200 
GP59            .................................................................................................... 38200 

JAM1            TTTTTGTCGACAACAATATTGGAATCCATCTGACAGCTGGATCGGAAGATAATAAATTCAAAAATAACGCTTTTATCAATAACACTAATCAAGTGAAATA 38300 
GP59            .................................................................................................... 38300 

JAM1            TGTCTCTAATCGGCTGCAGGAGTGGTCAACAGATGGTGTTGGTAACTATTGGAGTGATTATCTTGGCTGGGATTTAAATAAAGATGATATTGGCGATACG 38400 
GP59            .................................................................................................... 38400 

JAM1            GCATATGAACCCAATGACGGTATTGATCGTTTGTTATGGAAATATCCTGACGCCAAATTACTGATCAACAGCCCATCGGTTATGTTGTTGCGCTGGGTGC 38500 
GP59            .................................................................................................... 38500 => nosD

JAM1            AAAAACAGTTTCCGATATTGAAATCTCCCGGTGTCAAAGATAGTCATCCATTAATGACTTTACCCGCTGAGCTCACGCCATGATTATTGTTGATTTATCA 38600 => nosF  fnr
GP59            .................................................................................................... 38600 

JAM1            CAAGTGGAAAAACATTACCGTGGTGTACACGCTCTGCAACAACTTGATTTGCAGATCCAGCAGGGCGAAATATTAGGCCTTTTCGGTCATAACGGTGCTG 38700 
GP59            .................................................................................................... 38700 

JAM1            GTAAAACGACAACCATTAAATTGATCTTAGGCCTGATCAAACCTAGCCAGGGTGAAGTACGGGTATTTGATAAAAATCCCGCTGGCAGTCACGCCTATCA 38800 
GP59            .................................................................................................... 38800 

JAM1            GATACGCCGTCAACTCGGTTTTTTGCAGGAAAATGTCAGTTTTTATCAGCAAATGACTGGATTGGAAGTGCTGAATTATTTTGCAAAATTAAAAGGTTGC 38900 472883
GP59            .................................................................................................... 38900 

JAM1            ACAGCGAGACAGCCCCGTGATTTGTTGGCACAAGTTGGGCTGGAACAGGCAGCAGATCGCCGAGTAAAAACCTACTCAAAAGGTATGTGTCAGCGTTTAG 39000 
GP59            .................................................................................................... 39000 

JAM1            GGCTTGCACAAGCCTTGCTGGGTGCACCAAAACTATTATTATTAGATGAACCTACGGTTGGACTGGATCCGCTCGCCACACGCGATTTTTACAGCAGTAT 39100 
GP59            .................................................................................................... 39100 

JAM1            TGACGCTTTAAAACAAGCGGGTAGCACCATTGTGCTATGTTCACACCTGTTGGCTGGAGTTGAGAAGGTCATTGATCGGGCTTTGATTTTAAAACAAGGA 39200 
GP59            .................................................................................................... 39200 

JAM1            CGCGCATTGGCTGCCGGCAGCCTAAGTGAACTACGCCAACAGGCTGATTTACCAGTAAATATTGAACTCCAGGGTCAAGGTCTGCAACTTCCACAAAATC 39300 
GP59            .................................................................................................... 39300 

JAM1            TATTCGCTGACTTTCATCGTACTGAAAAGGGGATTGAATGCCAGATCAGTCAGCAACAGAAAATGCATTTATTACCACAACTGCTTTCGCTGCCAGGACT 39400 => nosF
GP59            .................................................................................................... 39400 

JAM1            AGAAAACATCGAGATCCATATGCCTTCTCTGGATGATGTGTACGCGCATTTTAATCAACCTTCTTCTGGAACCGTCTGATGTCAAACGTATTGTTAACCG 39500 =>nosY
GP59            .................................................................................................... 39500 

JAM1            TTGCAGGCAAGGAGTTTCGTGATGGCCTTCGCAATCGCTGGGTGGTATCCATTAGCCTTATTTTTGCCATTCTTGCCACGGGATTGGCTTATTTTGGCTC 39600 
GP59            .................................................................................................... 39600 

JAM1            CGCAGCCTCTGGGCAGGCCGGATTTTCTTCGCTTTCTGCCACTTTGGTCAGTCTCGCCACCCTTGCAGTGTTTCTTATCCCATTAATTGCTCTCATGCTT 39700 473683
GP59            .................................................................................................... 39700 

JAM1            GCGTATGACAGTGTCGTTGGCGAAGATGAACAAGGCACCTTATTATTGTTAATGACTTATCCGATGAAACGCTGGCAGTTTATTGCGGGTAAAATGCTTG 39800 473783
GP59            .................................................................................................... 39800 

JAM1            GACACGGAATGATCCTGGCATGTGCCAGCATTACAGGGTTTGGTTTGTCAGCGTTAGTGACAGGACTTTTTTCCGATAACACCTCTTGGTCAGAGTTGTT 39900 
GP59            .................................................................................................... 39900 

JAM1            TGATGCCTATGTTTTTTTTATTCTTTTTTCGGTCTTGTTGGGATGGATATTTATTGCATTTGCTTATGTTATTAGTGTGACGGTTACTGAAAAATCGAAA 40000 
GP59            .................................................................................................... 40000 

JAM1            GCTGCGGGGCTCGCATTAATCGTCTGGTTTAGTGTCGTCTTGATGTTTGATTTGATGTTACTGGGGTTGCTGGTGACGACCGAAGGCAATGTCAATGCGG 40100 
GP59            .................................................................................................... 40100 

JAM1            AGATGTTTCCCTATCTATTGCTACTCAATCCAACTGATATCTATCGCTTAGCAATTATTCAGTATTTTGCCGACCAACAGCTAAGCGGACTGATGGCCGT 40200 
GP59            .................................................................................................... 40200 

JAM1            GGCACAACAGGCGCAATTCACTACCCGCATATTGTTTTGCGGAATGGTGGTTTGGCTGTTGATTCCGTTACTGTATACCTTGTTCAAGTTTAATAAGAGA 40300 =>nosY
GP59            .................................................................................................... 40300 

JAM1            ACATTATGACGCTAAGCAAAAAAATACGACCGTTAATGGTGGCGATAGTTTTGATTATCACTGCCTGTGGTGAATCACCTTCAGAACTTACATTGGTTCA 40400 => nosL
GP59            .................................................................................................... 40400 

JAM1            GCACGCTGTCAAAGTCGAAAGCGGTGATGAGTGTCATCAATGTGGCATGATTATCAAGCACTTCCCTGGACCAAAAGGACAGTTGTATCAGCGCGGCACT 40500 
GP59            .................................................................................................... 40500 

JAM1            GAGCAAGTGCTGAAGTTTTGCTCTACTCGCGATATGTTTGCCTATTTACTGGATCCGGAACATCAGCACGCCATACAAACAGTATATGTTCATGATATGG 40600 
GP59            .................................................................................................... 40600 

JAM1            CTCAAACATCATGGGATGCCCCAGATGATGAGTTCTATATTGATGCCCGCCAAGCGTGGTTTGTTATTGGTCATCGTCAACAAGGGGCAATGGGGCCGAC 40700 
GP59            .................................................................................................... 40700 

JAM1            CTTGGCCAGTTTTGCGGAAAAAGAAATTGCCAACAGGTTTGCTAAAGAATACGGCGGCACAGTGCTGGCTTTCGAGGCGATAACACAAGCAAAAATATCT 40800 474783
GP59            .................................................................................................... 40800 

JAM1            GGTCGAGAGACCAAATAGAGAAAAAATGAAGAAAGTGTAAGAACAGATTAACGCTCTTTCCACAGGGGATTAAATTAATAACAGTTGATGTTCTGTTTTT 40900 
GP59            .................................................................................................... 40900 

JAM1            TTAGCCTATATCGGGATTGTTTATCTTTCATATCCTCCAACGCTGCTGACGGCTATTTTTGTGTTTGATGAGGCGCTAAGCAGGAAGTTTAGCCTTCAGT 41000 
GP59            .................................................................................................... 41000 

JAM1            CTGGAACTCGTTTGAACGATTTATAAATACAGAGATGTGTTTCATCAACGCAGTGGGTTGCGAAAACAGTCTCAACTTTACAGGATGTGATTGAAACCCA 41100 
GP59            .................................................................................................... 41100 

JAM1            ACCAAATCAGCAAACAGAGAGAAAAGATGGCGAGCTGTCTTAGGGGTTCTCTATCCATGGGGCTTATGCCTGAAAATATTTAGTTGCAGACTGCAAAGGC 41200 
GP59            .................................................................................................... 41200 

JAM1            TATTTACTGACGTTTTTGTAATCATTCTAAACAAGTTAATACCTTAACCACATGGAACCCCAGTAAAAAACCTCGATAGCACTGAGAGCAACCCTTGGAA 41300 
GP59            .................................................................................................... 41300 

JAM1            TGGTTTGTAGACCGACCAATATGAACAATAATTGACATTTTTTTGCTCTTGCTCAGAGTCTGATGTTTCTTTTATATGGTGTACTCGAACTGACCTAAGT 41400 fnr
GP59            .................................................................................................... 41400 

JAM1            ACATCATCCAGCTTAAGTTTTGCCAAAACTTGATCCAGATCAATTACACAACATGTTGTGCTTTTATTTTTAATAAACACAATATATTGTGTTTTATGTC 41500 fnr(coli)/anr
GP59            .................................................................................................... 41500 

JAM1            TTTTTGATTAACCGAGTGTGCTGCGTTAAATAAAGACTAAAGAGGTAGAAATGAGATGACATCTTGTCGCTGTTCGGTGCTGAGTTGCTGACGTTTCTCA 41600 475583
GP59            .................................................................................................... 41600 

JAM1            ACAGAGAAAATGAATTAGGAAGGGGATAAAAATGTACACAGACATAGCCACTGCCACAGACGAGTTATCGGTAGGCAAACTGCCTGACATGGTGATCAAG 41700 => RiboN Redase LSU
GP59            .................................................................................................... 41700 

JAM1            CGTGACGGCAGACAGGCCCGGTTTGATGCTGAAAAAATCCTTTCGGCGATTCATCATGCAGGAATGGCCACTGAAGAATTTTCTATAGAAGAAGCGCGCC 41800 475783
GP59            .................................................................................................... 41800 

JAM1            AGTTAACTCTGCAGGTATTAAAAGTGCTGCATCATCGTTACGCAGAAAGCTTGCCCGAGATAGAGCAAATTCAGGACGTGGTTGAACAGGTTTTGATTTC 41900 
GP59            .................................................................................................... 41900 

JAM1            TTCTGATTATTTCCGTACCGCACGTGCTTATATTGTTTACCGAGAACAGCACGGTAAATTACGGCATGATCAAACCACGATAATCAACGTCGAAACCTCA 42000 
GP59            .................................................................................................... 42000 

JAM1            ATGAATGAATACCTGCAGCAACTCGACTGGCGGGTCAATGCCAATGCCAACCAGGGTTATTCTCTGGGCGGTTTGATTTTGAACACTGCAGGCAAAGTTA 42100 
GP59            .................................................................................................... 42100 

JAM1            CTGCTAACTACTGGCTCAATCATGTTTATCCTGCGGAGGCAGGTTTAGCACACCGTGAAGGCGATCTTCATATTCATGATCTTGATATGTTGGCGGGTTA 42200 
GP59            .................................................................................................... 42200 

JAM1            TTGCGCCGGCTGGTCTTTGCGTAACTTGTTAACGGAAGGCTTCAATGGGGTGCCTGGCAAGGTTGAAGCCGGACCGCCTAAGCGTTTTTCCAGTGCCTTG 42300 
GP59            .................................................................................................... 42300 

JAM1            GGCCAAATGGTTAACTTTCTTGGAGCACTGCAGAATGAATGGGCCGGAGCTCAGGCGTTCAGCTCATTTGATACTTATCTGGCGCCTTATGTTCGCAATG 42400 
GP59            .................................................................................................... 42400 

JAM1            AAAATTTAACCTATGATGTGGTGCGTCAAGGGATACAAGAATTTATCTATAACCTTAACGTACCTTCACGTTGGGGAACGCAAACACCTTTTACTAACCT 42500 
GP59            .................................................................................................... 42500 

JAM1            GACTTTTGACTGGGTGTGTCCCGAAGATCTGCGGGAGCAGATTCCGGTAATTGCTGGTAAAGAAATGCCATTTTTTTATGGTGATCTGCAGACAGAAATG 42600 
GP59            .................................................................................................... 42600 

JAM1            GACATGATTAATCAGGCTTATATTGAGGTCATGATTGCCGGTGATGAAAAAGGACGTGTTTTCACCTTCCCTATTCCTACATATAACATTACAAAAGACT 42700 
GP59            .................................................................................................... 42700 

JAM1            TTCCCTGGGAAAGTGATAACGCTGAACGGTTATTTGATATGACCGCCAAATACGGCTTACCTTATTTTCAGAATTTTATTAATTCCGATATGGAACCGGG 42800 476783
GP59            .................................................................................................... 42800 

JAM1            CCAGGTTCGCTCAATGTGTTGCCGACTGCAACTGGATCTAAGAGAGTTGTTAAAACGAGGTAATGGTTTATTCGGTTCTGCTGAACAAACAGGGTCTATT 42900 
GP59            .................................................................................................... 42900 

JAM1            GGTGTGGTTACAATCAATTGTGCTCGGCTTGGCTACCTTTACAAAAATGATGAACCAGCCTTAATTAAGAGCCTTGACCGGCTGATGGAGCTGGCCAAGA 43000 => RiboN Redase LSU
GP59            .................................................................................................... 43000 

JAM1            ACAGTCTGGAAGTCAAACGCAAGATGATTCAGCGCTATATGGATGGTGGCCTATATCCTTATACCAAACGTTATCTGGGAACCTTGAGAAATCATTTTTC 43100 
GP59            .................................................................................................... 43100 

JAM1            CACTATTGGAGTGAACGGAATTAACGAGATGATCCGTAACTTCAGTGATGATGCCCATGATGTCAGCGATGAGTGGGGCCAGCAATTTGCATTAAGACTA 43200 
GP59            .................................................................................................... 43200 

JAM1            CTCACGCATGTTCGAGAAAAATTGATTGTTTTTCAGGATGAAACCGGGCATATGTATAACCTTGAAGCCACTCCTGCCGAGGGTACGACCTATCGTTTTG 43300 
GP59            .................................................................................................... 43300 

JAM1            CCAAAGAAGATCGCAAGCGTTTTGCAGATATTATTCAAGCCGGTACTGAAGCTGCACCTTATTACACCAACTCATCACAGCTGCCAGTGGGGCTGACAGA 43400 
GP59            .................................................................................................... 43400 

JAM1            TGATCCCTTTGAAGCTTTATCGCTGCAGGATGAGCTGCAGACACAATATACAGGCGGTACGGTTCTTCACCTTTATATGAATGAACGGATCTCGGATTCA 43500 477483
GP59            .................................................................................................... 43500 

JAM1            CTCGCCTGTAGCACATTGGTCCGACGTGTGCTGGAACGTTTCCGACTTCCTTATATCACGATTACGCCGACATTCTCCATTTGTCCCAAGCATGGTTATC 43600 => RiboN Redase LSU
GP59            .................................................................................................... 43600 

JAM1            TTGCTGGGGAACACGAGTTTTGTCCGCATTGTGATCAGGAACTACTCGCGGAAAAACACAACGAACAAGTTGTGGCTTAATCATTTATCAACAAGGAGTA 43700 fnr
GP59            .................................................................................................... 43700 

JAM1            ATGACATGACAACTAACAATCTTATGACAATAGAGCTGCGTGAAGAAGAACGTCAACGCTGTGAAATCTGGACCCGGGTAATGGGATATCATCGTCCGTT 43800
GP59            .................................................................................................... 43800 

JAM1            GACGTCATTCAACCCAGGCAAACAAAGCGAGCATGCAGAACGTCGGCATTTCATTGAATCTTCGGCCAATAGCTGATTGTAAAGGAGACGCCATTTAATG 43900 => => RiboN Redase
GP59            .................................................................................................... 43900 

JAM1            GGACTAAAGATTGGTGGGCTGACTTCGTTTACGACCATCGATTTTCCAGGCCATTTGGCTGCAGTCGTTTTTTTTCAAGGCTGTGCTTGGCGTTGTCCCT 44000 
GP59            .................................................................................................... 44000 

JAM1            ATTGCCATAATCCGCACCTGCTTGAGGTTGATAAGCAAGGTGATGATTGGGAGAAGTTGCGAGCATTTTTGCAACCAAGACGGGGGTTTCTTGACGGGGT 44100 
GP59            .................................................................................................... 44100 

JAM1            TGTTCTGAGCGGTGGTGAACCGCTACTGCAAACCGGATTAGCAGATGCCATTAACGGCATCAAAGCTATGGGGTTTAAAGTGGCACTTCATACCGCCGGA 44200 
GP59            .................................................................................................... 44200 

JAM1            GCCAACCCTCAAAGGCTTGAAAAATACCTGTCAATGCTTGACTGGGTTGGCCTGGATATCAAAACGACCTTTACAGAGTATTCCCAAATTACCGGAATAA 44300 
GP59            .................................................................................................... 44300 

JAM1            AAGGAAGTGGTGAACAAGTTCAGCAAAGTCTAAAGTCGATTCTGGCCAGTGGTATTGCCTATGAAGTGCGAACTACGGTTGATCCGAATTTCTTTACCCA 44400 
GP59            .................................................................................................... 44400 

JAM1            CAAAACAGTTATTGAGTTGGCTGAGAGCCTGGCAGCAGAAGGTGTTACGCACTATGCTTTGCAGGAATGCAGACCAGTTGCAGGCTATCAGATGCAAAAA 44500 478483
GP59            .................................................................................................... 44500 

JAM1            GCATCTTTATTTCATGACGGTTCACTGGCAGCACAACTTGAAAAGATGTTTTCCCATTTTACGTTACGCTATGGCTAATATTGAATGATGTAATTATCTG 44600 
GP59            .................................................................................................... 44600 

JAM1            ATTTTTATATTAAAATGTTTCGCTGTAGTTTTTTCAGTAACGACGTGATCAGCACAGAAATTATTAGAAAAGAAGCTTATTTTTGTTCCGGCCGGTGACG 44700 
GP59            .................................................................................................... 44700 

JAM1            GTACGGTTATTTTTCGGAGGTTTGACAGTGGCACAAAAGACCACATCTTTTTCAGTAAAAACAGGATTGGCCGAAATGCTTAGAGGTGGCGTGATTATGG 44800 => Pyr-Glu NTrfase
GP59            .................................................................................................... 44800 

JAM1            ATGTCACCAATCCACAACAGGCAAAGATTGCCGAAGATGCCGGAGCTGTTGCGGTCATGGCTCTGGAAAGAATCCCTTCTGACATCAGAAAGGATGGTGG 44900 
GP59            .................................................................................................... 44900 

JAM1            CATTGCGCGGATGAGTGATCCGGAGATGATTCGGGAAATTCAGGAAACTGTCACCATTCCAGTGATGGCAAAATGTCGGATTGGTCATTTTGTTGAAGCC 45000 
GP59            .................................................................................................... 45000 

JAM1            CAGATATTACAGGCGCTGGAAATTGATTTTATTGATGAAAGTGAAGTACTGACACCCGCAGATGAAACCAATCATATTTGGAAGCATGAATTTAATGTAC 45100 
GP59            .................................................................................................... 45100 

JAM1            CTTTTGTGTGTGGTTGTCGGAACCTGGGTGAAGCGTTACGGCGTATCGGTGAAGGGGCTGCTATGATCCGTACCAAAGGTGAAGCTGGCAGTGGCAACAT 45200 
GP59            .................................................................................................... 45200 

JAM1            TGTCGAAGCCGTTCGTCATATGCGTCAGATTCAAAACGACATGAAAAACCTCATGGGGTTACGAACCGAGGAATTAATGGCACAAGCTAAAATATTGGGC 45300 
GP59            .................................................................................................... 45300 

JAM1            GCACCTTTCGATTTGGTTCGTCTGGTAGCTGAAACTGGGAAATTACCCGTACCTAACTTTGCCGCAGGTGGAGTTGCTACTCCTGCTGATGCTGCTCTTA 45400 479383
GP59            .................................................................................................... 45400 

JAM1            TGATGCAATTAGGGGCCGAGACAGTGTTTGTCGGATCGGGTATTTTCATGAGTGAAGACCCACAACGACGTGCCAAAGCCATTGTGGAGGCCGTAACCTA 45500 479483
GP59            .................................................................................................... 45500 

JAM1            TTTCGAAGATGCACAAAAGCTGGCTGAAATATCGGCTGGTTTAAAAAGTGCCATGAAAGGCCTTGATATTGCAGAAATACCAGCTGAGCAAAGGTTACAG 45600 => Pyr-Glu NTrfase
GP59            .................................................................................................... 45600 

JAM1            GAGCGGGGTTGGTAAGTGAAAATCGGTCTGCTGGCATTACAGGGTGATTATGAAAAACATAGTCAGATTTTATCCCGCCTGGATCTGCAAACTGTTCTGG 45700 => Pyr-Glu NTrfase
GP59            .................................................................................................... 45700 

JAM1            TTCGTTACCCACAACAATTGAACGATGTGGCAGGACTGGTTATTCCTGGTGGAGAGTCGACGACTATGAGCAAACTGATCAATGCGAATGGATTCTGCCA 45800 
GP59            .................................................................................................... 45800 

JAM1            GCCGTTGGTTTCATTCGCACAGCAATTTCCGATATTGGGCACCTGTGCTGGTTTGATAATGATGGGCAGGCTGCAACAGCCTGACCCACGTATTGAGACA 45900 
GP59            .................................................................................................... 45900 

JAM1            TTAGACCTGATAGATGTTACGGTAACGCGTAATGCTTATGGACGACAATTGGCCTCTTTTGTTGATGATTTGACGGTGGAGGTGGCGGATCACCAGCAAA 46000 
GP59            .................................................................................................... 46000 

JAM1            CCGTTCCTGGTTGCTTTATCCGGGCGCCCAAGATTAATACCATTGGGTCCAATGTTGAAGTGCTGGCACGATACAACAATGACCCCGTTGCTGTTCGTCA 46100 
GP59            .................................................................................................... 46100 

JAM1            AGATCGCCATATCGGCCTGACGTTTCATCCAGAACTAAATAATGTCACTCTATTTCATCAATTAGCTTTTCGTGGCGGCTGAAATGAATGGTAGTAAGTT 46200 
GP59            .................................................................................................... 46200 

JAM1            ATGACGATAAAAAAAGCAAAAATCGGTATTGTGACGGTCAGTGATCGGGCTAGCAAAGGTGTTTATGATGATCTTTCCGGGCAGGTCATTATGGCGACAT 46300 => mogA
GP59            .................................................................................................... 46300 

JAM1            TGGATGAATTTCTGACATCACCCTGGCAACCGGTTTATGAAGTGATTCCAGATGAAAAAGACGTGATTTGTGCAACGCTGATTAAAATGGCTGATGAATT 46400 
GP59            .................................................................................................... 46400 

JAM1            GGGTTGTTGTCTGATCGTGACGACTGGCGGTACCGGACCTGCAAAACGTGATGTTACGCCAGAGGCGACCGAAGCCGTATGCGATCGCATGATGCCCGGA 46500 480483
GP59            .................................................................................................... 46500 

JAM1            TTTGGTGAATTAATGCGGGCTGAATCGTTGAAGTTTGTTGCTACTGCCATTTTATCCCGCCAGACAGCGGGGTTAAGAGGATCAAGTCTTATCGTCAACC 46600 
GP59            .................................................................................................... 46600 

JAM1            TTCCCGGTAAACCGAAAGCAATCCGAGAATGCCTTGATGCTGTTTTTCCAGCAGTACCTTACTGTATCGATCTGATGGAAGGGCCCTACCTAGAATGTGA 46700 
GP59            .................................................................................................... 46700 

JAM1            CGAAACAGTTATTAAACCATTTCGACCCAAAAACTAGGAGTCTGTTGCTTCTAAAAAACTACTGTGGAAAAGCCTCAACAACGTCAGCCATCAAAGTTAG 46800 <= Put-Lip carrier
GP59            .................................................................................................... 46800 

JAM1            CGCATGATTAAAAATGGATGGTAATTCATCCATATCAACACTATCAATTAAATTCTTAATCTGTAGACCCAATTCGGTATTGCCCTCAATAGACAAGCGT 46900 
GP59            .................................................................................................... 46900 

JAM1            CGTTGAAAAAACAGTGTGTCCGGGTCTTCACGGCGACCAGCTAACAACAGAAAATCTTTACTGTTGCCGGAAAAAGTAGTGTCAGCACGGTCGCTGCCAG 47000 
GP59            .................................................................................................... 47000 

JAM1            AACTGAATGATAAACGATTTTGCTTTAACGTCACCTGCCAATCATAAGCCAGATCTTTTATTTGTATTCGTAACAGCGTATTTTCCAGAAAGCTTAACTC 47100 
GP59            .................................................................................................... 47100 

JAM1            ACCTTCGAGCAGCTGTTGTTTGAATATAACATTGGACACTTTTTCCACAACGATGAACTGTGCGGCTTGCGGCAACAGTCTAAACGGTATCCGCCAGGGC 47200 
GP59            .................................................................................................... 47200 <= Put-Lip carrier

JAM1            AGGTATTGAATGCCAGGCAATAAAGCCAATTTTTCCGGTATGGATGAAACAAGTTTTGTTGGCATCGAAACCATAGTCAGTTGTTCCTGAAGAGCCTGAT 47300 
GP59            .................................................................................................... 47300 

JAM1            ATGTAAATGAAATGACTATGATGATGGTTTTACGAGGTAAATAACCATGACCTAGATCAATAGATGGGCAACTAGCCATCATTTACGCATTAGAAACTTA 47400 481383
GP59            .................................................................................................... 47400 

JAM1            TATTATGTTGTTACAAAAATATGGAGGAAAAAATGGAGCTGGTCTGTCCTGCGGGGAATATCTCAATGTTAAAAGCCGCAGCCGAGCATGGTGCTGACGC 47500 => protease
GP59            .................................................................................................... 47500 

JAM1            AATCTATATCGGTTTCAAAGATGACACTAATGCCAGGCATTTTGCCGGCTTGAATTTTCAAGATGGTGCCGCACAGAAAGCCGTTAACTATGCACATGAT 47600 
GP59            .................................................................................................... 47600 

JAM1            CACGGTAGCCAGGTCTATGTCGCTATTAATACCTATCCACAACCGACCGGTTGGTCGAGATGGCAAAAAGCAGTTGATGTCGCAGCAGATATAGGTGTTG 47700 
GP59            .................................................................................................... 47700 

JAM1            ATGCTTTAATTGTTGCTGATATTGGTGTGCTGGCCTATGCGGCAGAAAAATATCCGCATCTGGCGTTGCATTTGTCGGTACAGGGTTCAGCTACCAACGC 47800 
GP59            .................................................................................................... 47800 

JAM1            TGAGGCATTACGATTTTATCAGCAACAGTTTGCTATTCGCCGTGCCGTCATACCGCGTGTATTGTCGATGGCGCAGGTAAAACGATTGGCCGAAGACAGT 47900 
GP59            .................................................................................................... 47900 

JAM1            CCTGTTAAGCTTGAAGTATTCGGTTTTGGCAGCTTATGCGTCATGGTGGAAGGGCGTTGTTTATTATCGTCTTATGCAACCGGTGAATCCCCAAATACTT 48000 
GP59            .................................................................................................... 48000 

JAM1            GCGGTGCCTGCAGTCCGGCGAAAGCTGTGCGCTGGGTGGAAACGCCGGCAGGCACAGAGAGCCGATTAAATGAGGTATTGATTGACCGTTTTGCAGTGGG 48100 
GP59            .................................................................................................... 48100 

JAM1            TGAAAAAGCCGGCTATCCGGTGGTCTGTAAAGGACGGTTTAATGTTGACGGCCATACCTATCATGCCTTGGAAGAGCCCACCAGTTTAAATACGTTGGCA 48200 
GP59            .................................................................................................... 48200 

JAM1            TTATTGCCTGAGCTGTTGGCAGCAGGGGTCAGTGCCATAAAAATAGAAGGTCGTCAGCGCAGTACCGCATACATTACCAAGGTGGTCAAAGTCTGGCGGG 48300 
GP59            .................................................................................................... 48300 

JAM1            AAGCGATAGATAGTTGTCAGTCAAACCCGCAGTCGTATGCGGCAAAAGCACAGTGGATGCAGGCGCTTGCTGATGTATCTGAAGGTACACAAACCACTCT 48400 482383
GP59            .................................................................................................... 48400 

JAM1            GGGCGCTTACAACCGCCCCTGGCAATAAATAAAGTCAGGTCAAGCACAAGACTTTTGGAGTTTTCATGCGTTTATCATTAGGAGCAATTCAGTATCTGTG 48500  => protease
GP59            .................................................................................................... 48500 

JAM1            GCCTCGGCAAACAGTGGAGGATTTTTATCAATTACAGCTGAAGAGTCCAGTTGATATTATTTATCTGGGTGAAACTGTGTGTGCCAAAAGAAGGTTTCTG 48600 
GP59            .................................................................................................... 48600 

JAM1            AAAACAGCAGAATGGATTGAACTGGCGAAGCAACTGGCACAATCAGGAAAGCAAATCGTGCTGTCTACACTGACTTTGATTGAAGCCAGATCCGATATGG 48700 
GP59            .................................................................................................... 48700 

JAM1            CAAGCGTCAAACGTTTATGTAACAACGGTGACTTATTGGTAGAAGCGAATGACATTGGTGCTGTGCAATTAATGGCCGAGCAGACGCTTCCTTTTGTCGG 48800 
GP59            .................................................................................................... 48800 

JAM1            CGGGCCCAGTTTAAATATCTACAATGCCCATACACTTAAACAGTTATATAAGCAGGGTTTAATCCGCTGGGTCATGCCGGTTGAACTGGATGCGGAAACA 48900 
GP59            .................................................................................................... 48900 

JAM1            CTGAAAGCGATATTGGCTGATGCGGAATCCTTGGGTCTGGGTGGAAAGATTGAAACTGAAGTTTTCTCATTTGGTCGCATGCCGCTGGCTTATTCAGCCC 49000 
GP59            .................................................................................................... 49000 

JAM1            GTTGTTTTACCGCCCGTTATCGTCAGCTGCCCAAGGATGACTGTCAACTTGTGTGTCAGGATTATGCTGAAGGCTTGCCAATGGATAGTCAGGAAGGTCA 49100 
GP59            .................................................................................................... 49100 

JAM1            AGCGTTTTTCACCATTAACGGTATTCAGACACAATCAGGCCGCATTCAGCATTTATTGCCGCATTGGCAACAAATGCAACAGATTGGTGTAGACATTATG 49200 483183
GP59            .................................................................................................... 49200 

JAM1            CGGCTTAGTCCTCAGCCCCACGATATGAATGAGATAGTTCAGCGCTATGCCCAGGTCCTAAATGGTGAATCAAAGAATACGGAGGTTGAATCCTGGCTGA 49300 
GP59            .................................................................................................... 49300  => protease

JAM1            ACGCTCCTGCATGTGACGGTTATTGGAGTGGTCAATCCGGGATGGACTATATTGCAGTTGTTGCTGAATAAAGAACCTAAGCTCCGGGGTGTTTATTCTG 49400 
GP59            .................................................................................................... 49400 

JAM1            ATGAATAACAGGATGTGGTTGATTAGGTTTTTTCTTGCGCCTTGGTCCGTGGTCGCAAGAAATCAGCCGAAGTATTTATAGCTTACCCTCAGTTCCCTTC 49500 <= cytochrome; HP
GP59            .................................................................................................... 49500

JAM1            ACGTAGCTGTGTGACTTCATCAACAGTGACTAATTCACCTTGATTGCCCCAGGCATTCATTACATAGGTCATGATATGGGCGATCTCATTATCTTTGAGA 49600 483583
GP59            .................................................................................................... 49600 

JAM1            CTGGTGAAAGCAGGCATGACACTATTGTACTTTTCGCCATTAACGATGATTTCTCCTTGTAAACCATTGAGGATAACGCCAATAGCGCGTTGTTTGTCTG 49700 
GP59            .................................................................................................... 49700 

JAM1            CCATCAGATAATCGGAGCCTGCGAGCGGTGGGAAAACACCTTTCATTCCTTGGCCATTAGCTTGATGACACGCAAGACAGTTACTTTCATAGAGCTTTTT 49800 fnr
GP59            .................................................................................................... 49800 

JAM1            GCCTTCAATAAAACTTTCTGAATTTTCGATGGCGAAACTTTGTGAAGAGATACAGAACGATATGGATGTTATCGTCAGGCTCAAGAAGGGTTTGATTTTC 49900 
GP59            .................................................................................................... 49900 

JAM1            ATTTCTTACTCCGCTATTCGAGATAAATAGTTTCACTGTTAAAACCTAAAAGCACGTTTGTGGCATTAAGCTTGAAGACTATAATATAGCGGGTTAACAA 50000 
GP59            .................................................................................................... 50000 

JAM1            GTATGCTTGATCTGGATCAATAGTCGGTAATATAGCAAACACAACTCTCTTTTATTTTCAATAGTTCATCCCGTATTTTGTATACTGAAGCAGCGTAATG 50100 
GP59            .................................................................................................... 50100 

JAM1            TAGCTATCTAAAGACATAAGTCATTATACGGTGCTGGATGATTCTCCCTTTTTACCAAATCAGACGCTCTTCAGTGTCTTTTGTTGATCACTGTTATGTC 50200 <= HP
GP59            .................................................................................................... 50200 

JAM1            GGGCATTGGCGTTTCAGCCTGTGTGATGATGCACTGCCAGACATTAATCCGGAGTATGATGACAAAAATCCTTGCTGACAAAATGATTAGCTTAAAACGG 50300 
GP59            .................................................................................................... 50300 

JAM1            CCTGCCAGCAGGTTTTTTGTGTCGTTATCTACGTCAAAATATGAAAAGTTACGGATCAGCATGGCATAGACTGTTATCCTGATTTGGTTTTTTCTTTTCA 50400 
GP59            .................................................................................................... 50400 

JAM1            ATATAGGCCAGACCAATACTGAGTCGTCCACGAACAGATAAACAGACCTGAGCCAGTTTCCACTTTGAATGAGGAAATACTATTACTATGAGACCAAAAA 50500 => HP
GP59            .................................................................................................... 50500 

JAM1            GTTTATTAAAGTTGGAGTGGGATGCCATTGCGGGGATTATTGCCGCTATCGTTGCTATAGTCTTACATTTTCTACACATCGCTCAAGCTGACTTGTTATT 50600 484583
GP59            .................................................................................................... 50600 

JAM1            GCCAATTTTGCTTGCTCTTATTGGGCTTTTGTTTATTAATTTCCTGCGACATTCAAAAAACAATGAAATAACGGCGGAAACAATAGAAAAGATCCATAAG 50700 
GP59            .................................................................................................... 50700 

JAM1            CAGATTGATTTGGTGCAGAATTCGCTCAAAGAATCAGATATAGAACTCATCGGCCCACGTAAATTACGTTCAGAAAATGACCGTTTTCTAAAAGAAATCA 50800 
GP59            .................................................................................................... 50800 

JAM1            GCGGAGATGTTGTTTTTTATAATATTTGTCTCAAAATGTATTCTGCTGAAGCAATGTTTAATGGGTTGCTGCGACCTGTTTTCGATAATCCTGGTGTTAG 50900 
GP59            .................................................................................................... 50900 

JAM1            CTCCGTCTGTTTCATACTTGATGTTGGACAACAAGCCAACTGGGAAAGAACCATTACCTCGAGACTCTCAGGACATCCACGGCGTGGCATTTTACGCGAA 51000 
GP59            .................................................................................................... 51000 

JAM1            CCAAAATGGTGCTCTTTAGCGGCGAGTTTATCTTTTATTATGGCGGAACAATCTGAAAGTAAATGTGTGGAAGCGTTATTGAGCTTTTGGGGTGAGCCGT 51100 485083
GP59            .................................................................................................... 51100 

JAM1            TTATGACGAATCAAACAGGAATTGATACTCCACGTTATATTTTTCGGCTCAAGAAGCATAATGAACTACTGCCACATCTTGAATCATTGATACAAAATCA 51200 => HP
GP59            .................................................................................................... 51200 

JAM1            ACGCTTGCAAAGCAATTAATTTGTTTAATAGAGCCATCCAGTTCGAAATGAGACCCCGACAACTACAAAAAGTATAGAGAGAGTTGTCACGGCGACAATA 51300 <= Cu exp/resistance
GP59            .................................................................................................... 51300 

JAM1            TGCCATGCCATCACTACAAGATTATTTTCAGAAAGGGCAGGAATCACCCGTAATCGTGCATGTGCCGCAATACTAATCGTTAGCAGCAAAAGAATGAGCT 51400 485383
GP59            .................................................................................................... 51400 

JAM1            TTGCCATTATGGGGTGTGTTAATGGATTGGTATAATCAAACCACTGAAAGACATCCGGAATCATGTGATATACAAGCCATAGCCCTGTTATGACTTGTAT 51500 
GP59            .................................................................................................... 51500 

JAM1            AAGCAGTGCTGGCATACCAATTTTTTCATAAACTGACTCAAATTCCAGTAGCCTTGTCGGTGAGCGGTCGCGGAGTACTTTGGGCAAAATGACCACAACA 51600 
GP59            .................................................................................................... 51600 

JAM1            AGGACAATGTGGCCGCCAGTCCAGATGGTCGCACCAAGCACATGCAACAATAGTATTATTGCATCCATAAAATATTCAACCTTATGTTAAGAGGGATGAT 51700 
GP59            .................................................................................................... 51700 

JAM1            TAGCATTTAACACGCGCCTTTTTGCCCATGTAAAAATAGGCTGTGAGGAAGATACAGATGAGATAAAAAAGGATAAAGCCGAAAAAGGCCGCTCTGACAT 51800 <= narK12f
GP59            .................................................................................................... 51800 

JAM1            CTCCGGATATCGACAATGCAGTTCCGTAAGCTTTTGGAATAAAAAATCCACCATATGCAGCAATTGCCGATATAAAACCCAGAATGGCTGCAGATTCACG 51900 
GP59            .................................................................................................... 51900 

JAM1            ATTTGCTTCAATTATTGCTTGTTCTTGCTTATCCTTCCCTAAAGCTTTAGATCGTAGTGTAAGGCAGATCACAGGCACCATCCTAAATGTTGAACCATTC 52000 
GP59            .................................................................................................... 52000 

JAM1            CCTGCTCCACTGGCCAGAAAGAGTAAAAGAAACATCGTGAAAAATCCGCCAAAATGACCCTGGGAGTTAGCGGATGGCAAAAAGGTCATAACCCCAACAA 52100 
GP59            .................................................................................................... 52100 

JAM1            CGCCAACAACCATCACAATAAATAACCAGAACGTGATGACCGCACCACTAAACTTGTCGGCTAATAGCCCCCCTAAAGGCCTTGCTAAAGCGCCAATTAA 52200 
GP59            .................................................................................................... 52200 

JAM1            TGGGCCGATAAATGCATATTTCATGGCATCTATTTCCGGGAAAAGTTTGCCACTCAATAATGGGAATCCTGCTGCAAATCCTATAAAGCTGCCAAAGGTA 52300 
GP59            .................................................................................................... 52300 

JAM1            CCGAGATACAATATACACATTGTCCAGTTATCAAGTCGTTTAAATATAATTGCTTGATCAGAAAATGATGATTTTGTGGAAGAAATATCATTCATACCAA 52400 486383
GP59            .................................................................................................... 52400 

JAM1            ACCATGCAACAATCGCTGCAACGGCAATAAAAGGTACCCAGATCAATGCCGCATTATGCAACCAGATGGGCTTATCAATTTGCGAGCCTTGCATCACAAT 52500 
GP59            .................................................................................................... 52500 

JAM1            TAAAGGCTCCCCACCAAGAAGACTGCTGGAGATAATAATGGGCGCAATCAATTGCATTGCGGAGACCCCTAGATTCCCTAGCCCAGCATTCAGTCCAAGT 52600 
GP59            .................................................................................................... 52600 

JAM1            GCAGTACCTTTCTCCTTTTTAGGAAAGAAAAAACTGATATTGGCCATGCTGGAAGAAAAATTGCCACCGCCAAAACCACACAACAATGCCAGTATCATCA 52700 
GP59            .................................................................................................... 52700 

JAM1            TCGTTATGTAGGGAGTATCAGGATTCTGCAGTGCAAGGCTTAACCAGATACAGGGTATCAAAAGGCTTGCCGTCGACAAAGCTGTCCAGCGCCGCCCACC 52800 
GP59            .................................................................................................... 52800 

JAM1            AAATACAGGTACCATAAAGCCATAAAATAAACGTAAGGTTGCACCTGACAGTGCTGGTAAAGCGGCCAGCCAAAACAACTGGTTGGCACTGTAAGCAAAA 52900 <= narK12f
GP59            .................................................................................................... 52900 

JAM1            CCTAAGTCTGGCATTTTAACGACCAGAATGCTCCAAATGGTCCAGACTGCAAAAGCAAGAAACAGGCAGGGAATTGAGATCCATAAGTTGCGCCTAGCAA 53000 486983
GP59            .................................................................................................... 53000 

JAM1            TGCTTTTACCCGTTTGTTGCCAAAATCTTTGATCTTCTGGTCGCCAATCTTCTAATATAAAAACAGACGGTGTAGAGAGTTCGGGCAGATCAGAAACGCT 53100 487083
GP59            .................................................................................................... 53100 

JAM1            TTGTCCTAAACTCCATTCAATGCGCTCTGCACGACGGATGGTGAAATGCATCCATAGCATTGAGGTGCCCGCCATGAAACACAGCAAAATAAAACTGATT 53200 
GP59            .................................................................................................... 53200 

JAM1            TGCCATACTCCGACAACATCACTAATGAGCCCAAAAGTTAAGGGTAATAAAAAGCCTCCCAGCCCACCGATCATGGCGATCGTACCAACAACAATACCAA 53300 
GP59            .................................................................................................... 53300 

JAM1            TATGATTGGGGAAATAATCTGGAATGTGTTTAAACACGGCCACATTCCCCAGAGAAAGAAAACACCCCAAAACACAAATTAATACTATGAAGCTTACAAT 53400 
GP59            .................................................................................................... 53400 

JAM1            ATTGATTGAAAAAGCAAATTGATAGCTGGCTGTCACGCCATGAACTACGTAGGTTGTCGGTGGGTAGCTGAGTAAAAAAGTACTCACCATAACCATGCCA 53500 
GP59            .................................................................................................... 53500 

JAM1            AAGCTAAGGTACATTGACCGTCTGGCGCCGATCCTGCTGACTAATTTCATAGCGGGCAGTCGCATTAACGATGTCACTACAATAAATATTGCTGTCACTG 53600 
GP59            .................................................................................................... 53600 

JAM1            CTCCCGCTTGGATTAATCCTAAACGATAGACTTCGATGAGGTAAACTGGCAACCATAAAACCAGTGCAATCAAGCCACCGAAAAGCAAAAAGTAGTAAAA 53700 
GP59            .................................................................................................... 53700 

JAM1            TGCAAATCGCCAAACCGCTGGTTCGGTAATGGGTGTGAGTCGTGAAAGTAATTGTTTACGTAACTCGCTACGGACCGGTTCTGGGTGAGCCAAAAGCTGG 53800 
GP59            .................................................................................................... 53800 

JAM1            AAGCCAATCGCAACAACAACCAATGTTAAGGCAGTAAGTTGAATCATTTGCTGCCAGCTCAGATACTGAAGCAGCAGGGCGGTGCCAAAAGATGTCATCG 53900 
GP59            .................................................................................................... 53900 

JAM1            CCATGCCCATCGTAGCAGAACCTAAAATAACTAATGCTTTGGCCTGATCTGACTGGCTAAACCAAGACGATGTGTATCCGGCTCCAACAACAAAACTTCC 54000 
GP59            .................................................................................................... 54000 

JAM1            TCCCAGCAGACCAAAACCGGCAGATGACAAGAGCATCATGCCGTAACTTTCACTTAGACTGAATAACCACAAAAAAGCCGCAGGAATAAGCATCAGTAAA 54100 488083
GP59            .................................................................................................... 54100 

JAM1            GTGAAAACCCGTCTGCCACCAAGATAATCTGACAGCACACCAAAAACCAGTCTGCAGAGCGCGCCAGTTAGAACAGGCATGGCCATCAAAAGTGCAAGCT 54200 
GP59            .................................................................................................... 54200 

JAM1            GCGTGTTTAAAAAACCCATCTCGTCACGAATTTTGATGCCAACGACAGATAAAACCGTCCAGGCAGCAAAACAAAACATCAGCGAGATACTGACTAACCA 54300 <= narK12f
GP59            .................................................................................................... 54300 

JAM1            CAGGGTACGTTTTTGGGCGAATTCTGTTGGTGGGAAAGATTTACTGATCATGATTCAGCAATCTGTCATCTATTGCATTAAGGGAGAGCCGGAAACGTCA 54400 <= ppiD
GP59            .................................................................................................... 54400 

JAM1            ATTTCAATACCCTCTATCTCGGCCTCAGATAATAGGTACTCTATATATTGTGCATACGCTTTATGTTGTACTCGATTATCGAGATACTCAGAAATTTGCT 54500 fnr
GP59            .................................................................................................... 54500 

JAM1            TGACAACATGCTCATAAGGTAGCAACTCACCATCAATATGCTGATCAACCCAAACCACATGAAAACCGAAACGAGTTTCGACTGGACGAGGTATTAGCCC 54600 
GP59            .................................................................................................... 54600 

JAM1            TAATGGTGCAGCAAATACTTGCCGTTCAAACTCGCTTACGGTTTGTCCACGACTCAACTGCCCCAGCTGACCATCGTGTTTCGCTGAATCACAATGAGAA 54700 
GP59            .................................................................................................... 54700 

JAM1            TACTGTTTTGCCAGATCACCAAAACTTTCGGGATGAAGCGTTATTTTATTTATCAATGATTCAGAAAGATTTAATGCTTCTTCCCTTGCTTGGGGATCTT 54800 
GP59            .................................................................................................... 54800 

JAM1            CGGGGTGTGCAGCAATTAAAATATGCCGAGCAGCTATCAATGGCGAAGTACAGAATTTATCAGGGTTGGCCTGGTAATATTGATAGCATTCATCTTGGCT 54900 488883
GP59            .................................................................................................... 54900 

JAM1            TGCCTGTGGCACTTCAACTTCTATATCAATCAATTCATCTATATAGTCTGTGCCTTGATAGTCCTCTGTACTGATCCCACATGCTATGGCCTGCTGACGC 55000 488983
GP59            .................................................................................................... 55000 

JAM1            ATCACTTCAGCAATAACCAAGCATTGAGCCGCTTTGTGAAATGCAGCATCATGAGACGCTGCCGGATGGTACTGCATTTCAGCTAAGACGGCTGAATCTG 55100 <= ppiD
GP59            .................................................................................................... 55100 

JAM1            AAATCTCAACATTGTTGACTTTTATCATCCCCATTTATCCCTTGACTAATTGCCGCCACGCGTTCTGACTACTTGATAGTTTCTGAATAAGTACTTTACT 55200 <= nar2-I
GP59            .................................................................................................... 55200 

JAM1            GGCACGCTTATCATGTGAACGAGACGGCTAAAAGGAAAGATTGCAAAAATAATAAGACCTAATGTCACATGCATTTTGAAAATCCAATGGGCTTCTGAAA 55300 
GP59            .................................................................................................... 55300 

JAM1            TTATCGGAGCCGCTGTTTGGGGGTTCAAAATCAGAATATTCTGGGCCCATCCCATTAATCTAAGCATCTCGCTGCCATCAAGATGGCCCACTGAAACAAA 55400 
GP59            .................................................................................................... 55400 

JAM1            AATACTCCCCAGTCCAAGCAAAAGCTGACCGAGAAGTAAAAACAGAATGAGAATATCCATGTTAGTACTCGATGCTCTAACAGCTGGGTGCGTCAGTCTT 55500 
GP59            .................................................................................................... 55500 

JAM1            CGATGCATTAAAATTAATAAACCATAAAGACATAGCAAGCCAAATATGCCACCAGCCACAATCGCAACAATCTGCTTGACTCCGGGAGAGATTCCCAATG 55600 
GP59            .................................................................................................... 55600 

JAM1            CATGCCAAACCACATTTGGTGTCAGTAGACCAACAAAGTGACCAATAAATATACCTATGATGCCGATATGAAAAGGGATGCTGCCGCGTCGGAGCTGTTT 55700 
GP59            .................................................................................................... 55700 

JAM1            CTTCTCAAGTAACTGGCTAGAACTTGCCTTCCAGGTATATTGCTCGCGCTCATATCGTATTAACGAGCCCAGAAGCAAAATAGCTAATGCAATATACGGA 55800 <= nar2-I
GP59            .................................................................................................... 55800 

JAM1            AAAACACCAAATAACAAGAAATTTAGGTATGCCATATCTCACCCTCTCATATTTGCTTATATGTTAAGTGTCACGGTGCCTTATTAAAATCCATCCAGGT 55900 <= nar2-J
GP59            .................................................................................................... 55900 

JAM1            AACAGGCACCTCACTGACAACTTTATTATCGGTTGTTTGTGAGTTACAACCATTCTCCATGTCTGGGCCAAAGGTTACGGCCTCTTCTTCCCACTCTTTA 56000 489983
GP59            .................................................................................................... 56000 

JAM1            TCGAGTGCTTCGGCAGTGTCATCACGCTTTTCAGTTGCCAGTTGCTGACGAATTACGTCAAGGTCTACCTCAGTGTCAGCTAATTCCAGTAATAATGAAA 56100 
GP59            .................................................................................................... 56100 

JAM1            ACAATACAGCATAGTCGCTTTCACGTTGTTGCAATCGGCACATCAATAGCGCCAAAATGTGCTCCACTTGTCTTAACCACTCACGCGCATTCTCATCACC 56200 
GP59            .................................................................................................... 56200 

JAM1            TTGCGTGACCAGAAACTCAAGAAATAAAGGAATATAATCCGGCAGCTCATGCTGATTAATATCCAGACCTGCTTCACGATAATTCGACAACAGGTCAACC 56300 
GP59            .................................................................................................... 56300 

JAM1            ATAGCCTGACCCCGATCACGGCTTTCGCCGTGCACATGTTCAAAAAGCCAAAGCCCAAGACTCCTGCCCCGTTCAAACAATTCGTCGTATTGCGCTTGCC 56400 
GP59            .................................................................................................... 56400 

JAM1            AGTTCATTAATTCCATTGCCAAATGCTTTTTGATAAAACGTCGCAATTGGCTTTTAGCTGTCGGGCTCAGGTTGCTGTTGTCAATCAGTTCATGAAAGAG 56500 
GP59            .................................................................................................... 56500 <= nar2-J

JAM1            TACTTCTTCCTGTGACAGACTCGTGGTTGGATAGTCCAGTAAGCGAGAAAATACAAATAAGACGTCCATAATTATTTACTGATTGTGATGGGAATGACTT 56600 <= nar2-H
GP59            .................................................................................................... 56600 

JAM1            TGACTTTCCGTTGTGTCGTCTTGGTCTGTCCAAACAAATTAGCCTCACTGTTGCCATCACTACAGCCATTACCAAAACTGAAACCACAACTACTTTTCAT 56700 
GP59            .................................................................................................... 56700 

JAM1            GTCATAGGCATTTTCAGCATAGGCAGCATGGCTCGTGGGAATAACAAAACGATCTTCATAATTAGCAATAGCCATATACCGATACATCTCTTCAATTTGT 56800 490783
GP59            .................................................................................................... 56800 

JAM1            TCAGCCGTTATTCCTACTTGCTCAGGTATGGAATGGTTTTCGACACCATCCACTTGCTGTTCACGTTTGTAGGCCCTCATGGCAATCATACGTTTTAATG 56900 <= nar2-H
GP59            .................................................................................................... 56900 

JAM1            CACGAATAACCGGTTTTTCATCTCCGGCAGTCAGCAGGTTGGCAAGGTATCGTACAGGTATCCTTAGTGACTCAAGCTCTGGAATCACTCCATTCATCGA 57000 490983
GP59            .................................................................................................... 57000 

JAM1            TACATGGCCAGCTTCCGCAGCAGATTGGATTGGAGATAATGGCGGTATATACCAAACCATAGGCAACGTCCGGTATTCCGGATGAAGAGGTAAAGCGACC 57100 
GP59            .................................................................................................... 57100 

JAM1            TGCCAATCAATCGCCATTTTATATACGGGTGACTGGCGAGCAGCTTCGAGCCAACTGTCAGGAATATTTTCTTTTTGAGCAGCAGCAATCACTTCTGGAT 57200 
GP59            .................................................................................................... 57200 

JAM1            CACTGGGATCTAAAAAGATATCAAGCTGTGCCGGATAAAGGTCCTGTTCATCTGCGACAGCTGCCGCTTCTTCTATACGATCGGCGTCATAAAGCAGAAC 57300 
GP59            .................................................................................................... 57300 

JAM1            ACCAAGATAGCGGATACGCCCAACACATGTTTCAGAACAAACGGTAGGTTGACCTGCCTCGATACGTGGATAACAAAAAATACATTTTTCAGATTTGCCA 57400 
GP59            .................................................................................................... 57400 

JAM1            CTTTTCCAGTTGTAATAAATTTTCTTGTAGGGACAACCTGATACACACATGCGCCAGCCACGGCATTTATCCTGATCAATAAGAACTATGCCATCTTCTT 57500 
GP59            .................................................................................................... 57500 

JAM1            CTCTTTTATAAATTGCACCCGAAGGACAGCTTGCTACACAGGTTGGATTAAGACAGTGTTCACAAAGACGAGGCAAATACATCATGAACGTCTGTTCAAA 57600 
GP59            .................................................................................................... 57600 

JAM1            CTGCCCGTATATATCTTTTTGAATTTGATCAAAGTTTTTATCGCGTTTACGTTTTTCAAACTCTGTGCCGAGTATTTCCTCCCAATTCGGGCCCCAGTTA 57700 
GP59            .................................................................................................... 57700 

JAM1            ATTTTTTCCATACGTTTACCACTGATAAGTGATCGTGGTCTGGCTATCGGTTGATGTTTGCTGTCTTTTGCCGTATGTAAATGTTGATAATCAAAATCAA 57800 
GP59            .................................................................................................... 57800 

JAM1            AGGGTTCGTAGTAGTCATTAATTTCAGGTAGATCAGGATTGGCAAAAATATTAGCTAGGATCCGTAATTTACCCCCTTGTTTTGGGACTAACTTGCCATT 57900 
GP59            .................................................................................................... 57900 

JAM1            TTTTTTGCGTGTCCAGCCGCCGTTGTATTTTTCCTGATTTTCCCACTCTTTGGGATAGCCAATGCCGGGTTTGGTTTCCACATTATTGAACCACGCATAT 58000 491983
GP59            .................................................................................................... 58000 

JAM1            TCCATACCCTCACGGCTGGTCCAGACGTTTTTGCAGGTCACGGAACAGGTATGGCAGCCAATACATTTATCGAGGTTAAGCACCATGCCGATTTGTGCAC 58100 <= nar2-H
GP59            .................................................................................................... 58100 

JAM1            GTACTTTCATAATCTCTCCTCCTGCTCATCGTCAAGCCAATCGACTTTGTTCATTTTTCTAACAATGACAAACTCATCACGGTTAGAGCCAACAGTACCG 58200 <= nar2-G
GP59            .................................................................................................... 58200 

JAM1            TAGTAATTGAAACCATAAGAGAACTGCGCATAGCCACCAATCATATGCGTTGGTTTCATCACGATACGAGTCACAGAGTTATGGATTCCACCACGACCCC 58300 
GP59            .................................................................................................... 58300 

JAM1            CCGTTATTTCTGAGCCCGGAATATTTACTATTCTCTCCTGTGCGTGATACATCATGCTCATACCTTGAGGAACACGTTGAGAAACAACAGCACGGCATGT 58400 fnr
GP59            .................................................................................................... 58400 

JAM1            GAGTGCGCCATTCACATTGAAAACTTCAATCCAATCGTTGTCTTCAATACCTGCATTTTTTGCATCAATTTCACTCATCCACACGATTGGGCCACCCCGA 58500 
GP59            .................................................................................................... 58500 

JAM1            CTCAATGTCAGCATTAACAAGTTATCTGAATAGGTTGAGTGAATGCCCCATTTCTGATGCGGGGTTATCCAATTCAGCACAATTTCCTTATGACCATTGG 58600 
GP59            .................................................................................................... 58600 

JAM1            GATTGATGTTAAGAATCGGAGTTGTCGTTTTGAGGTTCATCGGTGGCTTGTACAAAACGAATTGTTCACCAAAATCACGCATCCATGGATGATCCTGATA 58700 492683
GP59            .................................................................................................... 58700 

JAM1            AAATTGCTGTCGACCAGTCAAAGTACGCCAGGGGATTAGTTCATGTACATTGGTATAGCCAGCATTGTAGGAAACATGTTCATCTTCAAGTCCAGACCAG 58800 <= nar2-G
GP59            .................................................................................................... 58800 

JAM1            GTAGGCGAACTGATGATCTTACGAGGCTGCGCCTGAATATCCCGAAACCGGATCTTTTCCTCTGCTTTGTTTTTGGCAAGGTGGGTGTGATCAATTCCGG 58900 492883
GP59            .................................................................................................... 58900 

JAM1            TGAACTCAGATAATGCTGCCCAAGCTTTTACTGCCACCTGGCCGTTGGTTTCGGGAGCCAGGCCTAAAATGATTTCGGTAGCATCGATTGCACTCTCGAT 59000 
GP59            .................................................................................................... 59000 

JAM1            TCTTGGCCGCCCTTTGTTGATTCCTGGTGCCTGGTGAACATAATTGAGCTTACCGAGAAAGTTAACTTCATCTTTGGTGTCCCAACTGATGCCTTTGCCG 59100 
GP59            .................................................................................................... 59100 

JAM1            CCATTACCCAACTTTTCAAGCAGTGGTCCAAGTGAAACAAAACGCTGGTAGGTGTTTGGATAATCGCGTTCAACAACAACCATACTCGGTGCCGTTACTC 59200 
GP59            .................................................................................................... 59200 

JAM1            CGGGTATTAGTTCACATTCATTCTTCCACCACATCTTGACACCGTCGGCCTGTGCAATTTCAGCAGGTGTATCATGTTGCAGCGGCTGAGTAACCAAATC 59300 
GP59            .................................................................................................... 59300 

JAM1            TTTCTCAACGCCTAAATGTCCAACACACGCTGCTGAAAAAGCCTTCGCAATGCCTTTAAAAATATCCCAGTCACTTTTTGATTGCCATACCGGATCCACG 59400 
GP59            .................................................................................................... 59400 

JAM1            GCTTTAGATAATGGGTGTATAAAGGGATGCATATCTGAGGTATTGAGGTCATCTTTTTCGTACCAGGTTGCTGTTGGCAGCACAATATCAGAATACAAAC 59500 
GP59            .................................................................................................... 59500 

JAM1            ATGTCGTAGACATACGAAAATCAAGTGTTACCAACAGATCAACTTTTCCCTCAACAGCTTCATCCTGCCAATGAACATCTACTGGTTTAGCTTGACCAGA 59600 
GP59            .................................................................................................... 59600 

JAM1            TTCACCGAGATCTTTGCCCTGTAGACCATGGCGCGTACCCAGCAGGTGTCGCAACATGTATTCATGACCTTTACCCGAAGACCCCAACAGGTTGGATCGC 59700 
GP59            .................................................................................................... 59700 

JAM1            CAAATAAATAAATTCCGAGGAAAGTTCTCAGGACTGTCTGGTTCAAGACAAGCAAAATTGAGCTTGCCTGTACGCAATTGGTCAACCACATAATCCTGGG 59800 
GP59            .................................................................................................... 59800 

JAM1            GATCTCTTCCTGCTGCTTCCGCCTCACGGGTAAGTTTCAGGGGGTTGCGACCTAATTGAGGTGCCGATGGTAACCACCCCATTCTCTCGGCACGGGTATT 59900 493883
GP59            .................................................................................................... 59900 

JAM1            GAAATCAATTATCGACCCTTGCCAGCGGGACTCATCTGCCAAAGGCGACAACATTTCATTGATAGACAGTTTTTCATAACGCCACTGATTCGAGTGAGTA 60000 
GP59            .................................................................................................... 60000 

JAM1            TAAAAGAATGAAGTGCCGTTCATATGGCGTGGTGGACGGCTCCAATCAAGCCCAAATGCGAGAGGTAGCCAGCCTGTTTGTGGTCGTAGCTTTTCCTGCC 60100 <= nar2-G
GP59            .................................................................................................... 60100 

JAM1            CAACATAATGTGCCCAACCTCCGCCAGATTGACCAACACACCCGCATAAAATAAGCATATTGATAAGGCCACGATAGTTCATATCCATGTTGAACCAATG 60200 
GP59            .................................................................................................... 60200 

JAM1            GTTCAAACCCGCACCAACAATAATCATGCTTCGACCGTTGGTTTTGGCAGCAGTCTCTGCAAATTCACGCGCAATTTGAACCACACGATCGGCAGGAACT 60300 
GP59            .................................................................................................... 60300 

JAM1            CCCGTAATCTGTTGCTGCCAGGCTGGCGTGTACGGAATATTATCCTCATAGCTGCGAGCCGTATTCTGATCATTGAGCTCATTGTCGACACCATAATGTG 60400 
GP59            .................................................................................................... 60400 

JAM1            CCAGCATCAGATCAAATACCGTAGCAACCTGGAGAGTGCTGCCATCCTTTAACGTGATATTTTTAACCGGTACTTTTCGCTGTTGGAGCTCTTTATGATC 60500 
GP59            .................................................................................................... 60500 

JAM1            GGTATGCTGAAAATAACCGAGATCGTGTACCTTTGATCCGAAATATGGAAACAGCACTTCGGCGACATCATCAGCTGATTCTTTCTGGCTCAATAATAGC 60600 494583
GP59            .................................................................................................... 60600 

JAM1            TGACATGGCTTACTATCAGCGCCAAAATTTTGCTGCAGGTTCCATTTTCCTTTCTCCCCCCAGCGATAACCGATACTGCCATTTGGTGATACCAGCTGAG 60700 <= nar2-G
GP59            .................................................................................................... 60700 

JAM1            CCGTTTGTTCATCAATACCTACCATTTTCCATTGCGGATTATTCTCCTCGCCTAAGTTATCTATCAGATCGCTGGCGCGTAAGAATGATCCTTGAACGAA 60800 494783
GP59            .................................................................................................... 60800 

JAM1            GCTTCCCGAGTCGTGAGGTTCCAGCTTTACTAATAAAGGTAAGTCGGTATAACGACGCGCATAATCGATAAAGTAATCGACCTGTTTATCTACATAAAAC 60900 
GP59            .................................................................................................... 60900 

JAM1            TCTTTAAGAATGACATGGCCAAAAGCCATCGCCAGGGCCGCATCAGTACCTTGTTTTGGAGATAGCCAAATATCGGATAACTTCGCACATTCTGAGTAAT 61000 
GP59            .................................................................................................... 61000 

JAM1            CTGAAGCAATAGCAACTGTTTTCGTGCCTTTGTAACGGACCTCAGTGAAAAAATGTGCGTCAGGAGTACGCGTTTGCGGAACATTAGAACCCCAGGCGAT 61100 
GP59            .................................................................................................... 61100 

JAM1            TAGGTAAGCTGAATTGAACCAGTCAGCTGACTCGGGTACATCGGTCTGTTCACCCCAAGTCTGAGGTGATGAGGGTGGTAAATCGCAATACCAATCATAA 61200 
GP59            .................................................................................................... 61200 

JAM1            AAGCTAAGACAGTTCGCGCCAATTAGAGAAAGATAACGTGATCCTGCAGCGTAACTGACCATACTCATCGCTGGAATAGGTGAAAACCCCGAAATTCGAT 61300 
GP59            .................................................................................................... 61300 

JAM1            CTGGACCGAAAGTTTTTGCGGTGTAAACATTGCTCGCAGCAATAATTTCGTTGACTTCATCCCAATCAGCTCGAACAAAGCCACCCATACCTCGGCGAGT 61400 
GP59            .................................................................................................... 61400 

JAM1            TTTGTAGCTGTTCGTTTTTGTACTGTCCTCTACGATAGATTGCCAGGCATCGACTGGATCGGCATGAATCTGCTTGGCTTCTCTCCATAAAGACAAGAGT 61500 
GP59            .................................................................................................... 61500 

JAM1            GTCTGCCGTACTTTTGGGTATTTGAGCCGATTGGCACTGTATATGTACCAGGAGAAGCTTGCGCCACGAGGGCATCCTCGCGGTTCATGATTAGGTAGAT 61600 
GP59            .................................................................................................... 61600 

JAM1            CTGGGCGGGTACGAGGGTAGTCCGTTTGCTGGGTTTCCCATGTTATCAAGCCATCTTTAACATAAATTTTCCAACTGCATGATCCAGTACAGTTCACTCC 61700 495683
GP59            .................................................................................................... 61700 

JAM1            ATGGGTTGATCTGACAATCTTGTCATGTTGCCAACGCTGCCTGTAACCTTTTTCCCATTCACGGTTCTCATCAGTTGTAACACCATGATCCGCTGAAAAA 61800 <= nar2-G
GP59            .................................................................................................... 61800 

JAM1            GTTTCTGACCGCTGTTTAAAAAAACGAAGTTTATCTAGCAAGTGACTCATGTCTTGGTCTCCTACAGTTTTTAGCGTTTGATGTAACGGTAACACACAAA 61900 Intergenic
GP59            ..................................................TA................................................ 61900 
                                                            nar2<=

JAM1            TTAGAGTAACTTCATAACAAAAAGTGAAACGCATATAACGAACACTGCTAAGGCATGGTTTCTGGCAACAAAACGTAAAAACTGTAATGCTGTCACTTAT 62000 
GP59            .....................................................T.........................TG.........C......... 62000 

JAM1            TCAGGGCTGGTTTAACTTTCTTCTCGGGCGCTGTTGTGAACTAAAAAACGCCATTCTGCCAAGTCTACGAGGAGGTCAGAAGGCTGAGTAGAATGCGGAA 62100 
GP59            .....A.......T......A.T...................GG..T...............A....T..A............................. 62100 

JAM1            GCTTTGTTCGTCGCTTGGTTAAAATGTCTGCGAAAATGCCGGAAAGCCAGGTTTCCGACATAAATTTTGTGTAAACACGTTTGATGAAACAACACCAAGG 62200 
GP59            ............A....................................................................................... 62200 

JAM1            TCACTTAAGCACTCACCAGTCGCCTCATCCAATCTAACAAGGCTGTCGATAATAATTCGGGTTTGGTCAAGAGCGCATATCCACCAGACCCAAAAATACG 62300 <= nor2D
GP59            ..................A....T......................................C..................................... 62300 
                    <= end nor2D (strand -)


JAM1            AGGAAGGTAGTTCGCCGCCTGACGATCGATGGTCAAACAGAAGCAATGAATGCCTTGCAACTTTGATTCAGTGACTGATTGACGCATATCCTCTACACCA 62400 496383
GP59            .............................................................C...................................... 62400 


JAM1            TAACGACCTTCATAATCGTCTTTGTCACTAGGTTTACCATCGGAAATCAATAATAGCAGTCTGTGAGCTGCAGGCTGATGCATAAGGCCTGATGTCGCAT 62500 496483
GP59            ..................................................................A................................. 62500 

JAM1            GACGTATGGCGGCTCCAGTACGAGTATAGTGCTGTGGCTCTAAGGCACTAATTTTAAGGGCAATATCATTGTTATAGCGCTCATCAAAGTCTTTGATTTG 62600 
GP59            ....A............................................G.................................................. 62600 

JAM1            TCGAATAGTAACTGCATTGGGGCCTTCACCGGAAAAGGCCTGAACGGAATATGGTTCGCCCAGGCCCTCAAGAGCAACGCAAACCAGAAGAAGCGCTTCT 62700 <= nor2D
GP59            .................................................................................................... 62700 

JAM1            CTTTCTACATCAATTACTCGTCGGTTCGCTGAAACCCAACTGTCGGTGGAGCCACTGATGTCAATAAGGAGAGTGATAGCCATGTTACGATCAGTATAAC 62800 
GP59            .................................................................................................... 62800 

JAM1            GTCGTGTTTGGTACAAAGCCTCGGCCAAATATTTACCGGCACGAAAATCCGCATAACTGTCTGTGTAAGCATTAAGATCAATATCATCCCCGTCAAGCTG 62900 
GP59            .................................................................................................... 62900 

JAM1            TTTACGACGCGTTACTCGGCGTGCCTGTAGCATTTCGAAGCGTTTGCGTATTGACTGAAGCATGGATTCATGGGCGGCCAGGGTATTGTCGACCCAGTTT 63000 
GP59            .................................................................................................... 63000 

JAM1            TGTGAGCCCTCCTGAGAAGAAAGTACTCGAATAGTCGCTCCTGGTTCACGGTAAATTTGCTCGCGATAGTCCCATTCTGGATAGCTGAAACCTTTACCTT 63100 
GP59            ......................................................T............................................. 63100 

JAM1            CAACTATTGCCGCTTTAAGTTCCTTTCGTGCACGAGCATCTGGAGGATCGTCTGATAGAAGCACTTCCTTTGGCCGACCTGGTGTAGAAACCAGGCGTGC 63200 
GP59            .................................................................................................... 63200 

JAM1            ATTAGCGAGCTCGGAAACCATTTCACCAAATTCATCGGCGCTGGTGTCCCGATCTCGATCTGTTGGTCGTTGAAGTCCAAAGGGATCTTCTGCATGTGGA 63300 
GP59            .................................................T.................................................. 63300 

JAM1            TGTGACTCATCGGCCTGAACCATCCAGATCCCCGATTCTGGGTTCTCGTTTTGTTCCTCTTTTGTGGCTTCGCGTTCATCGGGGCGGCGGGAAAGCCTTG 63400 
GP59            .................................................................................................... 63400 

JAM1            CTGATCTCGGCGTACTGTTTAGATCTTCGCCATTATCTTGTTCCATTGCTGTCGGAATAACGTCATGTTGTGGGGAGGGAGGCCGGAGTTCACCTGTCCA 63500 497483
GP59            .................................................................................................... 63500 

JAM1            CCAATCCTTCACTAATGGAGAGGGACCAAGATAACGTTCATCAACACCGGTTGGCAATAGTTGAAAAGTTCGTATAACTTCCGAGACATTGTTCAAAGAA 63600 
GP59            .................................................................................................... 63600 

JAM1            CCATCTGGTGTGATGGTAAGAGGAATATCCTTATCTGGATGACCACATTGCGTTTTTAAAAGTCTACGCAGGAATAGTTCGAGCGGTTTCCGCGCATCAG 63700 
GP59            .................................................................................................... 63700 

JAM1            AAAACGTTGCTAAAGCGGGTCTTTTTTTAAGAGCTTTCCCGCGTAACTTGTTTACTGTTTCCGTGATGCCGGGTAATAGCGTTACCAATTCCTCGTCGGC 63800 
GP59            .................................................................................................... 63800 

JAM1            GGCATAGGCCTCAAGCAAAAGATAGACATCATTTTGCAAAGCGCTCCTCTCAATATTTGCATATGCTGCTCCACCCCGATGAGCTCTAATCGCCTGTTGA 63900 
GP59            .................................................................................................... 63900 

JAM1            AGGACCATAGCACGATAAATTTCTGTAGCTTGGGTTGAATCTGCGATTTCTAAGTCCGGTGGTAACCAGATAGATGAGCCATTAGTTGCGGGTATTGGAT 64000 
GP59            .................................................................................................... 64000 

JAM1            CTTGTCGACGGGGCTCTCGGTCATATCGAAATAGCATCGCGAGCAAGGTGGCCCGTGATGGCCGTTGCGCTGAACGTAAATTATAACTGCTGCCGAATAT 64100 
GP59            .................................................................................................... 64100 

JAM1            CGCTGTTATCAACAGATCCAGACGTGCTGCAACATCGCACAGAGTAACCGCTTGTCTTTGCTCTGGTGGTGTTCTATGTTTGCGCCAAAACTTTTGTACA 64200 <= nor2D
GP59            .................................................................................................... 64200 

JAM1            AATATCGTCGCATGGCGAGCAGCATCAGAGATGAGTTCTTCAGCTTCTGCCATTGAGCTCCTCACTCATTATCAACGAGTCAGCTTTATACAAAAGTGGC 64300 <= nor2Q
GP59            .................................................................................................... 64300 

JAM1            GTCAACCAGATCACGCATGGCACCGACCAGTGATTCATCATCAGACAAAGGTGAAATTAAACCGGCGTAGCAGGCTTCACGAATTGGAATGCCTTTTTCG 64400 <= nor2Q
GP59            .................................................................................................... 64400 

JAM1            ACTAACAAAGCAGTAGCTATCAGCAACCGAGTGCTGGGTACTTCTGCAAGCCCACGGTCTTTAAGGGCACGAATTCTGTGCGCCAGTGATACCAGGGCAT 64500 498483
GP59            .................................................................................................... 64500 

JAM1            GCGCCGTCGAATAATCTGCGCCACTTTCTTCGCGAATAATGGTGAGTTCATGCCTGGCAGACGGAAAGTCCAGATCCATGGCGACAAAACGCTGGCGGGT 64600 
GP59            .................................................................................................... 64600 

JAM1            GGAAGGTTTAAGATCTTTTAGCATCCGTTGATAGCCAGGGTTGTAGGAAACCACCAGCTGAAATCCTGTCGCTGCTTTGACGGTTTCACCCGTTTTATCA 64700 
GP59            .................................................................................................... 64700 

JAM1            ATCGGAAGAATACGTCTGTGATCTGTTAATGCATGTAACACAACGATAGTATCCTGTCGGGCCTCAACGATTTCATCGAGATAGCAGATTGCACCTTCAC 64800 
GP59            .................................................................................................... 64800 

JAM1            GTACCGCCTGAGTTAGTGGCCCATCGTACCATTGTGTTCCATCGTGCCGAATCAGAAACCGGCCAATAAGATCACTGGCGGCTAAATCGTCATGACAGGG 64900 
GP59            .................................................................................................... 64900 

JAM1            AATGGTAATCAGTGGTCGACCAAGCCGCCAGGCCATATGTTCAACCAAACGGGTTTTACCACAACCCGTCGGGCCTTTAAGCATGACGGCAAGATTTTGT 65000 
GP59            .................................................................................................... 65000 

JAM1            GCATGACATTGCGCAAAAATGTCTACTTCATTGCCCGATGGCAAATAGAAAGGTTCAGCTTTAACTTCAGGCACAGAGCGTTTATGCAGAAATTCAGCTG 65100 <= nor2Q
GP59            .................................................................................................... 65100 

JAM1            TCACCGTTCTACAGTTTCTTTGTTAACCGGATCTTCTTCATGTGATGTTTCAAAATGAGGCCGGTACCTGAAAAAGTCGAATATGAATAATGCAACTCCA 65200 <= nor2B
GP59            .................................................................................................... 65200 

JAM1            ATAGTAAAAATTGATGCCGTTGCAATTAGCATCAGAAAATGAACCTGAATTTTAAGTTGGGCATCCAAATATCCCATGCCCATGATCCGTTCCAGATAAA 65300 
GP59            .................................................................................................... 65300 

JAM1            CCTGCCCGAGGCCTGCAGTAGCAAAAGATAAAGTCATACCAAACATACCCGCAAGCTGTAGCCAGAAAGCCCAATAGCCCAATGTGGAACCCTCTTCTGA 65400 
GP59            .................................................................................................... 65400 

JAM1            ACGATTAGTCATCTTCGGTAGAATATACGTTATCATTGCTAGCACAATCATGGCGTAGGCACCATAAAAAGCCGCATGACCATGCATCGCCGTAATTAGC 65500 499483
GP59            .................................................................................................... 65500 

JAM1            GTCCCATGCGTCCATTTATTGACTGCTGGCCAAGTGTGTGCAAGACCCAACAATCCCGCACCAAAAAGCGCAAATACTGTGCTGCCAATGGTCCAGTGTA 65600 
GP59            .................................................................................................... 65600 

JAM1            ATGCTAGCGTATTGGGGTGGGCCAAACCTGAGCGGCGCATGGCTGCATATGCAAACGCGGCCATGCCAACTAGTGCTGCTGGTTCAAGAGCACTAAAAAA 65700 
GP59            .................................................................................................... 65700 

JAM1            ACCACCGATCGGAAGCCAATACTGAGGCACTCCTACCCAGTAGTAATGATGCGCAGTTCCGAGAATTCCCGCGATAAACACTAATCCGACAATGACATAA 65800 
GP59            .................................................................................................... 65800 

JAM1            AGCCATTTTTCCATGACCTCCCGATCCGCTCCGGACAGACGAATCAGGAGATAGGCAAGAAATGACCCCATAATCATCATCCAGACGCCTTCCACCCAAA 65900 
GP59            .................................................................................................... 65900 

JAM1            GATGAATTGTCCACCACCGATAGAAAATGGAAATCGTATAATTTTCGTAATGAAGTAATGCTGGGAAAAATAACAATGCAGAGCTACCCAACCCTAGTAA 66000 
GP59            .................................................................................................... 66000 

JAM1            TAAGACCCCTTCGGTGGTGGTAAAGCGACCAGACTTTTTAATTGTCATAGCGATGTTATAAAGAAAAATCAACATACAGATTACGATGATGATTTTGTGC 66100 
GP59            .................................................................................................... 66100 

JAM1            GGCAAGGGTTGCTCCAATAGCTTGTTGCCTGTGCCATAACGAAAAAGATAGCCAATGATGGCGGCCACACCCATACCCGTCCAGAGAATCAGTTGTAGGT 66200 
GP59            .................................................................................................... 66200 <= nor2B

JAM1            AAGCAAGCTTGACGCTATGTAGTTCAGTACGCGACTCATCGGGAACCATCCAATAGGTCCCCCCCATAAAGCCTGTTAATACCCATACAATAAGAAGGTT 66300 500283
GP59            .................................................................................................... 66300 

JAM1            GGTGTGTATGACCTTGGTCACGTCGAACGGCAGAATGTAAAGCAGAGGATCCGGACCCAAATACTTGGTGGCAGATAAAAGACCGAATACCAGCTGTAAT 66400 
GP59            .................................................................................................... 66400 <= nor2B

JAM1            CCAAACAAGACCATAGCAACAGCAAAGTACCAATACGCAACTGATTGCGATTTATACCGCATCGTTATTCTCCTTGAGGTTTCTGTCGTCTTGCATCACA 66500 
GP59            .................................................................................................... 66500 

JAM1            CAAGCTAATCGGTCGTGAAACATCATTTCAGTGTTGCCAGAAAGGCTGTTAATTGCTCGAGTTCTTCCTTTGAAAGGCTCTGTTCATAACCTGTCGGCAT 66600 <= nor2C
GP59            .................................................................................................... 66600 

JAM1            GAATGATGTTTCACCGGAAGAATACATTTCTCCGGGCACCAAATAAGCACTAGGCGCGATAATGGATTCTCGTATGTACTCTTCAATATTGGTAGCTTCT 66700 
GP59            .................................................................................................... 66700 

JAM1            CCGTCGTATTCCGGTGATTCAATAATTTCAGCCGCCCGCTTTGCTAAGCCAGCCATGGACGGGCCAGCCATATTGACGTCAGGTTCTGTGGAGTGACATG 66800 
GP59            .................................................................................................... 66800 

JAM1            AGGCACAAGCTGGCTGAGCTGTTCGGAAAATGTGCTCCCCTATTGCACGTGGATCGTCATTCCCGTCTACTGGAGTAACTCCCTGGGGGGCTGCATCCGC 66900 
GP59            .................................................................................................... 66900 

JAM1            TATTACACCACCAGAAACACCGCTTCCCGTGACGGTTATGGGACGAGGAGGCCATCCTTGATTATCTACATTGGAAACCCACTCGAGAAAAGTAATCAGA 67000 
GP59            .................................................................................................... 67000 

JAM1            TCGGCAATCTCCTTTTCGCTCAAATTTTGAGTGGGCATGAGCCGGCGATGCTTTTGCTCATCATAAAATTTTGAAGGGTCTCGCATATAAGCGGTGAGAT 67100 
GP59            .................................................................................................... 67100 

JAM1            AATCTACTGTTCGGTGTTTTGTTATCTTAGTCAGATCAGGTGCGTAATAAGCACCTTCACCAAAAATCGTATGGCAGTTAATGCAATTGTATTTGTGCCA 67200 
GP59            .................................................................................................... 67200 

JAM1            TACGTCTTTCCCGTGGATCACTTCATCGTTGATATTTTCAGCATGCGTCAGATCGTCAATTTGACGGTGAGTATCCAGGGTAAGTACTAGAAATATAAAA 67300 
GP59            .................................................................................................... 67300 

JAM1            GCAGCAATGCCAGTGGATGCAATTGCAAAAATTCGAGCCTGCCGATTAGTCAT 67353 <= nor2C
GP59            ..................................................... 67353


Sequence and gene arrangements aroung nirK in strain GP59 genome (CP021973.1)


GP59        TTTAACCCCGCATTATTTAAAGCCAAATCCCATTATTTCCCTTTAAATTTCGCCAAATCCCATTATTCTTAATCCATGTGTCTTCTTTCAGCGGCGATTA 100  
GP59        TTGAACCATTGAGCGATGAAGAAGAAACCGTTGATGCTTTAATGGAAGTGGTGAAGGCAAAAATACAAATTACAAGTTGAATAATTTAGGAATGAATGAT 200  
GP59        GACTGAGCATGCTGAAAACGATCATACTGCCGCCTTTGCAGAAGCTTTTTATATTGCTAACTTGTTATTTGTCGGTTTCTTTTATCTGGCGTTATGGGCC 300  
GP59        TTGTTTTTTATGCGTTATAAACAAACCTCAAGCATCGCAAAACATCACCTTATTCAATGCTTAGTGGTGGGAACATTGAGTACCTCTATTTTTATTTTGA 400  
GP59        TCAATGGTTATATTATTCTGACAACAGGATATGCTTCTTTAACAGCTCTATTATCATTAGAGCTCTATTTTATGTTGGTTGTGCCGATATTTTTGCTTGC 500  
GP59        TGGAATTATTGGTTTTACGAAAGCCATTAAAGGTTTGGATTATTCTTATCCTTTAGTAGGAAAGGTATTTTAATGGTCGAACATCACTGCTATCACTGTA 600  
GP59        ATGGACCCGTAGAAACTAAAGATGTTAAATGCCCGAATTGTGGTATTCCATTACCTCCAAATCTCGAGAAAAAACGTCAGCATAACTTTATTTTGTGGTT 700  

GP59        TGTTTTCTTGGTCATTTTTTGTTTTTTTATGATGATCTGGTTACCACCCAACTGGACATGATCCATGATTGAAGAATTTGTAGGTGAAACATGGCATCGG 800  norD 3140267
GP59        TTTATAACCAAACATGCAGATAGGCATTATCCTGAAGCATTAGTCGCTTTAGACGATGTTAGACACACGGCGGGAATATTTTTTCGTGCTTTAGGTGGTG 900  
GP59        AAGCAAGCTTAAGAGTTGAAAATGCAACCGACAGTGAAGTGAATTCGCGTCGTTCTTTGGTCGAGCGTATTGCTGGGATCGGAAATAAAACGCAATATGC 1000 
GP59        TTGGCGAGATGAGGATACGTTACGCTTACCCGAGTCGATTGCATTATTTCCAAGTAAACAGCTCAATATTGACTTATATTTATGGCTAACTGCAATTTCA 1100 
GP59        GTTGTCTCCACTGAAGGCGGAGACTGGATTAGTCGAAATCAATATAGAACAAAAAAAACCTTGGAAATATGGCCTGGACTGAAACCACGTTATCAGCAGT 1200 
GP59        TAGTTACTGCTCATTTAACACAGCGCCCGAAATTAACTGACTTGTCTAAAGGTGAAGCTCAACAAGAGAAAACAGTGCAGTCAGCCTTATTAAACTCGAC 1300 
GP59        GCAAGAGTATGTGTTGGAGTATGCAAAACGGCCTCCATACCCAGTTTGTTTATGGTTGCATCCTTCACCACCTAAAATTGAATTTAATCAAAAAAGTCCT 1400 
GP59        CCTAAAGATCCCGACCATCCGGAAAATGTAGAAGCCAAAAAGGAGCAATCTAAGCAGCAAAAACGTAAAAAGGCGAATCGAGAAGACGTTCCTGATGGAA 1500 
GP59        AAAGTGGTTTAATGAGTTTTCGACTTGAAAGCCTTTGGAGTTGGGCCGAATATTCTAAAGTTGATCGAACCAGTACGGAAGATGATGATGATGATGTAGT 1600 
GP59        TAAAACTGCCGAAGATATGGATAGCATCACTATTGGTCAGGATGGTGAAACAACGGCAAGCAAAGTTAAGTTGGATTTAGATTTACCTTCCAGCTCCTAT 1700 
GP59        GATGACATTGTTTTAGGAGAAGGGATTCCCATTGATGAATGGGATTACAAAACACAGAGATTACAAAAAGCACATTGTCGTTTAATTCCAATGTTGTCTC 1800 
GP59        GTGATGCCATTGAAACGACATTACCTGCACGTTTGAACAAGCAAGCTCGAAGAATTCGCCGACAGTTTGAAGTGCTGCGTCCACAACGACATTGGGTTAA 1900 
GP59        TCGTCAAAATGAAGGAACGGAGTTAGATTTAGAAAGCTATGTTAACTTTCTAAGTGATCGAAAACATGGGCATGTACAAACTGATATGCCAGTTTATAAA 2000 
GP59        GATCTACAAAATCAATCACGTGATTTATCCTGTTTACTGCTCGCAGATCTTTCATTGTCGACAGATGCTTATATTAATAATGATTCACGCGTAGTTGATG 2100 
GP59        TGATTCGAGATACCTTATTTCTTTTTTCAGAAGCATTGAATGTGACCGGAGATCGATTTGCCTTACATGGCTTTTCATCTCGTAACCGTAATCATGTCCG 2200 
GP59        TTTTTACGACATTAAACCTTTTGACGAACAATATACGGATATTTCTCGTGGACATATTAATGCGATTCGTCCTGGTTATTACACCCGAATGGGGGCCGCT 2300 
GP59        ATTCGGCATGCTAGTCAATTGCTGGATAAACAAGCGACGAGTCAAAAGCTATTATTGATTTTGACAGACGGCAAACCTAATGACTTAGATAAATACGAAG 2400 
GP59        GGCGTTATGGTTTAGAAGACACTCGGCAAGCAATTATTGAGGCCGAAAGAATGGGCTTGCAACCTTTTTGTGTCACGATTGATGAAAAAGCAGAAGACTA 2500 
GP59        TTTACCTTATTTATTTGGTAAAAATGCGTATCTATTCGTTAAAAATATTGCTGCATTACCCGCAAAATTGCCGCAATTGTATTTACGTTTAACTCAATAA 2600 
                                                                                     
GP59        CCCTAATTATTTTAATAGAAATATCCAGCAAATCCCCTATAACCCTAAAGGTTTTGTGTCAATTGCCTGATTATTGATTTAAATCAATGCTACGGTATCA 2700 fnr
                                                          
GP59        CCGTTGTTAGATAGTGAGCACGGACAACAAAAGAAGATCTAACATTAATAACAACTGTGAGGAAATATTATGAGAACGTTCAATACATGGGGTAAAGGCA 2800 

GP59        TTCTTGCTGCTACTTCTTTATCAATTATGACTTGTTTGTCACCAGCAGTATTTGCTGGTGAAGAAATGGAAGTCGGTAAAGTAGCCGTTGAATCCAATAT 2900 nirK 3142326
GP59        TGCTCATCTAGAAAGAGTGACACAAACATTAGTTGCACCTCCATTTTTACCTGAGCACGATCAAGTTGCTCAAGGTGCGCCTAAAGTTGTTCAAATGACG 3000 
GP59        ATGGAGATTGTTGAAAAAGAAATGGAAATTGCTGAAGATGTGTTTGTACAAGCAATGACGTTTGAAGGTACTAACCCAGGACCTATGATTGTTGTTCATC 3100 
GP59        AAGATGATTATGTTGAATTAACACTTAAAAACCCGGCTACTAGCACAATGGTTCACAACATTGACTTTCATGCTTCAACGGGTGCATTAGGTGGTGGTGG 3200 
GP59        TTTAACCAACGTTGCTCCAGGTGAACAAGTTGTTCTTCGTTTTAAAGCGACAAAAGCAGGTGTGTTTGTTTATCACTGTGCCCCAGGTGGCGCTATGATT 3300 
GP59        CCTTACCATGTTGTTGCTGGTATGAACGGCGCGATTATGGTTTTACCTCGTGATGGTTTAAAAGACAAGAACGGTGACTTAGTCTCTTACGATAAAGCCT 3400 
GP59        ATTACATTGGTGAACAAGATTGGTATATCCCGTTTGATGAAGAAGCGGATGAATATAAACGTTATGATTCACCTGCTTTAGCAATGGGTGAAACCTTTGA 3500 
GP59        AGTCATGAAAACATTAACTCCTACACATCTGACTTTTAACGGTAAAGTGGGCGCGTTAACAGGTGAAAATGCGATGACAGCAAAAGTGGGTGAAACAGTC 3600 
GP59        TTGTTCATTCATTCACAAGCAAATCGTGATTCTCGTGTTCATTTAATTGGTGGTCATGGTGATTTAGTTTGGAATGGTGGTTCATTTGATGATACACCTG 3700 
GP59        CAACCAACCTAGAAACTTGGCCGGTTGCCGCTGGTGAAGCCAGTGCTGCTTTATATACATTCCTACAACCAGGTTTATATGCTTATGTGAATCATAATCT 3800 
GP59        GATTGAAGCGATTTTATTAGGTGCAGCAGCTCACGTTTCAGTAGAAGGTGAGTGGAATAATGACTTAATGGAACAGGTTCAAAAACCTGGCCCAATTCAG 3900 
GP59        TAAGTTTTTAATGAGATTTGACATGCACTCAAGTATTGGGTGCATGTCAGCTTATTTTTATAATGAAAACAATAAAATACATCCCCATACTTTATCTCTT 4000 
GP59        A 4001
