# Supplementary material for: The bacterial strains JAM1T and GP59 of the species Methylophaga nitratireducenticrescens differ in their expression profiles of denitrification genes in oxic and anoxic cultures
Source: PeerJ. 2024 Oct 28;12:e18361. doi: 10.7717/peerj.18361 (PMC11526790; doi:10.7717/peerj.18361)
Supplement: Data S4 [file peerj-12-18361-s010.pptx]

## Slide 1
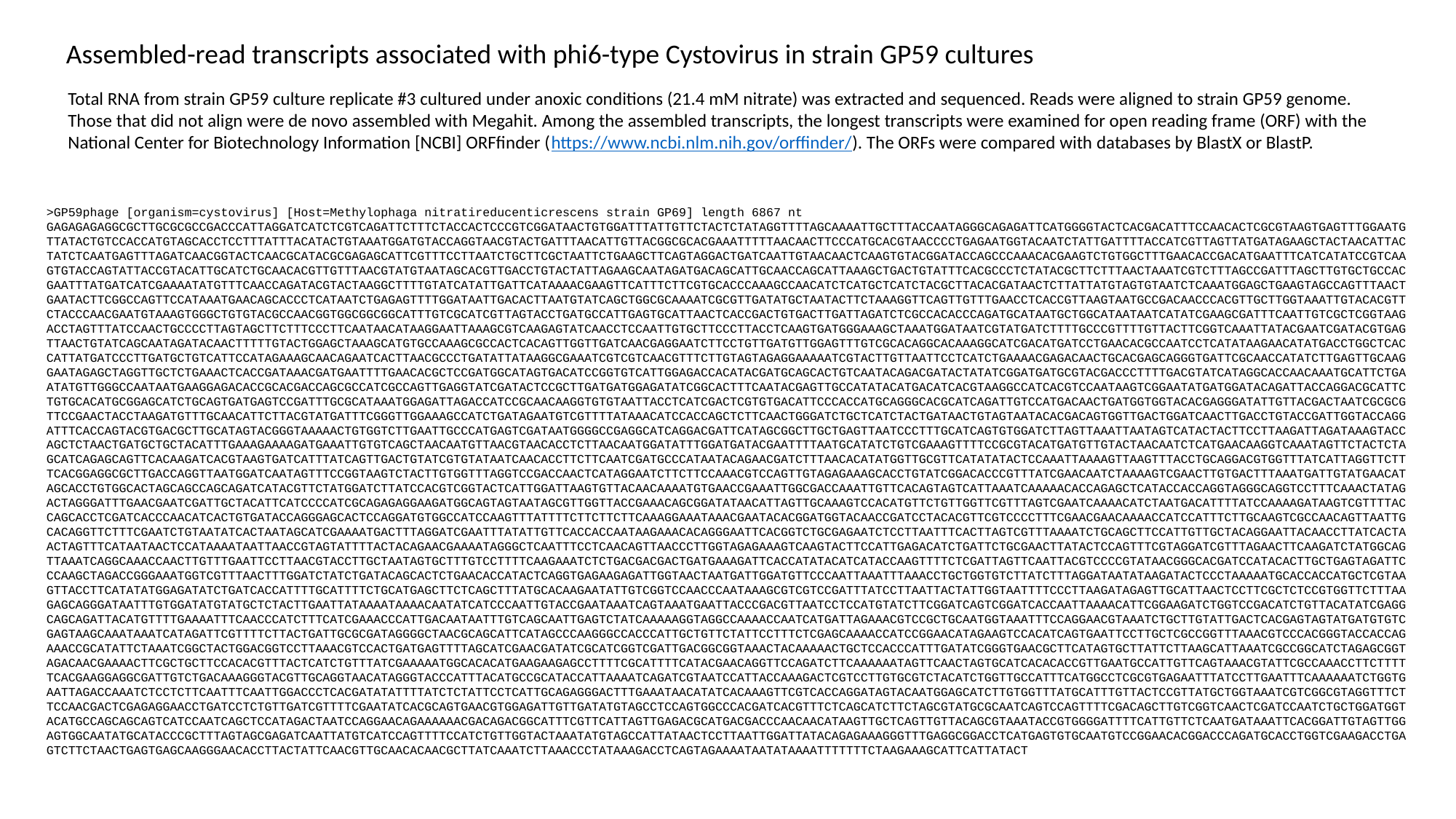

Assembled-read transcripts associated with phi6-type Cystovirus in strain GP59 cultures
Total RNA from strain GP59 culture replicate #3 cultured under anoxic conditions (21.4 mM nitrate) was extracted and sequenced. Reads were aligned to strain GP59 genome. Those that did not align were de novo assembled with Megahit. Among the assembled transcripts, the longest transcripts were examined for open reading frame (ORF) with the National Center for Biotechnology Information [NCBI] ORFfinder (https://www.ncbi.nlm.nih.gov/orffinder/). The ORFs were compared with databases by BlastX or BlastP.
>GP59phage [organism=cystovirus] [Host=Methylophaga nitratireducenticrescens strain GP69] length 6867 nt
GAGAGAGAGGCGCTTGCGCGCCGACCCATTAGGATCATCTCGTCAGATTCTTTCTACCACTCCCGTCGGATAACTGTGGATTTATTGTTCTACTCTATAGGTTTTAGCAAAATTGCTTTACCAATAGGGCAGAGATTCATGGGGTACTCACGACATTTCCAACACTCGCGTAAGTGAGTTTGGAATGTTATACTGTCCACCATGTAGCACCTCCTTTATTTACATACTGTAAATGGATGTACCAGGTAACGTACTGATTTAACATTGTTACGGCGCACGAAATTTTTAACAACTTCCCATGCACGTAACCCCTGAGAATGGTACAATCTATTGATTTTACCATCGTTAGTTATGATAGAAGCTACTAACATTACTATCTCAATGAGTTTAGATCAACGGTACTCAACGCATACGCGAGAGCATTCGTTTCCTTAATCTGCTTCGCTAATTCTGAAGCTTCAGTAGGACTGATCAATTGTAACAACTCAAGTGTACGGATACCAGCCCAAACACGAAGTCTGTGGCTTTGAACACCGACATGAATTTCATCATATCCGTCAAGTGTACCAGTATTACCGTACATTGCATCTGCAACACGTTGTTTAACGTATGTAATAGCACGTTGACCTGTACTATTAGAAGCAATAGATGACAGCATTGCAACCAGCATTAAAGCTGACTGTATTTCACGCCCTCTATACGCTTCTTTAACTAAATCGTCTTTAGCCGATTTAGCTTGTGCTGCCACGAATTTATGATCATCGAAAATATGTTTCAACCAGATACGTACTAAGGCTTTTGTATCATATTGATTCATAAAACGAAGTTCATTTCTTCGTGCACCCAAAGCCAACATCTCATGCTCATCTACGCTTACACGATAACTCTTATTATGTAGTGTAATCTCAAATGGAGCTGAAGTAGCCAGTTTAACTGAATACTTCGGCCAGTTCCATAAATGAACAGCACCCTCATAATCTGAGAGTTTTGGATAATTGACACTTAATGTATCAGCTGGCGCAAAATCGCGTTGATATGCTAATACTTCTAAAGGTTCAGTTGTTTGAACCTCACCGTTAAGTAATGCCGACAACCCACGTTGCTTGGTAAATTGTACACGTTCTACCCAACGAATGTAAAGTGGGCTGTGTACGCCAACGGTGGCGGCGGCATTTGTCGCATCGTTAGTACCTGATGCCATTGAGTGCATTAACTCACCGACTGTGACTTGATTAGATCTCGCCACACCCAGATGCATAATGCTGGCATAATAATCATATCGAAGCGATTTCAATTGTCGCTCGGTAAGACCTAGTTTATCCAACTGCCCCTTAGTAGCTTCTTTCCCTTCAATAACATAAGGAATTAAAGCGTCAAGAGTATCAACCTCCAATTGTGCTTCCCTTACCTCAAGTGATGGGAAAGCTAAATGGATAATCGTATGATCTTTTGCCCGTTTTGTTACTTCGGTCAAATTATACGAATCGATACGTGAGTTAACTGTATCAGCAATAGATACAACTTTTTGTACTGGAGCTAAAGCATGTGCCAAAGCGCCACTCACAGTTGGTTGATCAACGAGGAATCTTCCTGTTGATGTTGGAGTTTGTCGCACAGGCACAAAGGCATCGACATGATCCTGAACACGCCAATCCTCATATAAGAACATATGACCTGGCTCACCATTATGATCCCTTGATGCTGTCATTCCATAGAAAGCAACAGAATCACTTAACGCCCTGATATTATAAGGCGAAATCGTCGTCAACGTTTCTTGTAGTAGAGGAAAAATCGTACTTGTTAATTCCTCATCTGAAAACGAGACAACTGCACGAGCAGGGTGATTCGCAACCATATCTTGAGTTGCAAGGAATAGAGCTAGGTTGCTCTGAAACTCACCGATAAACGATGAATTTTGAACACGCTCCGATGGCATAGTGACATCCGGTGTCATTGGAGACCACATACGATGCAGCACTGTCAATACAGACGATACTATATCGGATGATGCGTACGACCCTTTTGACGTATCATAGGCACCAACAAATGCATTCTGAATATGTTGGGCCAATAATGAAGGAGACACCGCACGACCAGCGCCATCGCCAGTTGAGGTATCGATACTCCGCTTGATGATGGAGATATCGGCACTTTCAATACGAGTTGCCATATACATGACATCACGTAAGGCCATCACGTCCAATAAGTCGGAATATGATGGATACAGATTACCAGGACGCATTCTGTGCACATGCGGAGCATCTGCAGTGATGAGTCCGATTTGCGCATAAATGGAGATTAGACCATCCGCAACAAGGTGTGTAATTACCTCATCGACTCGTGTGACATTCCCACCATGCAGGGCACGCATCAGATTGTCCATGACAACTGATGGTGGTACACGAGGGATATTGTTACGACTAATCGCGCGTTCCGAACTACCTAAGATGTTTGCAACATTCTTACGTATGATTTCGGGTTGGAAAGCCATCTGATAGAATGTCGTTTTATAAACATCCACCAGCTCTTCAACTGGGATCTGCTCATCTACTGATAACTGTAGTAATACACGACAGTGGTTGACTGGATCAACTTGACCTGTACCGATTGGTACCAGGATTTCACCAGTACGTGACGCTTGCATAGTACGGGTAAAAACTGTGGTCTTGAATTGCCCATGAGTCGATAATGGGGCCGAGGCATCAGGACGATTCATAGCGGCTTGCTGAGTTAATCCCTTTGCATCAGTGTGGATCTTAGTTAAATTAATAGTCATACTACTTCCTTAAGATTAGATAAAGTACCAGCTCTAACTGATGCTGCTACATTTGAAAGAAAAGATGAAATTGTGTCAGCTAACAATGTTAACGTAACACCTCTTAACAATGGATATTTGGATGATACGAATTTTAATGCATATCTGTCGAAAGTTTTCCGCGTACATGATGTTGTACTAACAATCTCATGAACAAGGTCAAATAGTTCTACTCTAGCATCAGAGCAGTTCACAAGATCACGTAAGTGATCATTTATCAGTTGACTGTATCGTGTATAATCAACACCTTCTTCAATCGATGCCCATAATACAGAACGATCTTTAACACATATGGTTGCGTTCATATATACTCCAAATTAAAAGTTAAGTTTACCTGCAGGACGTGGTTTATCATTAGGTTCTTTCACGGAGGCGCTTGACCAGGTTAATGGATCAATAGTTTCCGGTAAGTCTACTTGTGGTTTAGGTCCGACCAACTCATAGGAATCTTCTTCCAAACGTCCAGTTGTAGAGAAAGCACCTGTATCGGACACCCGTTTATCGAACAATCTAAAAGTCGAACTTGTGACTTTAAATGATTGTATGAACATAGCACCTGTGGCACTAGCAGCCAGCAGATCATACGTTCTATGGATCTTATCCACGTCGGTACTCATTGGATTAAGTGTTACAACAAAATGTGAACCGAAATTGGCGACCAAATTGTTCACAGTAGTCATTAAATCAAAAACACCAGAGCTCATACCACCAGGTAGGGCAGGTCCTTTCAAACTATAGACTAGGGATTTGAACGAATCGATTGCTACATTCATCCCCATCGCAGAGAGGAAGATGGCAGTAGTAATAGCGTTGGTTACCGAAACAGCGGATATAACATTAGTTGCAAAGTCCACATGTTCTGTTGGTTCGTTTAGTCGAATCAAAACATCTAATGACATTTTATCCAAAAGATAAGTCGTTTTACCAGCACCTCGATCACCCAACATCACTGTGATACCAGGGAGCACTCCAGGATGTGGCCATCCAAGTTTATTTTCTTCTTCTTCAAAGGAAATAAACGAATACACGGATGGTACAACCGATCCTACACGTTCGTCCCCTTTCGAACGAACAAAACCATCCATTTCTTGCAAGTCGCCAACAGTTAATTGCACAGGTTCTTTCGAATCTGTAATATCACTAATAGCATCGAAAATGACTTTAGGATCGAATTTATATTGTTCACCACCAATAAGAAACACAGGGAATTCACGGTCTGCGAGAATCTCCTTAATTTCACTTAGTCGTTTAAAATCTGCAGCTTCCATTGTTGCTACAGGAATTACAACCTTATCACTAACTAGTTTCATAATAACTCCATAAAATAATTAACCGTAGTATTTTACTACAGAACGAAAATAGGGCTCAATTTCCTCAACAGTTAACCCTTGGTAGAGAAAGTCAAGTACTTCCATTGAGACATCTGATTCTGCGAACTTATACTCCAGTTTCGTAGGATCGTTTAGAACTTCAAGATCTATGGCAGTTAAATCAGGCAAACCAACTTGTTTGAATTCCTTAACGTACCTTGCTAATAGTGCTTTGTCCTTTTCAAGAAATCTCTGACGACGACTGATGAAAGATTCACCATATACATCATACCAAGTTTTCTCGATTAGTTCAATTACGTCCCCGTATAACGGGCACGATCCATACACTTGCTGAGTAGATTCCCAAGCTAGACCGGGAAATGGTCGTTTAACTTTGGATCTATCTGATACAGCACTCTGAACACCATACTCAGGTGAGAAGAGATTGGTAACTAATGATTGGATGTTCCCAATTAAATTTAAACCTGCTGGTGTCTTATCTTTAGGATAATATAAGATACTCCCTAAAAATGCACCACCATGCTCGTAAGTTACCTTCATATATGGAGATATCTGATCACCATTTTGCATTTTCTGCATGAGCTTCTCAGCTTTATGCACAAGAATATTGTCGGTCCAACCCAATAAAGCGTCGTCCGATTTATCCTTAATTACTATTGGTAATTTTCCCTTAAGATAGAGTTGCATTAACTCCTTCGCTCTCCGTGGTTCTTTAAGAGCAGGGATAATTTGTGGATATGTATGCTCTACTTGAATTATAAAATAAAACAATATCATCCCAATTGTACCGAATAAATCAGTAAATGAATTACCCGACGTTAATCCTCCATGTATCTTCGGATCAGTCGGATCACCAATTAAAACATTCGGAAGATCTGGTCCGACATCTGTTACATATCGAGGCAGCAGATTACATGTTTTGAAAATTTCAACCCATCTTTCATCGAAACCCATTGACAATAATTTGTCAGCAATTGAGTCTATCAAAAAGGTAGGCCAAAACCAATCATGATTAGAAACGTCCGCTGCAATGGTAAATTTCCAGGAACGTAAATCTGCTTGTATTGACTCACGAGTAGTATGATGTGTCGAGTAAGCAAATAAATCATAGATTCGTTTTCTTACTGATTGCGCGATAGGGGCTAACGCAGCATTCATAGCCCAAGGGCCACCCATTGCTGTTCTATTCCTTTCTCGAGCAAAACCATCCGGAACATAGAAGTCCACATCAGTGAATTCCTTGCTCGCCGGTTTAAACGTCCCACGGGTACCACCAGAAACCGCATATTCTAAATCGGCTACTGGACGGTCCTTAAACGTCCACTGATGAGTTTTAGCATCGAACGATATCGCATCGGTCGATTGACGGCGGTAAACTACAAAAACTGCTCCACCCATTTGATATCGGGTGAACGCTTCATAGTGCTTATTCTTAAGCATTAAATCGCCGGCATCTAGAGCGGTAGACAACGAAAACTTCGCTGCTTCCACACGTTTACTCATCTGTTTATCGAAAAATGGCACACATGAAGAAGAGCCTTTTCGCATTTTCATACGAACAGGTTCCAGATCTTCAAAAAATAGTTCAACTAGTGCATCACACACCGTTGAATGCCATTGTTCAGTAAACGTATTCGCCAAACCTTCTTTTTCACGAAGGAGGCGATTGTCTGACAAAGGGTACGTTGCAGGTAACATAGGGTACCCATTTACATGCCGCATACCATTAAAATCAGATCGTAATCCATTACCAAAGACTCGTCCTTGTGCGTCTACATCTGGTTGCCATTTCATGGCCTCGCGTGAGAATTTATCCTTGAATTTCAAAAAATCTGGTGAATTAGACCAAATCTCCTCTTCAATTTCAATTGGACCCTCACGATATATTTTATCTCTATTCCTCATTGCAGAGGGACTTTGAAATAACATATCACAAAGTTCGTCACCAGGATAGTACAATGGAGCATCTTGTGGTTTATGCATTTGTTACTCCGTTATGCTGGTAAATCGTCGGCGTAGGTTTCTTCCAACGACTCGAGAGGAACCTGATCCTCTGTTGATCGTTTTCGAATATCACGCAGTGAACGTGGAGATTGTTGATATGTAGCCTCCAGTGGCCCACGATCACGTTTCTCAGCATCTTCTAGCGTATGCGCAATCAGTCCAGTTTTCGACAGCTTGTCGGTCAACTCGATCCAATCTGCTGGATGGTACATGCCAGCAGCAGTCATCCAATCAGCTCCATAGACTAATCCAGGAACAGAAAAAACGACAGACGGCATTTCGTTCATTAGTTGAGACGCATGACGACCCAACAACATAAGTTGCTCAGTTGTTACAGCGTAAATACCGTGGGGATTTTCATTGTTCTCAATGATAAATTCACGGATTGTAGTTGGAGTGGCAATATGCATACCCGCTTTAGTAGCGAGATCAATTATGTCATCCAGTTTTCCATCTGTTGGTACTAAATATGTAGCCATTATAACTCCTTAATTGGATTATACAGAGAAAGGGTTTGAGGCGGACCTCATGAGTGTGCAATGTCCGGAACACGGACCCAGATGCACCTGGTCGAAGACCTGAGTCTTCTAACTGAGTGAGCAAGGGAACACCTTACTATTCAACGTTGCAACACAACGCTTATCAAATCTTAAACCCTATAAAGACCTCAGTAGAAAATAATATAAAATTTTTTTCTAAGAAAGCATTCATTATACT

## Slide 2
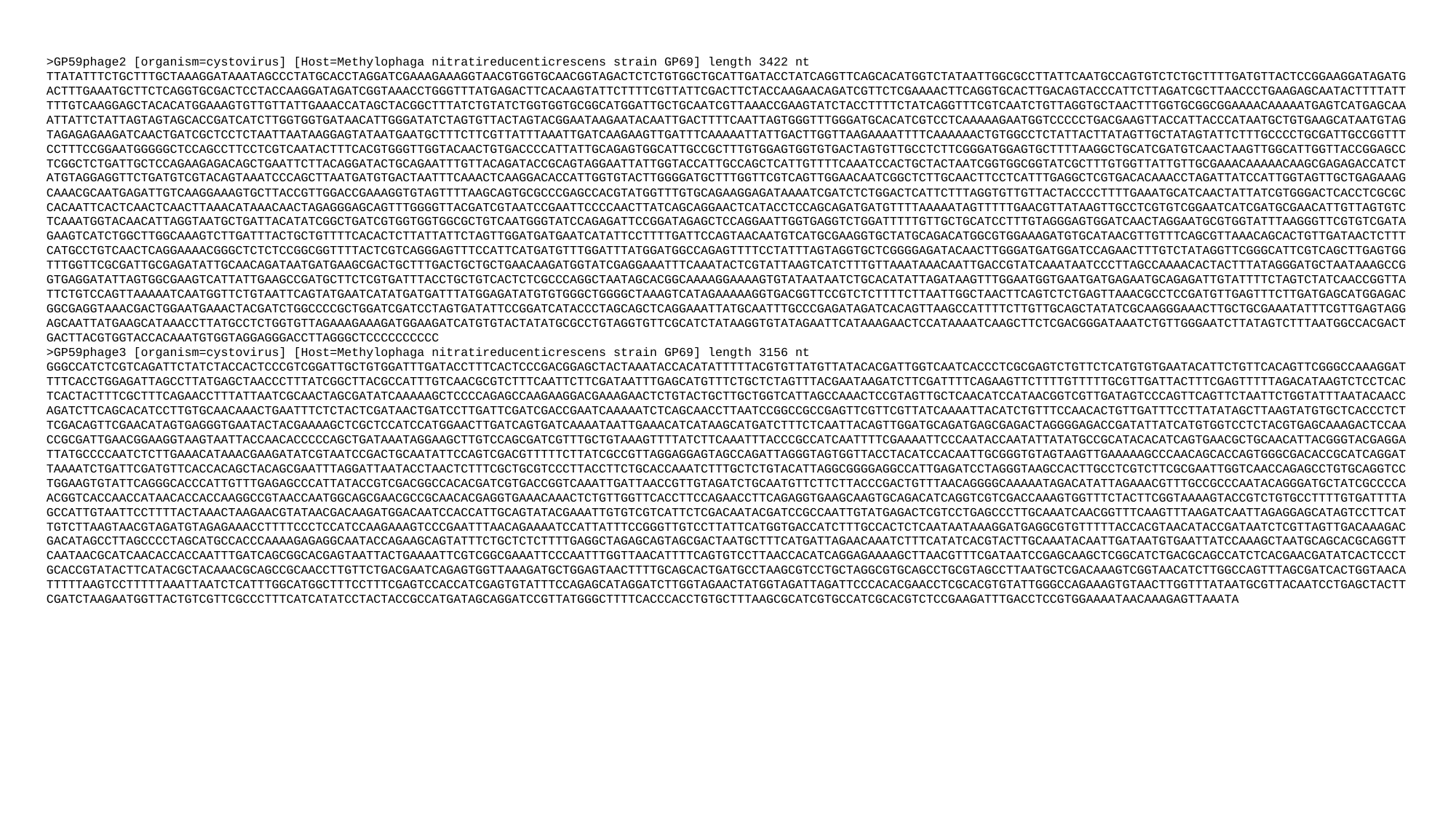

>GP59phage2 [organism=cystovirus] [Host=Methylophaga nitratireducenticrescens strain GP69] length 3422 nt
TTATATTTCTGCTTTGCTAAAGGATAAATAGCCCTATGCACCTAGGATCGAAAGAAAGGTAACGTGGTGCAACGGTAGACTCTCTGTGGCTGCATTGATACCTATCAGGTTCAGCACATGGTCTATAATTGGCGCCTTATTCAATGCCAGTGTCTCTGCTTTTGATGTTACTCCGGAAGGATAGATGACTTTGAAATGCTTCTCAGGTGCGACTCCTACCAAGGATAGATCGGTAAACCTGGGTTTATGAGACTTCACAAGTATTCTTTTCGTTATTCGACTTCTACCAAGAACAGATCGTTCTCGAAAACTTCAGGTGCACTTGACAGTACCCATTCTTAGATCGCTTAACCCTGAAGAGCAATACTTTTATTTTTGTCAAGGAGCTACACATGGAAAGTGTTGTTATTGAAACCATAGCTACGGCTTTATCTGTATCTGGTGGTGCGGCATGGATTGCTGCAATCGTTAAACCGAAGTATCTACCTTTTCTATCAGGTTTCGTCAATCTGTTAGGTGCTAACTTTGGTGCGGCGGAAAACAAAAATGAGTCATGAGCAAATTATTCTATTAGTAGTAGCACCGATCATCTTGGTGGTGATAACATTGGGATATCTAGTGTTACTAGTACGGAATAAGAATACAATTGACTTTTCAATTAGTGGGTTTGGGATGCACATCGTCCTCAAAAAGAATGGTCCCCCTGACGAAGTTACCATTACCCATAATGCTGTGAAGCATAATGTAGTAGAGAGAAGATCAACTGATCGCTCCTCTAATTAATAAGGAGTATAATGAATGCTTTCTTCGTTATTTAAATTGATCAAGAAGTTGATTTCAAAAATTATTGACTTGGTTAAGAAAATTTTCAAAAAACTGTGGCCTCTATTACTTATAGTTGCTATAGTATTCTTTGCCCCTGCGATTGCCGGTTTCCTTTCCGGAATGGGGGCTCCAGCCTTCCTCGTCAATACTTTCACGTGGGTTGGTACAACTGTGACCCCATTATTGCAGAGTGGCATTGCCGCTTTGTGGAGTGGTGTGACTAGTGTTGCCTCTTCGGGATGGAGTGCTTTTAAGGCTGCATCGATGTCAACTAAGTTGGCATTGGTTACCGGAGCCTCGGCTCTGATTGCTCCAGAAGAGACAGCTGAATTCTTACAGGATACTGCAGAATTTGTTACAGATACCGCAGTAGGAATTATTGGTACCATTGCCAGCTCATTGTTTTCAAATCCACTGCTACTAATCGGTGGCGGTATCGCTTTGTGGTTATTGTTGCGAAACAAAAACAAGCGAGAGACCATCTATGTAGGAGGTTCTGATGTCGTACAGTAAATCCCAGCTTAATGATGTGACTAATTTCAAACTCAAGGACACCATTGGTGTACTTGGGGATGCTTTGGTTCGTCAGTTGGAACAATCGGCTCTTGCAACTTCCTCATTTGAGGCTCGTGACACAAACCTAGATTATCCATTGGTAGTTGCTGAGAAAGCAAACGCAATGAGATTGTCAAGGAAAGTGCTTACCGTTGGACCGAAAGGTGTAGTTTTAAGCAGTGCGCCCGAGCCACGTATGGTTTGTGCAGAAGGAGATAAAATCGATCTCTGGACTCATTCTTTAGGTGTTGTTACTACCCCTTTTGAAATGCATCAACTATTATCGTGGGACTCACCTCGCGCCACAATTCACTCAACTCAACTTAAACATAAACAACTAGAGGGAGCAGTTTGGGGTTACGATCGTAATCCGAATTCCCCAACTTATCAGCAGGAACTCATACCTCCAGCAGATGATGTTTTAAAAATAGTTTTTGAACGTTATAAGTTGCCTCGTGTCGGAATCATCGATGCGAACATTGTTAGTGTCTCAAATGGTACAACATTAGGTAATGCTGATTACATATCGGCTGATCGTGGTGGTGGCGCTGTCAATGGGTATCCAGAGATTCCGGATAGAGCTCCAGGAATTGGTGAGGTCTGGATTTTTGTTGCTGCATCCTTTGTAGGGAGTGGATCAACTAGGAATGCGTGGTATTTAAGGGTTCGTGTCGATAGAAGTCATCTGGCTTGGCAAAGTCTTGATTTACTGCTGTTTTCACACTCTTATTATTCTAGTTGGATGATGAATCATATTCCTTTTGATTCCAGTAACAATGTCATGCGAAGGTGCTATGCAGACATGGCGTGGAAAGATGTGCATAACGTTGTTTCAGCGTTAAACAGCACTGTTGATAACTCTTTCATGCCTGTCAACTCAGGAAAACGGGCTCTCTCCGGCGGTTTTACTCGTCAGGGAGTTTCCATTCATGATGTTTGGATTTATGGATGGCCAGAGTTTTCCTATTTAGTAGGTGCTCGGGGAGATACAACTTGGGATGATGGATCCAGAACTTTGTCTATAGGTTCGGGCATTCGTCAGCTTGAGTGGTTTGGTTCGCGATTGCGAGATATTGCAACAGATAATGATGAAGCGACTGCTTTGACTGCTGCTGAACAAGATGGTATCGAGGAAATTTCAAATACTCGTATTAAGTCATCTTTGTTAAATAAACAATTGACCGTATCAAATAATCCCTTAGCCAAAACACTACTTTATAGGGATGCTAATAAAGCCGGTGAGGATATTAGTGGCGAAGTCATTATTGAAGCCGATGCTTCTCGTGATTTACCTGCTGTCACTCTCGCCCAGGCTAATAGCACGGCAAAAGGAAAAGTGTATAATAATCTGCACATATTAGATAAGTTTGGAATGGTGAATGATGAGAATGCAGAGATTGTATTTTCTAGTCTATCAACCGGTTATTCTGTCCAGTTAAAAATCAATGGTTCTGTAATTCAGTATGAATCATATGATGATTTATGGAGATATGTGTGGGCTGGGGCTAAAGTCATAGAAAAAGGTGACGGTTCCGTCTCTTTTCTTAATTGGCTAACTTCAGTCTCTGAGTTAAACGCCTCCGATGTTGAGTTTCTTGATGAGCATGGAGACGGCGAGGTAAACGACTGGAATGAAACTACGATCTGGCCCCGCTGGATCGATCCTAGTGATATTCCGGATCATACCCTAGCAGCTCAGGAAATTATGCAATTTGCCCGAGATAGATCACAGTTAAGCCATTTTCTTGTTGCAGCTATATCGCAAGGGAAACTTGCTGCGAAATATTTCGTTGAGTAGGAGCAATTATGAAGCATAAACCTTATGCCTCTGGTGTTAGAAAGAAAGATGGAAGATCATGTGTACTATATGCGCCTGTAGGTGTTCGCATCTATAAGGTGTATAGAATTCATAAAGAACTCCATAAAATCAAGCTTCTCGACGGGATAAATCTGTTGGGAATCTTATAGTCTTTAATGGCCACGACTGACTTACGTGGTACCACAAATGTGGTAGGAGGGACCTTAGGGCTCCCCCCCCCC
>GP59phage3 [organism=cystovirus] [Host=Methylophaga nitratireducenticrescens strain GP69] length 3156 nt
GGGCCATCTCGTCAGATTCTATCTACCACTCCCGTCGGATTGCTGTGGATTTGATACCTTTCACTCCCGACGGAGCTACTAAATACCACATATTTTTACGTGTTATGTTATACACGATTGGTCAATCACCCTCGCGAGTCTGTTCTCATGTGTGAATACATTCTGTTCACAGTTCGGGCCAAAGGATTTTCACCTGGAGATTAGCCTTATGAGCTAACCCTTTATCGGCTTACGCCATTTGTCAACGCGTCTTTCAATTCTTCGATAATTTGAGCATGTTTCTGCTCTAGTTTACGAATAAGATCTTCGATTTTCAGAAGTTCTTTTGTTTTTGCGTTGATTACTTTCGAGTTTTTAGACATAAGTCTCCTCACTCACTACTTTCGCTTTCAGAACCTTTATTAATCGCAACTAGCGATATCAAAAAGCTCCCCAGAGCCAAGAAGGACGAAAGAACTCTGTACTGCTTGCTGGTCATTAGCCAAACTCCGTAGTTGCTCAACATCCATAACGGTCGTTGATAGTCCCAGTTCAGTTCTAATTCTGGTATTTAATACAACCAGATCTTCAGCACATCCTTGTGCAACAAACTGAATTTCTCTACTCGATAACTGATCCTTGATTCGATCGACCGAATCAAAAATCTCAGCAACCTTAATCCGGCCGCCGAGTTCGTTCGTTATCAAAATTACATCTGTTTCCAACACTGTTGATTTCCTTATATAGCTTAAGTATGTGCTCACCCTCTTCGACAGTTCGAACATAGTGAGGGTGAATACTACGAAAAGCTCGCTCCATCCATGGAACTTGATCAGTGATCAAAATAATTGAAACATCATAAGCATGATCTTTCTCAATTACAGTTGGATGCAGATGAGCGAGACTAGGGGAGACCGATATTATCATGTGGTCCTCTACGTGAGCAAAGACTCCAACCGCGATTGAACGGAAGGTAAGTAATTACCAACACCCCCAGCTGATAAATAGGAAGCTTGTCCAGCGATCGTTTGCTGTAAAGTTTTATCTTCAAATTTACCCGCCATCAATTTTCGAAAATTCCCAATACCAATATTATATGCCGCATACACATCAGTGAACGCTGCAACATTACGGGTACGAGGATTATGCCCCAATCTCTTGAAACATAAACGAAGATATCGTAATCCGACTGCAATATTCCAGTCGACGTTTTTCTTATCGCCGTTAGGAGGAGTAGCCAGATTAGGGTAGTGGTTACCTACATCCACAATTGCGGGTGTAGTAAGTTGAAAAAGCCCAACAGCACCAGTGGGCGACACCGCATCAGGATTAAAATCTGATTCGATGTTCACCACAGCTACAGCGAATTTAGGATTAATACCTAACTCTTTCGCTGCGTCCCTTACCTTCTGCACCAAATCTTTGCTCTGTACATTAGGCGGGGAGGCCATTGAGATCCTAGGGTAAGCCACTTGCCTCGTCTTCGCGAATTGGTCAACCAGAGCCTGTGCAGGTCCTGGAAGTGTATTCAGGGCACCCATTGTTTGAGAGCCCATTATACCGTCGACGGCCACACGATCGTGACCGGTCAAATTGATTAACCGTTGTAGATCTGCAATGTTCTTCTTACCCGACTGTTTAACAGGGGCAAAAATAGACATATTAGAAACGTTTGCCGCCCAATACAGGGATGCTATCGCCCCAACGGTCACCAACCATAACACCACCAAGGCCGTAACCAATGGCAGCGAACGCCGCAACACGAGGTGAAACAAACTCTGTTGGTTCACCTTCCAGAACCTTCAGAGGTGAAGCAAGTGCAGACATCAGGTCGTCGACCAAAGTGGTTTCTACTTCGGTAAAAGTACCGTCTGTGCCTTTTGTGATTTTAGCCATTGTAATTCCTTTTACTAAACTAAGAACGTATAACGACAAGATGGACAATCCACCATTGCAGTATACGAAATTGTGTCGTCATTCTCGACAATACGATCCGCCAATTGTATGAGACTCGTCCTGAGCCCTTGCAAATCAACGGTTTCAAGTTTAAGATCAATTAGAGGAGCATAGTCCTTCATTGTCTTAAGTAACGTAGATGTAGAGAAACCTTTTCCCTCCATCCAAGAAAGTCCCGAATTTAACAGAAAATCCATTATTTCCGGGTTGTCCTTATTCATGGTGACCATCTTTGCCACTCTCAATAATAAAGGATGAGGCGTGTTTTTACCACGTAACATACCGATAATCTCGTTAGTTGACAAAGACGACATAGCCTTAGCCCCTAGCATGCCACCCAAAAGAGAGGCAATACCAGAAGCAGTATTTCTGCTCTCTTTTGAGGCTAGAGCAGTAGCGACTAATGCTTTCATGATTAGAACAAATCTTTCATATCACGTACTTGCAAATACAATTGATAATGTGAATTATCCAAAGCTAATGCAGCACGCAGGTTCAATAACGCATCAACACCACCAATTTGATCAGCGGCACGAGTAATTACTGAAAATTCGTCGGCGAAATTCCCAATTTGGTTAACATTTTCAGTGTCCTTAACCACATCAGGAGAAAAGCTTAACGTTTCGATAATCCGAGCAAGCTCGGCATCTGACGCAGCCATCTCACGAACGATATCACTCCCTGCACCGTATACTTCATACGCTACAAACGCAGCCGCAACCTTGTTCTGACGAATCAGAGTGGTTAAAGATGCTGGAGTAACTTTTGCAGCACTGATGCCTAAGCGTCCTGCTAGGCGTGCAGCCTGCGTAGCCTTAATGCTCGACAAAGTCGGTAACATCTTGGCCAGTTTAGCGATCACTGGTAACATTTTTAAGTCCTTTTTAAATTAATCTCATTTGGCATGGCTTTCCTTTCGAGTCCACCATCGAGTGTATTTCCAGAGCATAGGATCTTGGTAGAACTATGGTAGATTAGATTCCCACACGAACCTCGCACGTGTATTGGGCCAGAAAGTGTAACTTGGTTTATAATGCGTTACAATCCTGAGCTACTTCGATCTAAGAATGGTTACTGTCGTTCGCCCTTTCATCATATCCTACTACCGCCATGATAGCAGGATCCGTTATGGGCTTTTCACCCACCTGTGCTTTAAGCGCATCGTGCCATCGCACGTCTCCGAAGATTTGACCTCCGTGGAAAATAACAAAGAGTTAAATA

## Slide 3
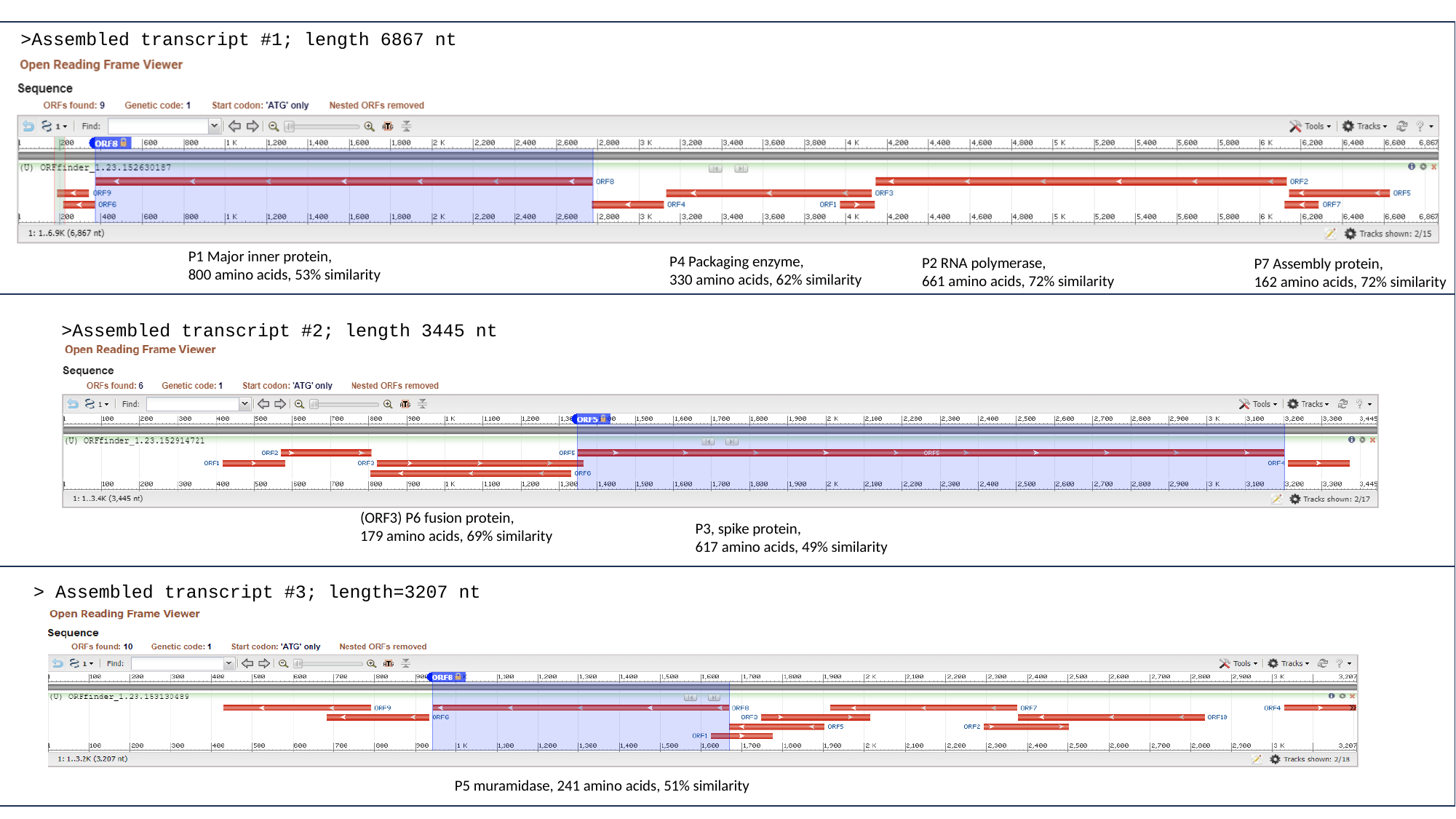

>Assembled transcript #1; length 6867 nt
P1 Major inner protein,
800 amino acids, 53% similarity
P4 Packaging enzyme,
330 amino acids, 62% similarity
P2 RNA polymerase,
661 amino acids, 72% similarity
P7 Assembly protein,
162 amino acids, 72% similarity
>Assembled transcript #2; length 3445 nt
(ORF3) P6 fusion protein,
179 amino acids, 69% similarity
P3, spike protein,
617 amino acids, 49% similarity
> Assembled transcript #3; length=3207 nt
P5 muramidase, 241 amino acids, 51% similarity

## Slide 4
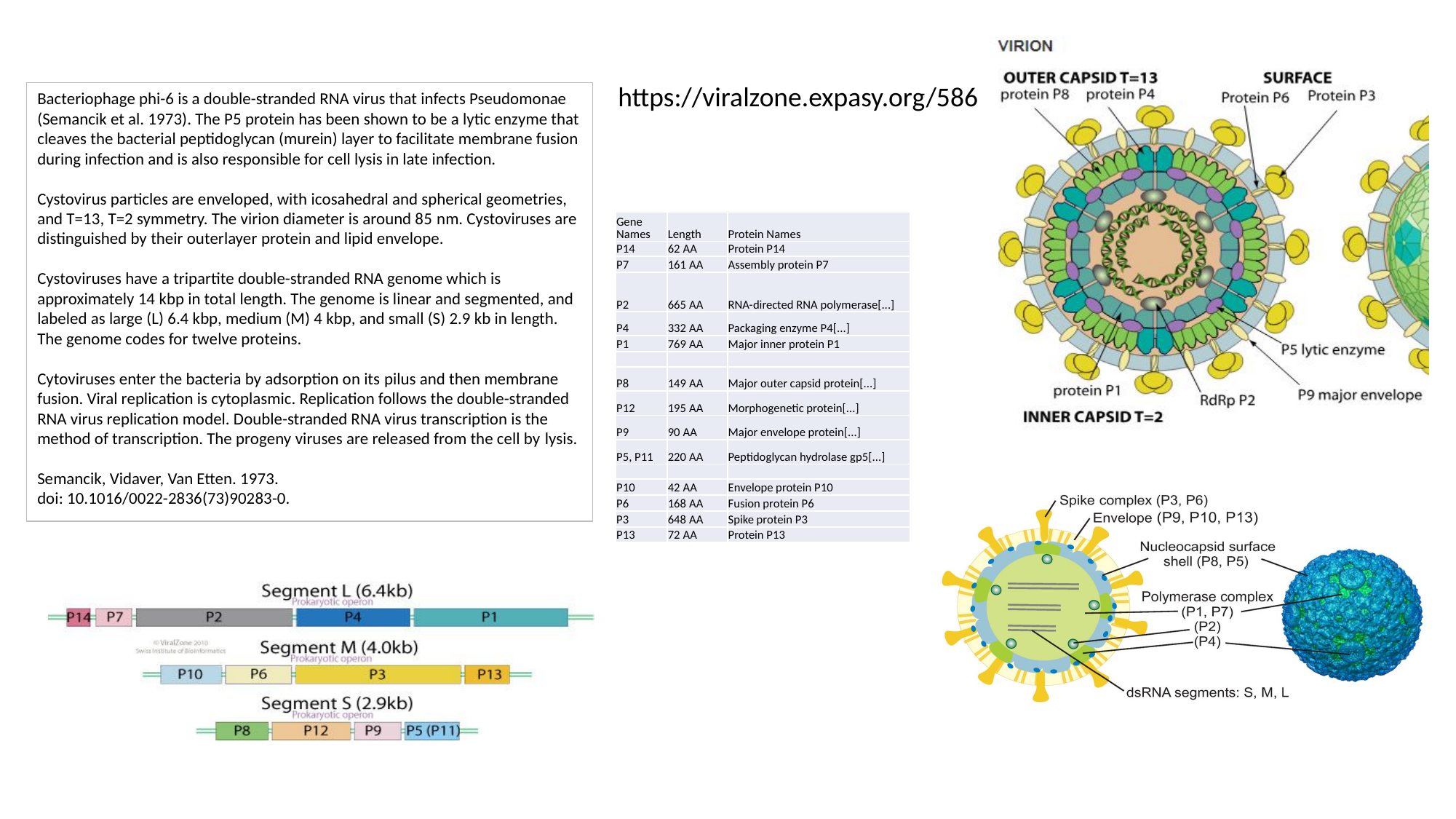

https://viralzone.expasy.org/586
Bacteriophage phi-6 is a double-stranded RNA virus that infects Pseudomonae (Semancik et al. 1973). The P5 protein has been shown to be a lytic enzyme that cleaves the bacterial peptidoglycan (murein) layer to facilitate membrane fusion during infection and is also responsible for cell lysis in late infection.
Cystovirus particles are enveloped, with icosahedral and spherical geometries, and T=13, T=2 symmetry. The virion diameter is around 85 nm. Cystoviruses are distinguished by their outerlayer protein and lipid envelope.
Cystoviruses have a tripartite double-stranded RNA genome which is approximately 14 kbp in total length. The genome is linear and segmented, and labeled as large (L) 6.4 kbp, medium (M) 4 kbp, and small (S) 2.9 kb in length. The genome codes for twelve proteins.
Cytoviruses enter the bacteria by adsorption on its pilus and then membrane fusion. Viral replication is cytoplasmic. Replication follows the double-stranded RNA virus replication model. Double-stranded RNA virus transcription is the method of transcription. The progeny viruses are released from the cell by lysis.
Semancik, Vidaver, Van Etten. 1973.
doi: 10.1016/0022-2836(73)90283-0.
| Gene Names | Length | Protein Names |
| --- | --- | --- |
| P14 | 62 AA | Protein P14 |
| P7 | 161 AA | Assembly protein P7 |
| P2 | 665 AA | RNA-directed RNA polymerase[...] |
| P4 | 332 AA | Packaging enzyme P4[...] |
| P1 | 769 AA | Major inner protein P1 |
| | | |
| P8 | 149 AA | Major outer capsid protein[...] |
| P12 | 195 AA | Morphogenetic protein[...] |
| P9 | 90 AA | Major envelope protein[...] |
| P5, P11 | 220 AA | Peptidoglycan hydrolase gp5[...] |
| | | |
| P10 | 42 AA | Envelope protein P10 |
| P6 | 168 AA | Fusion protein P6 |
| P3 | 648 AA | Spike protein P3 |
| P13 | 72 AA | Protein P13 |
